# Supplementary material for: Parametric estimation of P(X > Y) for normal distributions in the context of probabilistic environmental risk assessment
Source: PeerJ. 2015 Aug 18;3:e1164. doi: 10.7717/peerj.1164 (PMC4548477; doi:10.7717/peerj.1164)
Supplement: Supplemental Information 1 [file peerj-03-1164-s001.pdf]

Supplementary Material: Parametric estimation of $P(X > Y)$ for normal distributions in the context of probabilistic environmental risk assessment

Rianne Jacobs, Andriëtte Bekker, Hilko van der Voet, Cajo ter Braak

Contents

1 R-code **4**
1.1 Functions . . . 4
1.1.1 pnorm1 . . . 4
1.1.2 mode_f . . . 4
1.1.3 R_MLE_f . . . 4
1.1.4 R_QMLE_f . . . 4
1.1.5 R_NP_f . . . 5
1.1.6 comp_sampler_f . . . 5
1.1.7 R_boot_f . . . 5
1.1.8 R_bootstrap_f . . . 6
1.2 Main Program . . . 6
1.3 Figures . . . 10
1.3.1 Coverage Figures . . . 10
1.3.2 Sampling distribution of estimators . . . 14
1.3.3 Bayes posterior summary statistics . . . 16

2 Figures and Tables **21**
2.1 Application . . . 21
2.2 Case: $\sigma_y = \sigma_x$ . . . 24
2.3 Case: $\sigma_y = 0.2\sigma_x$ . . . 36
2.4 Case: $\sigma_y = 5\sigma_x$ . . . 48

List of Figures

S1 Histograms of the ratio of the 95% upper bound to the upper bound of the 90% credible interval of the Bayesian estimator for different values of the true R and sample sizes $n_x = 100$ and $n_y = 12$. The vertical red line indicates the corresponding ratio in the case study. . . . 21
S2 Histograms of the distances between the 95% upper bound and the true R value of the QMLE (left column) and Bayesian estimator (right column) for different values of the true R and sample sizes $n_x = 100$ and $n_y = 12$. The vertical red line indicates the corresponding upper bounds in the case study. . . . 22
S3 Histograms of the ratio of the 95% upper bound of the Bayesian estimator to QMLE for different values of the true R and sample sizes $n_x = 100$ and $n_y = 12$. The vertical red line indicates the corresponding ratio in the case study. . . . 23
S4 The median of the sampling distribution of the three Bayesian point estimators (mean, median and mode) calculated on the original scale and plotted on log_{10}-scale. When plotted on log_{10}-scale, a zero mode becomes $-\infty$. The diagonal dotted line represents the values where $\hat{R} = R$. 24
S5 The 0.025 and 0.975 quantiles of the sampling distribution of the three Bayesian point estimators (mean, median and mode) calculated on the original scale and plotted on log_{10}-scale. When plotted on log_{10}-scale, a zero mode becomes $-\infty$. The diagonal dotted line represents the values where $\hat{R} = R$. . . . 25
S6 The median of the sampling distribution of the three Bayesian point estimators (mean, median and mode) calculated on the probit scale. The diagonal dotted line represents the values where $probit(\hat{R}) = probit(R)$. . . . 26
S7 The 0.025 and 0.975 quantiles of the sampling distribution of the three Bayesian point estimators (mean, median and mode) calculated on the probit scale. The diagonal dotted line represents the values where $probit(\hat{R}) = probit(R)$. . . . 27
S8 The median of the sampling distribution of the four point estimators, $\hat{R}_{MLE}$, $\hat{R}_{QMLE}$, $\hat{R}_{Bayes}$ and $\hat{R}_{NP}$ calculated on the probit scale. The diagonal dotted line represents the values where $probit(\hat{R}) = probit(R)$. The horizontal grey line gives the lower bound ($probit\left(\frac{1}{n_x n_y + 2}\right)$) of $probit(\hat{R}_{NP})$. . . . 28
S9 The 0.025 and 0.975 quantiles of the sampling distribution of the four point estimators, $\hat{R}_{MLE}$, $\hat{R}_{QMLE}$, $\hat{R}_{Bayes}$ and $\hat{R}_{NP}$ calculated on the probit scale. The diagonal dotted line represents the values where $probit(\hat{R}) = probit(R)$. The horizontal grey line gives the lower bound ($probit\left(\frac{1}{n_x n_y + 2}\right)$) of $probit(\hat{R}_{NP})$. . . . 29

S10 Scatterplots of the 90% two-sided coverage probabilities against the relative median interval length calculated on the probit scale. The value of the true R value is illustrated by the color scale. The size of the dots corresponds to the size of the sample size of effect. A vertical reference line is drawn at a coverage probability of 90%. The points corresponding to $n_y = 12$ are indicated by an open black circle. . . . 30

S11 Scatterplots of the 95% one-sided coverage probabilities against the relative median difference calculated on the probit scale. The value of the true R value is illustrated by the color scale. The size of the dots corresponds to the size of the sample size of effect. A vertical reference line is drawn at a coverage probability of 95%. The points corresponding to $n_y = 12$ are indicated by a black circle. . . . 31

S12 The median of the sampling distribution of the three Bayesian point estimators (mean, median and mode) calculated on the original scale and plotted on log_{10}-scale. When plotted on log_{10}-scale, a zero mode becomes $-\infty$. The diagonal dotted line represents the values where $\hat{R} = R$. 36

S13 The 0.025 and 0.975 quantiles of the sampling distribution of the three Bayesian point estimators (mean, median and mode) calculated on the original scale and plotted on log_{10}-scale. When plotted on log_{10}-scale, a zero mode becomes $-\infty$. The diagonal dotted line represents the values where $\hat{R} = R$. . . . 37

S14 The median of the sampling distribution of the three Bayesian point estimators (mean, median and mode) calculated on the probit scale. The diagonal dotted line represents the values where $probit(\hat{R}) = probit(R)$. . . . 38

S15 The 0.025 and 0.975 quantiles of the sampling distribution of the three Bayesian point estimators (mean, median and mode) calculated on the probit scale. The diagonal dotted line represents the values where $probit(\hat{R}) = probit(R)$. . . . 39

S16 The median of the sampling distribution of the four point estimators, $\hat{R}_{MLE}$, $\hat{R}_{QMLE}$, $\hat{R}_{Bayes}$ and $\hat{R}_{NP}$ calculated on the probit scale. The diagonal dotted line represents the values where $probit(\hat{R}) = probit(R)$. The horizontal grey line gives the lower bound ($probit\left(\frac{1}{n_x n_y+2}\right)$) of $probit(\hat{R}_{NP})$. . . . 40

S17 The 0.025 and 0.975 quantiles of the sampling distribution of the four point estimators, $\hat{R}_{MLE}$, $\hat{R}_{QMLE}$, $\hat{R}_{Bayes}$ and $\hat{R}_{NP}$ calculated on the probit scale. The diagonal dotted line represents the values where $probit(\hat{R}) = probit(R)$. The horizontal grey line gives the lower bound ($probit\left(\frac{1}{n_x n_y+2}\right)$) of $probit(\hat{R}_{NP})$. . . . 41

S18 Scatterplots of the 90% two-sided coverage probabilities against the relative median interval length calculated on the probit scale. The value of the true R value is illustrated by the color scale. The size of the dots corresponds to the size of the sample size of effect. A vertical reference line is drawn at a coverage probability of 90%. The points corresponding to $n_y = 12$ are indicated by an open black circle. . . . 42

S19 Scatterplots of the 95% one-sided coverage probabilities against the relative median difference calculated on the probit scale. The value of the true R value is illustrated by the color scale. The size of the dots corresponds to the size of the sample size of effect. A vertical reference line is drawn at a coverage probability of 95%. The points corresponding to $n_y = 12$ are indicated by a black circle. . . . 43

S20 The median of the sampling distribution of the three Bayesian point estimators (mean, median and mode) calculated on the original scale and plotted on log_{10}-scale. When plotted on log_{10}-scale, a zero mode becomes $-\infty$. The diagonal dotted line represents the values where $\hat{R} = R$. 48

S21 The 0.025 and 0.975 quantiles of the sampling distribution of the three Bayesian point estimators (mean, median and mode) calculated on the original scale and plotted on log_{10}-scale. When plotted on log_{10}-scale, a zero mode becomes $-\infty$. The diagonal dotted line represents the values where $\hat{R} = R$. . . . 49

S22 The median of the sampling distribution of the three Bayesian point estimators (mean, median and mode) calculated on the probit scale. The diagonal dotted line represents the values where $probit(\hat{R}) = probit(R)$. . . . 50

S23 The 0.025 and 0.975 quantiles of the sampling distribution of the three Bayesian point estimators (mean, median and mode) calculated on the probit scale. The diagonal dotted line represents the values where $probit(\hat{R}) = probit(R)$. . . . 51

S24 The median of the sampling distribution of the four point estimators, $\hat{R}_{MLE}$, $\hat{R}_{QMLE}$, $\hat{R}_{Bayes}$ and $\hat{R}_{NP}$ calculated on the probit scale. The diagonal dotted line represents the values where $probit(\hat{R}) = probit(R)$. The horizontal grey line gives the lower bound ($probit\left(\frac{1}{n_x n_y+2}\right)$) of $probit(\hat{R}_{NP})$. . . . 52

S25 The 0.025 and 0.975 quantiles of the sampling distribution of the four point estimators, $\hat{R}_{MLE}$, $\hat{R}_{QMLE}$, $\hat{R}_{Bayes}$ and $\hat{R}_{NP}$ calculated on the probit scale. The diagonal dotted line represents the values where $probit(\hat{R}) = probit(R)$. The horizontal grey line gives the lower bound ($probit\left(\frac{1}{n_x n_y+2}\right)$) of $probit(\hat{R}_{NP})$. . . . 53

S26 Scatterplots of the 90% two-sided coverage probabilities against the relative median interval length calculated on the probit scale. The value of the true R value is illustrated by the color scale. The size of the dots corresponds to the size of the sample size of effect. A vertical reference line is drawn at a coverage probability of 90%. The points corresponding to $n_y = 12$ are indicated by an open black circle. 54
S27 Scatterplots of the 95% one-sided coverage probabilities against the relative median difference calculated on the probit scale. The value of the true R value is illustrated by the color scale. The size of the dots corresponds to the size of the sample size of effect. A vertical reference line is drawn at a coverage probability of 95%. The points corresponding to $n_y = 12$ are indicated by a black circle. 55

List of Tables

S1 Test statistic values and p-values for six normality tests on the nano-Ag exposure and effect concentration data of Gottschalk et al. (2013). 21
S2 The sample size, true R, true $probit(R)$, mean ($\bar{\hat{R}}$), standard deviation (SD), bias and root mean squared error (RMSE) of the MLE for all sample size scenarios and selected R values. . 32
S3 The sample size, true R, true $probit(R)$, mean ($\bar{\hat{R}}$), standard deviation (SD), bias and root mean squared error (RMSE) of the QMLE for all sample size scenarios and selected R values. 33
S4 The sample size, true R, true $probit(R)$, mean ($\bar{\hat{R}}$), standard deviation (SD), bias and root mean squared error (RMSE) of the Bayes estimator for all sample size scenarios and selected R values. 34
S5 The sample size, true R, true $probit(R)$, mean ($\bar{\hat{R}}$), standard deviation (SD), bias and root mean squared error (RMSE) of the non-parametric estimator for all sample size scenarios and selected R values. 35
S6 The sample size, true R, true $probit(R)$, mean ($\bar{\hat{R}}$), standard deviation (SD), bias and root mean squared error (RMSE) of the MLE for all sample size scenarios and selected R values. . 44
S7 The sample size, true R, true $probit(R)$, mean ($\bar{\hat{R}}$), standard deviation (SD), bias and root mean squared error (RMSE) of the QMLE for all sample size scenarios and selected R values. 45
S8 The sample size, true R, true $probit(R)$, mean ($\bar{\hat{R}}$), standard deviation (SD), bias and root mean squared error (RMSE) of the Bayes estimator for all sample size scenarios and selected R values. 46
S9 The sample size, true R, true $probit(R)$, mean ($\bar{\hat{R}}$), standard deviation (SD), bias and root mean squared error (RMSE) of the non-parametric estimator for all sample size scenarios and selected R values. 47
S10 The sample size, true R, true $probit(R)$, mean ($\bar{\hat{R}}$), standard deviation (SD), bias and root mean squared error (RMSE) of the MLE for all sample size scenarios and selected R values. . 56
S11 The sample size, true R, true $probit(R)$, mean ($\bar{\hat{R}}$), standard deviation (SD), bias and root mean squared error (RMSE) of the QMLE for all sample size scenarios and selected R values. 57
S12 The sample size, true R, true $probit(R)$, mean ($\bar{\hat{R}}$), standard deviation (SD), bias and root mean squared error (RMSE) of the Bayes estimator for all sample size scenarios and selected R values. 58
S13 The sample size, true R, true $probit(R)$, mean ($\bar{\hat{R}}$), standard deviation (SD), bias and root mean squared error (RMSE) of the non-parametric estimator for all sample size scenarios and selected R values. 59

1 R-code

This section contains the R codes used to perform the simulation. It consists of the main simulation program and the user-defined functions used in the main program.

1.1 Functions

1.1.1 pnorm1

Function to determine scale of estimator - original or probit

```
pnorm1 <- function(x){
   #original
    #newR = pnorm(x)
   #probit
    newR = x
   newR
}
```

1.1.2 mode_f

Function to estimate mode

```
mode_f <- function(x) {
  dens = try(density(x),silent=TRUE)
  if (class(dens)=="try-error") mode=0 else
   mode = dens$x[which.max(dens$y)]
  if (mode<0) mode=0 #only to use on on original scale
  mode
}
```

1.1.3 R_MLE_f

Function that calculates the MLE of $P(X > Y)$ for the normal-normal model

```
R_MLE_f <- function(x,y,c){
  if (class(x) == "matrix") {
    nx = nrow(x)
    ny = nrow(y)
    m1 = apply(x,2,mean)
    m2 = apply(y,2,mean)
    var1 = apply(x,2,var)
    var2 = apply(y,2,var)
  } else {
    nx = length(x)
    ny = length(y)
    m1 = mean(x)
    m2 = mean(y)
    var1 = var(x)
    var2 = var(y)
  }
  theta = (m1 - m2 - c)/ sqrt(var1/nx*(nx-1) + var2/ny*(ny-1))
  R_MLE = pnorm1(theta)
  R_MLE
}
```

1.1.4 R_QMLE_f

Function that calculates the QMLE of $P(X > Y)$ for the normal-normal model

```
R_QMLE_f <- function(x,y,c){
  if (class(x) == "matrix") {
    m1 = apply(x,2,mean)
    m2 = apply(y,2,mean)
    var1 = apply(x,2,var)
    var2 = apply(y,2,var)
  } else {
    m1 = mean(x)
    m2 = mean(y)
```

```
    var1 = var(x)
    var2 = var(y)
  }
  theta = (m1 - m2 - c)/ sqrt(var1 + var2)
  R_QMLE = pnorm1(theta)
  R_QMLE
}
```

1.1.5 R_NP_f

Function that calculates the non-parametric estimator of $P(X > Y)$

```
R_NP_f <- function(exp,sen,c){
  nx = length(exp)
  ny = length(sen)
  vec = rep(NA,length(c))
  for (i in 1:length(c)) {
    x_y = outer(exp,(sen+c[i]), "-")
    gt=sum(x_y>1e-15)
    eq = sum(abs(x_y)< 1e-15) # ties
    r = (gt + 0.5*eq + 1)/(nx*ny + 2)
    vec[i] = qnorm(r)
  }
  vec
}
```

1.1.6 comp_sampler_f

Function that generates sample of size n from posterior distribution of R and calculates the HPD credible intervals of posterior sample

```
library(coda)
comp_sampler_f <- function (n, seed, xbar, ybar, n_x, n_y, a_x, a_y, b_x, b_y,c,p2,p1) {
  B = length(xbar)
  samples = matrix(NA,nrow = n, ncol = B)
  int2 = matrix(NA,nrow = B, ncol = 2)
  int1 = matrix(NA,nrow = B, ncol = 1)
  set.seed(seed)
  for (i in 1:B) {
    bx = b_x[i]
    by = b_y[i]
    xmean = xbar[i]
    ymean = ybar[i]
    s2x = 1/rgamma(n, shape = a_x, rate = bx)
    s2y = 1/rgamma(n, shape = a_y, rate = by)
    mx = rnorm(n, xmean, sqrt(s2x/n_x))
    my = rnorm(n, ymean, sqrt(s2y/n_y))
    r = pnorm1((mx - my - c) / sqrt(s2x + s2y))
    samples[,i] = r
    #calculate 2-sided HPD interval
     int2[i,] = HPDinterval(mcmc(r),prob = p2)
    #calculate 1-sided percentile interval
     int1[i] = quantile(r,probs=p1)
  }
  return(list(samples = samples,intervals2 = int2,intervals1 = int1))
}
```

1.1.7 R_boot_f

Function to pass to statistic in boot to manually generate parametric bootstrap for R

```
R_boot_f <- function(x,i,nx,ny,m1,m2,var1,var2,c,func){
  exp_new = rnorm(nx,m1,sqrt(var1))
  sen_new = rnorm(ny,m2,sqrt(var2))
  R = func(exp_new,sen_new,c)
  R
}
```

1.1.8 R_bootstrap_f

Function to pass to statistic in boot to generate non-parametric bootstrap for R

```
R_bootstrap_f <- function(data,i,func,nx,ny,c){
  newdata = data[i]
  exp = newdata[1:nx]
  sen = newdata[(nx+1):(nx+ny)]
  r = func(exp,sen,c)
  r
}
```

1.2 Main Program

```
library(boot)
library(coda)

#version of code
 vers = "v10"
#sigma case
 factor = 1

#Parameter choices
 n_x = rep(c(5,100),each=5)
 n_y = rep(c(2,5,12,20,100),2)
 mu_x = -2.948
 mu_y = -2.948
 sig2_x = 0.0738
 sig2_y = (factor*sqrt(0.0738))^2

 n_scen = length(n_x)
B = 4500 #number of simulations
n_boot = 1000 #number of bootstrap iterations

p2 = 0.9 #confidence level for 2-sided CI
p1 = 0.95 #confidence level for 1-sided CI

#mean difference producing range of R values
 if (factor == 1)
   c = seq(0,2.9,0.1) else
   if (factor == 5)
     c = seq(0,10.4,0.4) else
     if (factor == 0.2)
       c = seq(0,2.1,0.1)

R_true = pnorm1((mu_x - mu_y - c)/sqrt(sig2_x + sig2_y))

#starting seed list for MC simulations for each scenario
 set.seed(1,kind="Mersenne-Twister")
 seed_list1 = sample(4:1e8,n_scen)

#starting seed list for bootstraps for each scenarios
 set.seed(2,kind="Mersenne-Twister")
 seed_list2 = sample(4:1e8,n_scen)

#starting seed list for MCMC for each scenario
 set.seed(3,kind="Mersenne-Twister")
 seed_list3 = sample(4:1e8,n_scen)

#-----------------------------------------------------------------------
#  Generating B exposure and B sensitivity samples for each scenario
#-----------------------------------------------------------------------

for (scen in 1:10){
  set.seed(seed_list1[scen],kind = "Mersenne-Twister") #set a random seed per scenario

  #Parameter values for scen'th scenario
```

```
   nx = n_x[scen]
   ny = n_y[scen]

  #Generating and writing samples
   MC_x = replicate(B,rnorm(nx,mu_x,sqrt(sig2_x)))
   name_x = paste("output_",vers,"/exposure/Scenario_",scen,
                  "_Exposure.RData",sep="")
   save(MC_x,file = name_x)

   MC_y = replicate(B,rnorm(ny,mu_y,sqrt(sig2_y)))
   name_y = paste("output_",vers,"/sensitivity_",factor,"/Scenario_",scen,
                  "_Sensitivity.RData",sep="")
   save(MC_y,file = name_y))
}

#----------------------------------------------------------
#                   MLE estimator
#----------------------------------------------------------

bounds = list()
data = rep(1,10) #fake data vector for boot function

for (scen in 1:n_scen){
  set.seed(seed_list2[scen],kind = "Mersenne-Twister")
  exp_name = paste("output_",vers,"/exposure/Scenario_",scen,"_Exposure.RData",sep="")
  sen_name = paste("output_",vers,"/sensitivity_",factor,"/Scenario_",scen,
                   "_Sensitivity.RData",sep="")
  load(exp_name, .GlobalEnv)
  load(sen_name, .GlobalEnv)
  nx = nrow(MC_x)
  ny = nrow(MC_y)
  num = rep(0,length(c))
  R_MLE = matrix(NA,nrow = length(c),ncol = B)
  l_MLE2 = matrix(NA,nrow = length(c),ncol = B)
  u_MLE2 = matrix(NA,nrow = length(c),ncol = B)
  u_MLE1 = matrix(NA,nrow = length(c),ncol = B)

  #loop for confidence intervals
   for (sim in 1:B) {
     m1 = mean(MC_x[,sim])
     m2 = mean(MC_y[,sim])
     var1 = var(MC_x[,sim])
     var2 = var(MC_y[,sim])
     R_MLE[,sim] = pnorm1((m1 - m2 - c)/ sqrt(var1/nx*(nx-1) + var2/ny*(ny-1)))
     s <- function(x,Mhat,deltahat,fhat,prob){
      arg = sqrt(Mhat) * deltahat
      nc = sqrt(Mhat) * x
      pt(arg,df=fhat,ncp=nc) - prob
     }
     Mhat = (var1 + var2)/(var1/nx + var2/ny)
     fhat = (var1 + var2)^2/(var1^2/(nx-1) + var2^2/(ny-1))
     deltahat = (m1 - m2 - c)/(sqrt(var1/nx*(nx-1) + var2/ny*(ny-1)))
     for (i in 1:length(c)) {
       l_MLE2[i,sim] = uniroot.all(s,interval=c(-500,500),Mhat = Mhat,fhat = fhat,
                                 deltahat = deltahat[i],prob = p1)
       u_MLE2[i,sim] = uniroot.all(s,interval=c(-500,500),Mhat = Mhat,fhat = fhat,
                                deltahat = deltahat[i],prob = 1-p1)
     }
   }
  u_MLE1 = u_MLE2
  bca = B-num #number of intervals where BCa was used
  bounds[[scen]] = list(l_MLE2,u_MLE2,u_MLE1,R_MLE,bca)
}
name = paste("output_",vers,"/estimators/MLE_raw_output_",factor,".RData",sep="")
save(bounds,file = name)
```

```
#--------------------------------------------------------
#                     QuasiMLE estimator
#--------------------------------------------------------

bounds = list()
data = rep(1,10) #fake data vector for boot function

for (scen in 1:n_scen){
  set.seed(seed_list2[scen],kind = "Mersenne-Twister")
  exp_name = paste("output_",vers,"/exposure/Scenario_",scen,"_Exposure.RData",sep="")
  sen_name = paste("output_",vers,"/sensitivity_",factor,"/Scenario_",scen,
                   "_Sensitivity.RData",sep="")
  load(exp_name, .GlobalEnv)
  load(sen_name, .GlobalEnv)
  nx = nrow(MC_x)
  ny = nrow(MC_y)
  num = rep(0,length(c))
  R_QMLE = matrix(NA,nrow = length(c),ncol = B)
  l_QMLE2 = matrix(NA,nrow = length(c),ncol = B)
  u_QMLE2 = matrix(NA,nrow = length(c),ncol = B)
  u_QMLE1 = matrix(NA,nrow = length(c),ncol = B)

  #loop for confidence intervals
   for (sim in 1:B) {
     m1 = mean(MC_x[,sim])
     m2 = mean(MC_y[,sim])
     var1 = var(MC_x[,sim])
     var2 = var(MC_y[,sim])
     R_QMLE[,sim] = pnorm1((m1 - m2 - c)/ sqrt(var1 + var2))
     s <- function(x,Mhat,deltahat,fhat,prob){
      arg = sqrt(Mhat) * deltahat
      nc = sqrt(Mhat) * x
      pt(arg,df=fhat,ncp=nc) - prob
     }
     Mhat = (var1 + var2)/(var1/nx + var2/ny)
     fhat = (var1 + var2)^2/(var1^2/(nx-1) + var2^2/(ny-1))
     deltahat = (m1 - m2 - c)/(sqrt(var1 + var2))
     for (i in 1:length(c)) {
       l_QMLE2[i,sim] = uniroot.all(s,interval=c(-500,500),Mhat = Mhat,fhat = fhat,
                                    deltahat = deltahat[i],prob = p1)
       u_QMLE2[i,sim] = uniroot.all(s,interval=c(-500,500),Mhat = Mhat,fhat = fhat,
                                    deltahat = deltahat[i],prob = 1-p1)
     }
   }
  u_QMLE1 = u_QMLE2
  bca = B-num #number of intervals where BCa was used
  bounds[[scen]] = list(l_QMLE2,u_QMLE2,u_QMLE1,R_QMLE,bca)
}
name = paste("output_",vers,"/estimators/QMLE_raw_output_",factor,".RData",sep="")
save(bounds,file = name)

#-----------------------------------------------------------------------
#                      Bayes estimator
#-----------------------------------------------------------------------

n_bayes = 1000 #posterior sample size
bounds = list()

for (scen in 1:n_scen){
  seed = seed_list3[scen]
  exp_name = paste("output_",vers,"/exposure/Scenario_",scen,"_Exposure.RData",sep="")
  sen_name = paste("output_",vers,"/sensitivity_",factor,"/Scenario_",scen,
                   "_Sensitivity.RData",sep="")
  load(exp_name, .GlobalEnv)
  load(sen_name, .GlobalEnv)

```

```
  #sample parameters estimator
   nx = nrow(MC_x)
   ny = nrow(MC_y)
   m_x = apply(MC_x,2,mean)
   m_y = apply(MC_y,2,mean)
   var_x = apply(MC_x,2,var)
   var_y = apply(MC_y,2,var)

  #Bayes posterior parameters
   a_x = 0.5*(nx-1)
   a_y = 0.5*(ny-1)
   b_x = 0.5*(nx-1)*var_x
   b_y = 0.5*(ny-1)*var_y

  R_Bayes_mean = matrix(NA,nrow = length(c),ncol = B)
  R_Bayes_mode = matrix(NA,nrow = length(c),ncol = B)
  R_Bayes_median = matrix(NA,nrow = length(c),ncol = B)
  l_Bayes2 = matrix(NA,nrow = length(c),ncol = B)
  u_Bayes2 = matrix(NA,nrow = length(c),ncol = B)
  u_Bayes1 = matrix(NA,nrow = length(c),ncol = B)
  for (i in 1:length(c)){
    object = comp_sampler_f(n_bayes,seed,m_x, m_y , nx, ny, a_x, a_y, b_x, b_y,c[i], p2,p1)
    s = object$samples
    Post_median = apply(s,2,median)
    Post_mean = apply(s,2,mean)
    Post_mode = apply(s,2,mode_f)
    int2 = object$intervals2
    int1 = object$intervals1
    l_Bayes2[i,] = int2[,1]
    u_Bayes2[i,] = int2[,2]
    u_Bayes1[i,] = int1
    R_Bayes_median[i,] = Post_median
    R_Bayes_mode[i,] = Post_mode
    R_Bayes_mean[i,] = Post_mean
  }
  bounds[[scen]] = list(l_Bayes2,u_Bayes2,u_Bayes1,
                        R_Bayes_median,R_Bayes_mode,R_Bayes_mean)
}
name = paste("output_",vers,"/estimators/Bayes_raw_output_",factor,".RData",sep="")
save(bounds,file = name)

#------------------------------------------
#        Nonparametric estimator
#------------------------------------------

bounds = list()

for (scen in 1:n_scen){
  set.seed(seed_list2[scen],kind = "Mersenne-Twister")
  exp_name = paste("output_",vers,"/exposure/Scenario_",scen,"_Exposure.RData",sep="")
  sen_name = paste("output_",vers,"/sensitivity_",factor,"/Scenario_",scen,
                   "_Sensitivity.RData",sep="")
  load(exp_name, .GlobalEnv)
  load(sen_name, .GlobalEnv)
  nx = nrow(MC_x)
  ny = nrow(MC_y)
  R_NP = matrix(NA,nrow = length(c),ncol = B)
  l_NP2 = matrix(NA,nrow = length(c),ncol = B)
  u_NP2 = matrix(NA,nrow = length(c),ncol = B)
  u_NP1 = matrix(NA,nrow = length(c),ncol = B)
  num = rep(0,length(c))

  #loop for bootstrapping
   for (sim in 1:B) {
     d = c(MC_x[,sim],MC_y[,sim])
     myboot = boot(d,R_bootstrap_f,R=n_boot,strata = c(rep(1,nx),rep(2,ny)),
```

```
                   func = R_NP_f,nx=nx,ny=ny,c = c)
     R_NP[,sim] = myboot$t0
     for (i in 1:length(c)){
       int = try(boot.ci(myboot,conf = p2,type="bca",index=i)$bca[,4:5],silent = TRUE)
       if (!is.numeric(int)) {
         int = try(boot.ci(myboot,conf = p2,type="perc",index=i)$percent[,4:5],silent = TRUE)
         num[i] = num[i] + 1
       }
       if (!is.numeric(int))
         int = c(myboot$t0[i],myboot$t0[i])
       if (is.na(int[2])) int[2] = max(myboot$t[,i])
       if (is.na(int[1])) int[1] = min(myboot$t[,i])
       l_NP2[i,sim] = int[1]
       u_NP2[i,sim] = int[2]
     }
    }
  u_NP1 = u_NP2
  bca = B - num
  bounds[[scen]] = list(l_NP2,u_NP2,u_NP1,R_NP,bca)
}
name = paste("output_",vers,"/estimators/NP_raw_output_",factor,".RData",sep="")
save(bounds,file = name)
```

1.3 Figures

1.3.1 Coverage Figures

This code produces the coverage figures for the 2-sided intervals. The coverage figures for the upper bounds are produced in a similar manner.

```
library(boot) #bootstrap
library(coda) #for HPD intervals
library(xtable) #to convert table to latex code
library(ggplot2) #to use ggplot
library(gridExtra) #for facet spacing
#---------------------------------------------
# file name components
#---------------------------------------------
#version of code
vers = "v10"
#sigma case
factor = 1

#Parameter choices
n_x = rep(c(5,100),each=5)
n_y = rep(c(2,5,12,20,100),2)
mu_x = -2.948
mu_y = -2.948
sig2_x = 0.0738
sig2_y = (factor*sqrt(sig2_x))^2

n_scen = length(n_x)
source("functions/functions.R")

B = 4500 #number of simulations to be performed

#choosing the scale
pnorm1 <- function(x){
  #original
  #newR = pnorm(x)
  #probit
  newR = x
  newR
}

#creating labels for facet graph
mf_labeller <- function(var, value){
```

```
  value <- as.character(value)
  if (var=="nx") {
    value[value==5] <- "\n Exposure sample size: 5\n"
    value[value==100]   <- "\n Exposure sample size: 100\n"
  }
  if (var=="Estimator") {
    value[value=="QMLE_t"] <- "\n QMLE - noncentral t\n"
    value[value=="QMLE_boot"]   <- "\n QMLE - bootstrap\n"
    value[value=="Bayes"] <- "\n Bayesian\n"
    value[value=="NP"]   <- "\n Non-parametric\n"
  }
  return(value)
}

p2 = 0.9 #confidence level for 2-sided CI
p1 = 0.95 #confidence level for 1-sided CI

if (factor == 1)
  #for equal variances
  c = seq(0,2.9,0.1) else #mean difference producing range of R values
    if (factor == 5)
      #for 5x
      c = seq(0,10.4,0.4) else #mean difference producing range of R values
        if (factor == 0.2)
          #for 0.2x
          c = seq(0,2.1,0.1) #mean difference producing range of R values

R_true = pnorm1((mu_x - mu_y - c)/sqrt(sig2_x + sig2_y))
Rmatrix = matrix(R_true,nrow = length(R_true),ncol = B)

#---------------------------------
#   QMLE - noncentral t
#---------------------------------

name = paste("output_",vers,"/estimators/QMLE_raw_output_",factor,"_noncent",
             ".RData",sep="")
load(name)

#Coverage and lengths
coverage = matrix(NA,nrow=length(R_true),ncol=n_scen)
lengths = matrix(NA,nrow=length(R_true),ncol=n_scen)
std_lengths = matrix(NA,nrow=length(R_true),ncol=n_scen)
interval_data = NULL
for (scen in 1:n_scen){
  lower = bounds[[scen]][[1]]
  upper = bounds[[scen]][[2]]
  count = apply((Rmatrix>(lower) & Rmatrix<(upper)),1,sum)
  coverage[,scen] = count/B
  lengths[,scen] = apply((upper - lower),1,median)
  std_lengths[,scen] = lengths[,scen]/abs(R_true)
  std_lengths[1,scen] = std_lengths[2,scen]
  #matrix of n_scenxlength(c) by 6 containing coverage, std_median_lengths
  #number of bca intervals, nx and ny
  interval_data = rbind(interval_data,cbind(coverage[,scen],std_lengths[,scen],
                                            pnorm(R_true),bounds[[scen]][[5]],
                                            rep(n_x[scen],length(c)),
                                            rep(n_y[scen],length(c))))
}
coverage_data_QMLE_t = data.frame(interval_data,rep("QMLE_t",length(c)))
colnames(coverage_data_QMLE_t) = c("Coverage", "Med_length","R_true","Bca","nx","ny","Estimator")
coverage_data_QMLE_t$nx = factor(coverage_data_QMLE_t$nx)
coverage_data_QMLE_t$ny = factor(coverage_data_QMLE_t$ny)

#---------------------------------
#   QMLE - bootstrap
#---------------------------------
```

```
name = paste("output_",vers,"/estimators/QMLE_raw_output_",factor,".RData",sep="")
load(name)

#Coverage and lengths
coverage = matrix(NA,nrow=length(R_true),ncol=n_scen)
lengths = matrix(NA,nrow=length(R_true),ncol=n_scen)
std_lengths = matrix(NA,nrow=length(R_true),ncol=n_scen)
interval_data = NULL
for (scen in 1:n_scen){
  lower = bounds[[scen]][[1]]
  upper = bounds[[scen]][[2]]
  count = apply((Rmatrix>(lower) & Rmatrix<(upper)),1,sum)
  coverage[,scen] = count/B
  lengths[,scen] = apply((upper - lower),1,median)
  std_lengths[,scen] = lengths[,scen]/abs(R_true)
  std_lengths[1,scen] = std_lengths[2,scen]
  #matrix of n_scenxlength(c) by 6 containing coverage, std_median_lengths
  #number of bca intervals, nx and ny
  interval_data = rbind(interval_data,cbind(coverage[,scen],std_lengths[,scen],
                                            pnorm(R_true),bounds[[scen]][[5]],
                                            rep(n_x[scen],length(c)),
                                            rep(n_y[scen],length(c))))
}
coverage_data_QMLE_boot = data.frame(interval_data,rep("QMLE_boot",length(c)))
colnames(coverage_data_QMLE_boot) = c("Coverage", "Med_length","R_true","Bca","nx","ny","Estimator")
coverage_data_QMLE_boot$nx = factor(coverage_data_QMLE_boot$nx)
coverage_data_QMLE_boot$ny = factor(coverage_data_QMLE_boot$ny)

#---------------------------------
#   Bayes
#---------------------------------

name = paste("output_",vers,"/estimators/Bayes_raw_output_",factor,
             ".RData",sep="")
load(name)

#Coverage and lengths
coverage = matrix(NA,nrow=length(R_true),ncol=n_scen)
lengths = matrix(NA,nrow=length(R_true),ncol=n_scen)
std_lengths = matrix(NA,nrow=length(R_true),ncol=n_scen)
interval_data = NULL
for (scen in 1:n_scen){
  lower = bounds[[scen]][[1]]
  upper = bounds[[scen]][[2]]
  count = apply((Rmatrix>(lower) & Rmatrix<(upper)),1,sum)
  coverage[,scen] = count/B
  lengths[,scen] = apply((upper - lower),1,median)
  std_lengths[,scen] = lengths[,scen]/abs(R_true)
  std_lengths[1,scen] = std_lengths[2,scen]
  #matrix of n_scenxlength(c) by 6 containing coverage, std_median_lengths
  #number of bca intervals, nx and ny
  interval_data = rbind(interval_data,cbind(coverage[,scen],std_lengths[,scen],
                                            pnorm(R_true),rep(NA,length(c)),
                                            rep(n_x[scen],length(c)),
                                            rep(n_y[scen],length(c))))
}
coverage_data_Bayes = data.frame(interval_data,rep("Bayes",length(c)))
colnames(coverage_data_Bayes) = c("Coverage", "Med_length","R_true","Bca","nx","ny","Estimator")
coverage_data_Bayes$nx = factor(coverage_data_Bayes$nx)
coverage_data_Bayes$ny = factor(coverage_data_Bayes$ny)

#---------------------------------
#   NP - Laplace
#---------------------------------
```

```
  name = paste("output_",vers,"/estimators/NP_raw_output_",factor,
               ".RData",sep="")
  load(name)

  #Coverage and lengths
  coverage = matrix(NA,nrow=length(R_true),ncol=n_scen)
  lengths = matrix(NA,nrow=length(R_true),ncol=n_scen)
  std_lengths = matrix(NA,nrow=length(R_true),ncol=n_scen)
  interval_data = NULL
  for (scen in 1:n_scen){
    lower = bounds[[scen]][[1]]
    upper = bounds[[scen]][[2]]
    count = apply((Rmatrix>(lower) & Rmatrix<(upper)),1,sum)
    coverage[,scen] = count/B
    lengths[,scen] = apply((upper - lower),1,median)
    std_lengths[,scen] = lengths[,scen]/abs(R_true)
    std_lengths[1,scen] = std_lengths[2,scen]
    #matrix of n_scenxlength(c) by 6 containing coverage, std_median_lengths
    #number of bca intervals, nx and ny
    interval_data = rbind(interval_data,cbind(coverage[,scen],std_lengths[,scen],
                                              pnorm(R_true),bounds[[scen]][[5]],
                                              rep(n_x[scen],length(c)),
                                              rep(n_y[scen],length(c))))
  }
  coverage_data_NP = data.frame(interval_data,rep("NP",length(c)))
  colnames(coverage_data_NP) = c("Coverage", "Med_length","R_true","Bca","nx","ny","Estimator")
  coverage_data_NP$nx = factor(coverage_data_NP$nx)
  coverage_data_NP$ny = factor(coverage_data_NP$ny)
  coverage_data_NP$Coverage[coverage_data_NP$Coverage<0.5]=0.5

  min(coverage_data_NP$Coverage)

  alldata = rbind(coverage_data_QMLE_boot,coverage_data_QMLE_t,
                  coverage_data_Bayes,coverage_data_NP)
  alldata_12 = subset(alldata,ny==12)

  #-----------------------------------
  # graph
  #-----------------------------------

  #plot for coverages vs med CI length
  g = ggplot(data=alldata) +
    geom_point(aes(Coverage,Med_length,colour=R_true,size=ny),shape=16) +
    geom_point(data=alldata_12,aes(Coverage,Med_length),colour="black",size=5,shape=1) +
    #geom_line(aes(group=ny),colour = "black") +
    geom_vline(xintercept=0.9) +
    geom_hline(yintercept=0, linetype="dashed") +
    theme_bw() +
    #ggtitle("Scatterplot of coverage vs median CI length") +
    ylab("Relative Median Interval Length") + xlab("Coverage") +
    theme(panel.grid.major = element_blank(),
          panel.grid.minor = element_blank(),
          panel.background = element_blank(),
          panel.margin = unit(1,"cm"),
          strip.text.x = element_text(size=18),
          strip.text.y = element_text(size=18),
          axis.title.x = element_text(size=22,vjust=-0.8),
          axis.title.y = element_text(size=22,vjust=0.3),
          axis.text.x = element_text(size=18),
          axis.text.y = element_text(size=18),
          plot.title = element_text(size=22,vjust=2),
          legend.title = element_text(size=20),
          legend.text = element_text(size=18),
          legend.position = "bottom",
          legend.key.width = unit(1,"line"),
          legend.key = element_rect(linetype=0))  +
```

```
  guides(color = guide_colourbar(barwidth=25)) +
  scale_colour_gradientn(name="True R: ",colours=rainbow(4),
                         breaks=c(0,0.1,0.2,0.3,0.4,0.5)) +
  scale_size_manual(name="Effect sample size: ",
                    breaks = c(2,5,12,20,100),
                    values = c(3,4,5,6,7),
                    labels = c("2   ","5   ","12   ","20   ","100")) +
  scale_y_continuous(breaks = seq(0,12,3),
                     expand=c(0,0),limits=c(-1,12)) +
  scale_x_continuous(breaks=seq(0.5,1,by=0.1),expand=c(0,0),limits=c(0.5,1)) +
  facet_grid(Estimator~nx,labeller=mf_labeller)

name = paste("coverages_lengths_2s_",factor,".png",sep="")
ggsave(file=name,plot = g,width=10,height=18)
ggsave(file="test_noncent2.png",plot = g,width=10,height=18)
```

1.3.2 Sampling distribution of estimators

```
#version of code
vers = "v9"
#sigma case
factor = 5

n_x = rep(c(5,100),each=5)
n_y = rep(c(2,5,12,20,100),2)
mu_x = -2.948
mu_y = -2.948
sig2_x = 0.0738
sig2_y = (factor*sqrt(sig2_x))^2

n_scen = length(n_x)

pnorm1 <- function(x){
  #original
  #newR = pnorm(x)
  #probit
  newR = x
  newR
}

source("functions/functions.R")

if (factor == 1)
  #for equal variances
  c = seq(0,2.9,0.1) else #mean difference producing range of R values
    if (factor == 5)
      #for 5x
      c = seq(0,10.4,0.4) else #mean difference producing range of R values
        if (factor == 0.2)
          #for 0.2x
          c = seq(0,2.1,0.1) #mean difference producing range of R values

R_true = pnorm1((mu_x - mu_y - c)/sqrt(sig2_x + sig2_y))

name = paste("output_",vers,"/estimators/MLE_raw_output_",factor,
             ".RData",sep="")
load(name)
MLE_bounds = bounds
name = paste("output_",vers,"/estimators/QMLE_raw_output_",factor,
             ".RData",sep="")
load(name)
QMLE_bounds = bounds
name = paste("output_",vers,"/estimators/Bayes_raw_output_",factor,
             ".RData",sep="")
load(name)
Bayes_bounds = bounds
```

```
name = paste("output_",vers,"/estimators/NP_raw_output_",factor,
             ".RData",sep="")
load(name)
NP_bounds = bounds
name = paste("output_",vers,"/estimators/NP_raw_output_inf_",factor,
             ".RData",sep="")
load(name)
NPi_bounds = bounds
#name = paste("output_",vers,"/estimators/NP_raw_output_ss_",factor,
#             ".RData",sep="")
#load(name)
#NPs_bounds = bounds

plots = list()

for (scen in 1:n_scen){
  R_MLE = (MLE_bounds[[scen]][[4]])
  R_QMLE = (QMLE_bounds[[scen]][[4]])
  R_Bayes = (Bayes_bounds[[scen]][[4]])
  R_NP = (NP_bounds[[scen]][[4]])
  R_NPi = (NPi_bounds[[scen]][[4]])
  R_NPs = (NPs_bounds[[scen]][[4]])
  l_MLE = apply(R_MLE,1,quantile,probs=0.025)
  l_QMLE = apply(R_QMLE,1,quantile,probs=0.025)
  l_Bayes = apply(R_Bayes,1,quantile,probs=0.025)
  l_NP = apply(R_NP,1,quantile,probs=0.025)
  l_NPi = apply(R_NPi,1,quantile,probs=0.025)
  l_NPs = apply(R_NPs,1,quantile,probs=0.025)
  u_MLE = apply(R_MLE,1,quantile,probs=0.975)
  u_QMLE = apply(R_QMLE,1,quantile,probs=0.975)
  u_Bayes = apply(R_Bayes,1,quantile,probs=0.975)
  u_NP = apply(R_NP,1,quantile,probs=0.975)
  u_NPi = apply(R_NP,1,quantile,probs=0.975)
  u_NPs = apply(R_NP,1,quantile,probs=0.975)
  m_MLE = apply(R_MLE,1,quantile,probs=0.5)
  m_QMLE = apply(R_QMLE,1,quantile,probs=0.5)
  m_Bayes = apply(R_Bayes,1,quantile,probs=0.5)
  m_NP = apply(R_NP,1,quantile,probs=0.5)
  m_NPi = apply(R_NP,1,quantile,probs=0.5)
  m_NPs = apply(R_NP,1,quantile,probs=0.5)

  min_R = qnorm(rep(1/(n_x[scen]*n_y[scen]+1),length(c)))

  data_MLE = data.frame(R=R_true,lb = min_R,lower=l_MLE,med=m_MLE,upper=u_MLE,
                        est=rep("MLE",length(c)))
  data_QMLE = data.frame(R=R_true,lb = min_R,lower=l_QMLE,med=m_QMLE,upper=u_QMLE,
                         est=rep("QMLE",length(c)))
  data_Bayes = data.frame(R=R_true,lb = min_R,lower=l_Bayes,med=m_Bayes,upper=u_Bayes,
                          est=rep("Bayes",length(c)))
  data_NP = data.frame(R=R_true,lb = min_R,lower=l_NP,med=m_NP,upper=u_NP,
                       est=rep("NP",length(c)))

  totaldata = rbind(data_MLE,data_QMLE,data_Bayes,data_NP)

  plot = ggplot(data=totaldata,aes(x=R,y=lower)) +
    geom_line(data=totaldata,aes(x=R,y=lower,colour=est,linetype=est),size=3) +
    #geom_line(data=totaldata,aes(x=R,y=med,colour=est,linetype=est),size=3) +
    geom_line(data=totaldata,aes(x=R,y=upper,colour=est,linetype=est),size=3) +
    geom_line(data=totaldata,aes(x=R,y=lb),size=1,colour="grey") +
    geom_abline(intercept = 0, slope = 1,linetype = "dotted") +
    theme_bw() +
    #annotate(geom = "text",label="(D)",x=2.75,y=0.5,size=7) +
    ggtitle(paste("nx=",n_x[scen]," ny=",n_y[scen],sep="")) +
    ylab(expression(probit(hat(R)))) + xlab(expression(probit(R))) +
    theme(panel.grid.major = element_blank(),
          panel.grid.minor = element_blank(),
```

```
          panel.background = element_blank(),
          axis.title.x = element_text(size=22),
          axis.title.y = element_text(size=22),
          axis.text.x = element_text(size=18),
          axis.text.y = element_text(size=18),
          plot.title = element_text(size=22,vjust=2),
          legend.title = element_text(size=22),
          legend.text = element_text(size=18),
          legend.position = "bottom",
          legend.key = element_rect(linetype=0),
          legend.key.width = unit(3,"cm"),
          plot.margin = unit(c(10,10,10,10),"mm"))  +
    scale_y_continuous(breaks = c(-13,-11,-9,-7,-5,-3,-1,1),
                       expand=c(0,0),limits=c(-13,1.2)) +
    #scale_y_continuous(breaks = c(1e-14,1e-12,1e-10,1e-8,1e-6,1e-4,1e-2,1),
    #                   expand=c(0,0),
    #                   trans=log2_trans(),
    #                   labels = trans_format("log10", math_format(10^.x)),
    #                   limits=c(1e-14,1)) +
    scale_x_continuous(breaks = c(-7,-5,-3,-1,1),
                       expand=c(0,0),limits=c(-8,1.2)) +
    scale_linetype_manual(name="Estimator ",
                          values=c("solid","longdash","dashed","dotdash"),
                          labels=c("MLE ","QMLE ","Bayes ","NP ")) +
    scale_colour_manual(name="Estimator ",
                        values=c("#E69F00", "#009E73" , "#CC79A7","#56B4E9"),
                        labels=c("MLE ","QMLE ","Bayes ","NP "))

  plots[[scen]] <- plot
}
legend = g_legend_f(plot)
figure = arrangeGrob(arrangeGrob(plots[[1]]+theme(legend.position="none"),
                                 plots[[6]]+theme(legend.position="none"),
                                 plots[[2]]+theme(legend.position="none"),
                                 plots[[7]]+theme(legend.position="none"),
                                 plots[[3]]+theme(legend.position="none"),
                                 plots[[8]]+theme(legend.position="none"),
                                 plots[[4]]+theme(legend.position="none"),
                                 plots[[9]]+theme(legend.position="none"),
                                 plots[[5]]+theme(legend.position="none"),
                                 plots[[10]]+theme(legend.position="none"),
                                 nrow=5),
                     #main=textGrob("2.5% and 97.5% percentiles of sampling distribution",
                     gp=gpar(fontsize=26))),
                     legend, nrow=2,heights=c(27,1))
#name = paste("sampling_distribution_R_median_",factor,".png",sep="")
name = paste("sampling_distribution_R_percentiles_",factor,".png",sep="")
ggsave(file=name,plot = figure,width=14,height=28)
```

1.3.3 Bayes posterior summary statistics

```
setwd("D:/PhD/Thesis/Paper_2/R_Files")

library(coda) #for HPD intervals
library(ggplot2)
library(gridExtra) #for legend specifications
library(scales) #log scale in ggplot
library(modeest) #to calculate mode

#----------------------------------------------
# file name components
#----------------------------------------------
#version of code
vers = "v9"
#sigma case
factor = 5
```

```
#Parameter choices
n_x = rep(c(5,100),each=5)
n_y = rep(c(2,5,12,20,100),2)
mu_x = -2.948
mu_y = -2.948
sig2_x = 0.0738
sig2_y = (factor*sqrt(sig2_x))^2

n_scen = length(n_x)

source("functions/functions.R")
pnorm1 <- function(x){
  #original
  #newR = pnorm(x)
  #probit
  newR = x
  newR
}

if (factor == 1)
  #for equal variances
  c = seq(0,2.9,0.1) else #mean difference producing range of R values
    if (factor == 5)
      #for 5x
      c = seq(0,10.4,0.4) else #mean difference producing range of R values
        if (factor == 0.2)
          #for 0.2x
          c = seq(0,2.1,0.1) #mean difference producing range of R values

R_true = pnorm1((mu_x - mu_y - c)/sqrt(sig2_x + sig2_y))

#Only scenario 8
scen=8
#Original

R_true = pnorm((mu_x - mu_y - c)/sqrt(sig2_x + sig2_y))

#Study difference in posterior summary statistics
plots = list()

name = paste("output_",vers,"/estimators/Bayes_raw_output_orig",factor,
             ".Rdata",sep="")
load(name)

R_mean = bounds[[scen]][[6]]
R_median = bounds[[scen]][[4]]
R_mode = bounds[[scen]][[5]]
l_mean = apply(R_mean,1,quantile,probs=0.025)
u_mean = apply(R_mean,1,quantile,probs=0.975)
m1_mean = apply(R_mean,1,quantile,probs=0.5)
l_median = apply(R_median,1,quantile,probs=0.025)
u_median = apply(R_median,1,quantile,probs=0.975)
m1_median = apply(R_median,1,quantile,probs=0.5)
l_mode = apply(R_mode,1,quantile,probs=0.025)
u_mode = apply(R_mode,1,quantile,probs=0.975)
m1_mode = apply(R_mode,1,quantile,probs=0.5)

data_mean = data.frame(R=R_true,lower=l_mean,med=m1_mean,upper=u_mean,
est=rep("Mean   ",length(c)))
data_median = data.frame(R=R_true,lower=l_median,med=m1_median,upper=u_median,
est=rep("Median   ",length(c)))
data_mode = data.frame(R=R_true,lower=l_mode,med=m1_mode,upper=u_mode,
est=rep("Mode   ",length(c)))

totaldata = rbind(data_median,data_mean,data_mode)
```

```
  plot1 = ggplot(data=totaldata,aes(x=R,y=lower,group=est,colour=est,linetype=est)) +
    #geom_line(data=totaldata,aes(x=R,y=lower),size=1.5) +
    geom_line(data=totaldata,aes(x=R,y=med,group=est),size=3) +
    #geom_line(data=totaldata,aes(x=R,y=upper),size=1.5) +
    geom_abline(intercept = 0, slope = 1,linetype="dotted") +
    theme_bw() +
    annotate(geom = "text",label="(A)",x=1e-13,y=0.2,size=7) +
    ylab(expression(hat(R))) + xlab(expression(R)) +
    theme(panel.grid.major = element_blank(),
          panel.grid.minor = element_blank(),
          panel.background = element_blank(),
          axis.title.x = element_text(size=22),
          axis.title.y = element_text(size=22),
          axis.text.x = element_text(size=18),
          axis.text.y = element_text(size=18),
          plot.title = element_text(size=22,vjust=2),
          legend.title = element_text(size=22),
          legend.text = element_text(size=18),
          legend.position = "bottom",
          legend.key = element_rect(linetype=0),
          legend.key.width = unit(3,"cm"),
          plot.margin = unit(c(10,10,10,10),"mm"))  +
    scale_y_continuous(breaks = c(1e-14,1e-12,1e-10,1e-8,1e-6,1e-4,1e-2,1),
                       expand=c(0,0),
                       trans=log2_trans(),
                       labels = trans_format("log10", math_format(10^.x)),
                       limits=c(1e-14,1)) +
    scale_x_continuous(breaks = c(1e-14,1e-12,1e-10,1e-8,1e-6,1e-4,1e-2,1),
                       expand=c(0,0),
                       trans=log2_trans(),
                       labels = trans_format("log10", math_format(10^.x)),
                       limits=c(1e-14,1)) +
    scale_linetype_manual(name="Estimator ",
                          values=c("solid","dotdash","dashed")) +
    scale_colour_manual(name="Estimator ",values=c("#E69F00", "#CC79A7", "#56B4E9"))


  plot2 = ggplot(data=totaldata,aes(x=R,y=lower,group=est,colour=est,linetype=est)) +
    geom_line(data=totaldata,aes(x=R,y=lower),size=3) +
    #geom_line(data=totaldata,aes(x=R,y=med,group=est),size=2) +
    geom_line(data=totaldata,aes(x=R,y=upper),size=3) +
    geom_abline(intercept = 0, slope = 1,linetype="dotted") +
    theme_bw() +
    annotate(geom = "text",label="(B)",x=1e-13,y=0.2,size=7) +
    ylab(expression(hat(R))) + xlab(expression(R)) +
    theme(panel.grid.major = element_blank(),
          panel.grid.minor = element_blank(),
          panel.background = element_blank(),
          axis.title.x = element_text(size=22),
          axis.title.y = element_text(size=22),
          axis.text.x = element_text(size=18),
          axis.text.y = element_text(size=18),
          plot.title = element_text(size=22,vjust=2),
          legend.title = element_text(size=22),
          legend.text = element_text(size=18),
          legend.position = "bottom",
          legend.key = element_rect(linetype=0),
          legend.key.width = unit(3,"cm"),
          plot.margin = unit(c(10,10,10,10),"mm"))  +
    scale_y_continuous(breaks = c(1e-14,1e-12,1e-10,1e-8,1e-6,1e-4,1e-2,1),
                       expand=c(0,0),
                       trans=log2_trans(),
                       labels = trans_format("log10", math_format(10^.x)),
                       limits=c(1e-14,1)) +
    scale_x_continuous(breaks = c(1e-14,1e-12,1e-10,1e-8,1e-6,1e-4,1e-2,1),
```

```
                       expand=c(0,0),
                       trans=log2_trans(),
                       labels = trans_format("log10", math_format(10^.x)),
                       limits=c(1e-14,1)) +
    scale_linetype_manual(name="Estimator ",
                          values=c("solid","dotdash","dashed")) +
    scale_colour_manual(name="Estimator ",values=c("#E69F00", "#CC79A7", "#56B4E9"))


  #probit
  R_true = pnorm1((mu_x - mu_y - c)/sqrt(sig2_x + sig2_y))
  name = paste("output_",vers,"/estimators/Bayes_raw_output_",factor,
               ".Rdata",sep="")
  load(name)

  R_mean = bounds[[scen]][[6]]
  R_median = bounds[[scen]][[4]]
  R_mode = bounds[[scen]][[5]]
  l_mean = apply(R_mean,1,quantile,probs=0.025)
  u_mean = apply(R_mean,1,quantile,probs=0.975)
  m1_mean = apply(R_mean,1,quantile,probs=0.5)
  l_median = apply(R_median,1,quantile,probs=0.025)
  u_median = apply(R_median,1,quantile,probs=0.975)
  m1_median = apply(R_median,1,quantile,probs=0.5)
  l_mode = apply(R_mode,1,quantile,probs=0.025)
  u_mode = apply(R_mode,1,quantile,probs=0.975)
  m1_mode = apply(R_mode,1,quantile,probs=0.5)

  data_mean = data.frame(R=R_true,lower=l_mean,med=m1_mean,upper=u_mean,
  est=rep("Mean   ",length(c)))
  data_median = data.frame(R=R_true,lower=l_median,med=m1_median,upper=u_median,
  est=rep("Median   ",length(c)))
  data_mode = data.frame(R=R_true,lower=l_mode,med=m1_mode,upper=u_mode,
  est=rep("Mode   ",length(c)))

  totaldata = rbind(data_median,data_mean,data_mode)

  plot3 = ggplot(data=totaldata,aes(x=R,y=lower,group=est,colour=est,linetype=est)) +
    #geom_line(data=totaldata,aes(x=R,y=lower),size=1.5) +
    geom_line(data=totaldata,aes(x=R,y=med,group=est),size=3) +
    #geom_line(data=totaldata,aes(x=R,y=upper),size=1.5) +
    geom_abline(intercept = 0, slope = 1,linetype="dotted") +
    theme_bw() +
    annotate(geom = "text",label="(C)",x=-8.2,y=0.2,size=7) +
    ylab(expression(probit(hat(R)))) + xlab(expression(probit(R))) +
    theme(panel.grid.major = element_blank(),
          panel.grid.minor = element_blank(),
          panel.background = element_blank(),
          axis.title.x = element_text(size=22),
          axis.title.y = element_text(size=22),
          axis.text.x = element_text(size=18),
          axis.text.y = element_text(size=18),
          plot.title = element_text(size=22,vjust=2),
          legend.title = element_text(size=22),
          legend.text = element_text(size=18),
          legend.position = "bottom",
          legend.key = element_rect(linetype=0),
          legend.key.width = unit(3,"cm"),
          plot.margin = unit(c(10,10,10,10),"mm"))  +
    scale_y_continuous(breaks = c(-11,-9,-7,-5,-3,-1,1),
                       expand=c(0,0),limits=c(-13,1)) +
    scale_x_continuous(breaks = c(-9,-7,-5,-3,-1,1),
                       expand=c(0,0),limits=c(-9,1)) +
    scale_linetype_manual(name="Estimator ",
                          values=c("solid","dotdash","dashed")) +
    scale_colour_manual(name="Estimator ",values=c("#E69F00", "#CC79A7", "#56B4E9"))
```

```
plot4 = ggplot(data=totaldata,aes(x=R,y=lower,group=est,colour=est,linetype=est)) +
  geom_line(data=totaldata,aes(x=R,y=lower),size=3) +
  #geom_line(data=totaldata,aes(x=R,y=med,group=est),size=2) +
  geom_line(data=totaldata,aes(x=R,y=upper),size=3) +
  geom_abline(intercept = 0, slope = 1,linetype="dotted") +
  theme_bw() +
  annotate(geom = "text",label="(D)",x=-8.2,y=0.2,size=7) +
  ylab(expression(probit(hat(R)))) + xlab(expression(probit(R))) +
  theme(panel.grid.major = element_blank(),
        panel.grid.minor = element_blank(),
        panel.background = element_blank(),
        axis.title.x = element_text(size=22),
        axis.title.y = element_text(size=22),
        axis.text.x = element_text(size=18),
        axis.text.y = element_text(size=18),
        plot.title = element_text(size=22,vjust=2),
        legend.title = element_text(size=22),
        legend.text = element_text(size=18),
        legend.position = "bottom",
        legend.key = element_rect(linetype=0),
        legend.key.width = unit(3,"cm"),
        plot.margin = unit(c(10,10,10,10),"mm"))  +
  scale_y_continuous(breaks = c(-11,-9,-7,-5,-3,-1,1),
                     expand=c(0,0),limits=c(-13,1)) +
  scale_x_continuous(breaks = c(-9,-7,-5,-3,-1,1),
                     expand=c(0,0),limits=c(-9,1)) +
  scale_linetype_manual(name="Estimator ",
                        values=c("solid","dotdash","dashed")) +
  scale_colour_manual(name="Estimator ",values=c("#E69F00", "#CC79A7", "#56B4E9"))

legend = g_legend_f(plot1)
figure_sub = arrangeGrob(arrangeGrob(plot1+theme(legend.position="none"),
                                     plot2+theme(legend.position="none"),
                                     plot3+theme(legend.position="none"),
                                     plot4+theme(legend.position="none"),
                                     nrow=2),
                         legend, nrow=2,heights=c(14,1))
ggsave(file="Bayes_posterior_summary_sub.png",plot = figure_sub,width=14,height=15)
```

2 Figures and Tables

2.1 Application

In this section, we provide the extra tables and figures relating to the case study.

Table S1: Test statistic values and p-values for six normality tests on the nano-Ag exposure and effect concentration data of Gottschalk et al. (2013).

	Exposure	Effect
Normality Test	Statistic (p-value)	Statistic (p-value)
Anderson-Darling	0.6724 (0.0789)	0.3761 (0.3519)
Cramer-von Mises	0.1219 (0.0564)	0.0535 (0.4301)
Kolmogorov-Smirnov	0.0285 (0.0538)	0.1520 (0.6214)
Pearson chi-square	35.1360 (0.2001)	4.0000 (0.2615)
Shapiro-Francia	0.9973 (0.0878)	0.9183 (0.2293)
Shapiro-Wilk	0.9975 (0.1238)	0.9291 (0.3710)

Figure S1: Histograms of the ratio of the 95% upper bound to the upper bound of the 90% credible interval of the Bayesian estimator for different values of the true R and sample sizes $n_x = 100$ and $n_y = 12$. The vertical red line indicates the corresponding ratio in the case study.

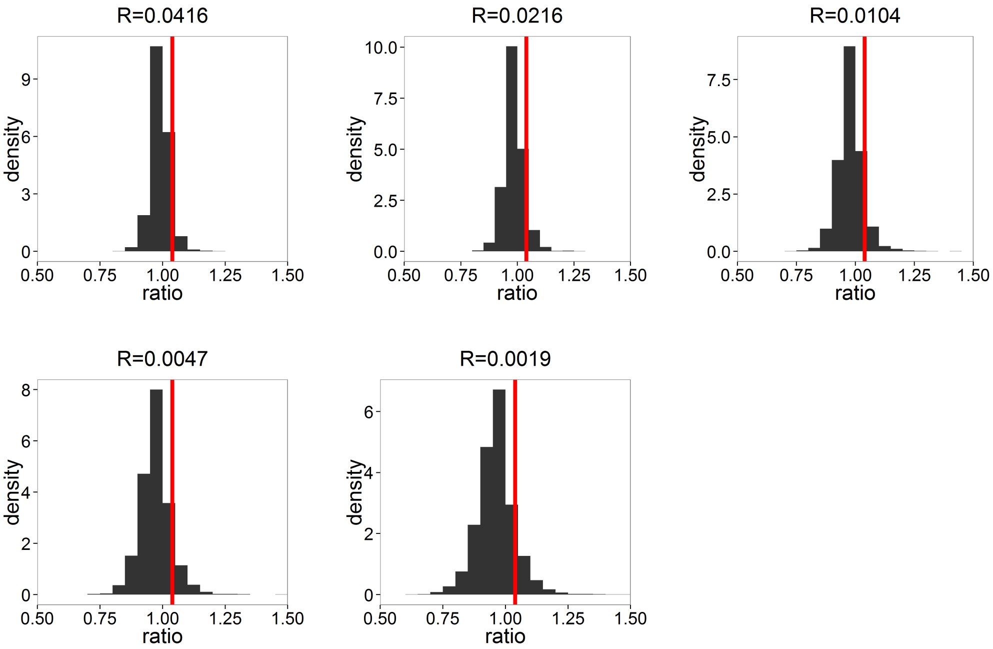

Figure S2: Histograms of the distances between the 95% upper bound and the true R value of the QMLE (left column) and Bayesian estimator (right column) for different values of the true R and sample sizes $n_x = 100$ and $n_y = 12$. The vertical red line indicates the corresponding upper bounds in the case study.

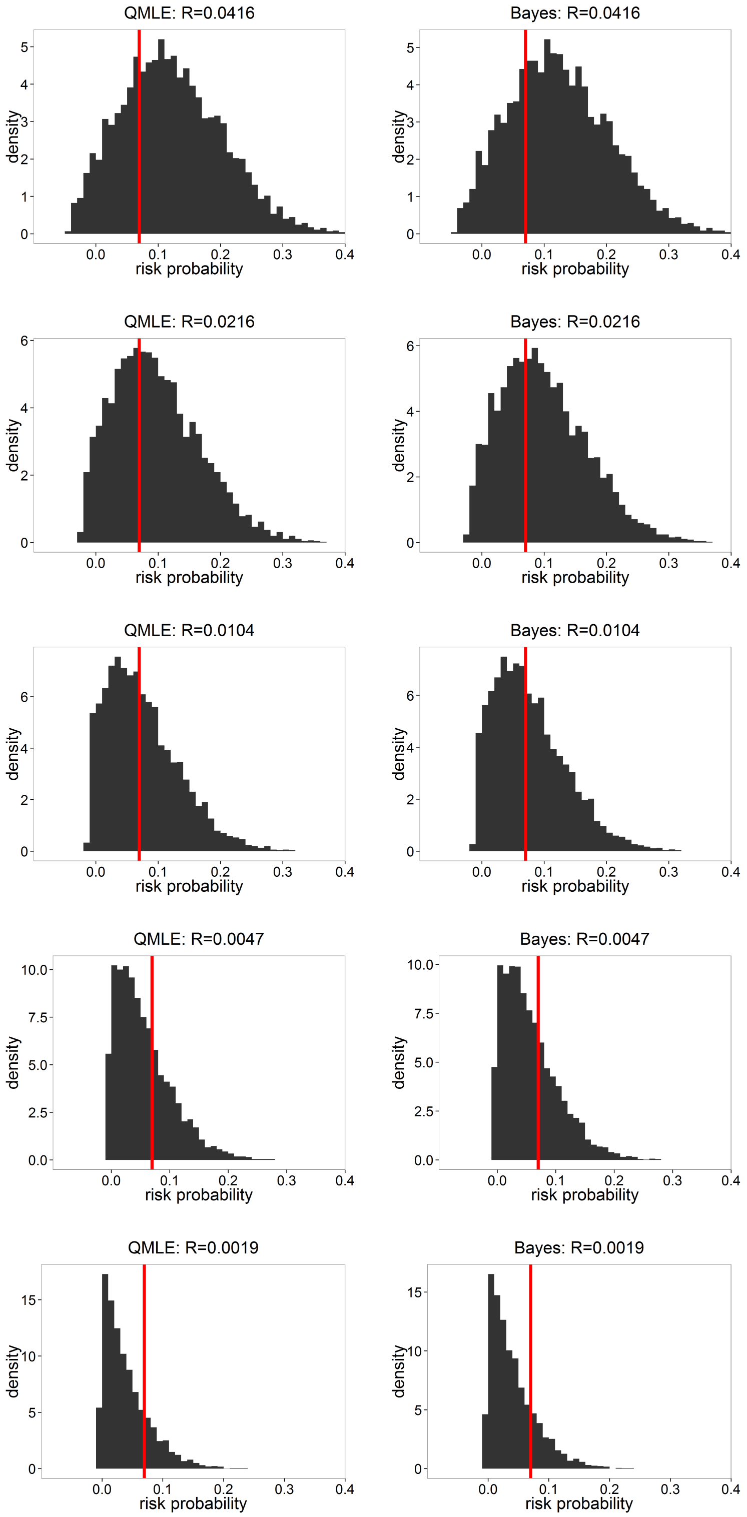

Figure S3: Histograms of the ratio of the 95% upper bound of the Bayesian estimator to QMLE for different values of the true R and sample sizes $n_x = 100$ and $n_y = 12$. The vertical red line indicates the corresponding ratio in the case study.

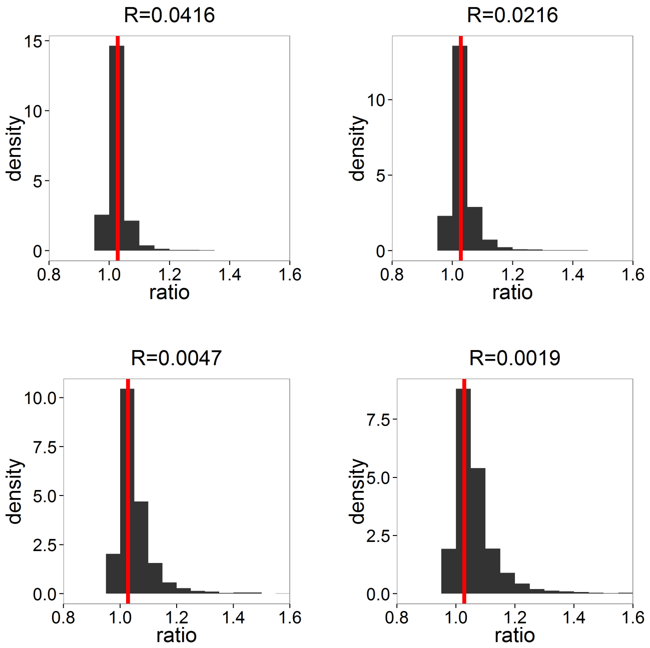

2.2 Case: $\sigma_y = \sigma_x$

Figure S4: The median of the sampling distribution of the three Bayesian point estimators (mean, median and mode) calculated on the original scale and plotted on log_{10}-scale. When plotted on log_{10}-scale, a zero mode becomes $-\infty$. The diagonal dotted line represents the values where $\hat{R} = R$.

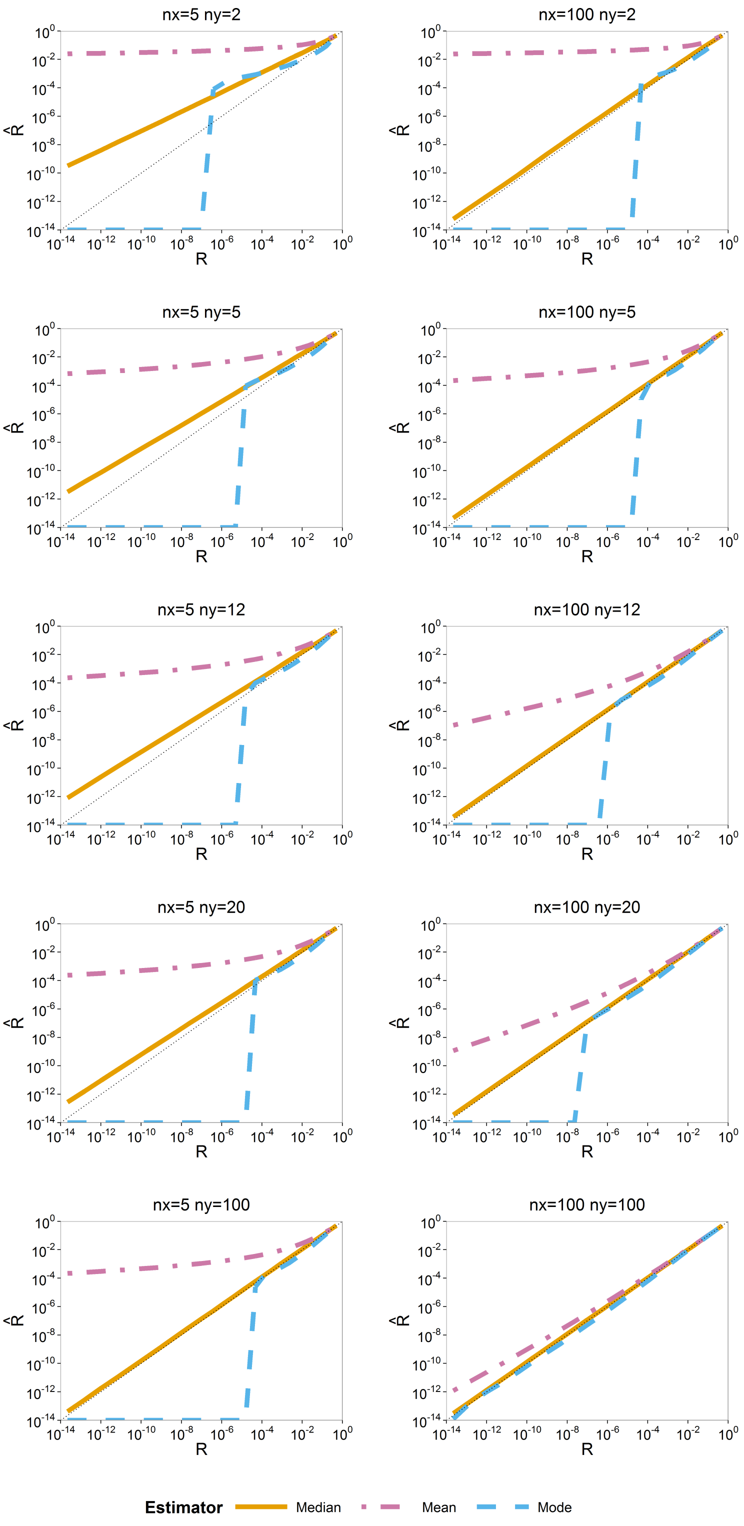

Figure S5: The 0.025 and 0.975 quantiles of the sampling distribution of the three Bayesian point estimators (mean, median and mode) calculated on the original scale and plotted on log_{10}-scale. When plotted on log_{10}-scale, a zero mode becomes $-\infty$. The diagonal dotted line represents the values where $\hat{R} = R$.

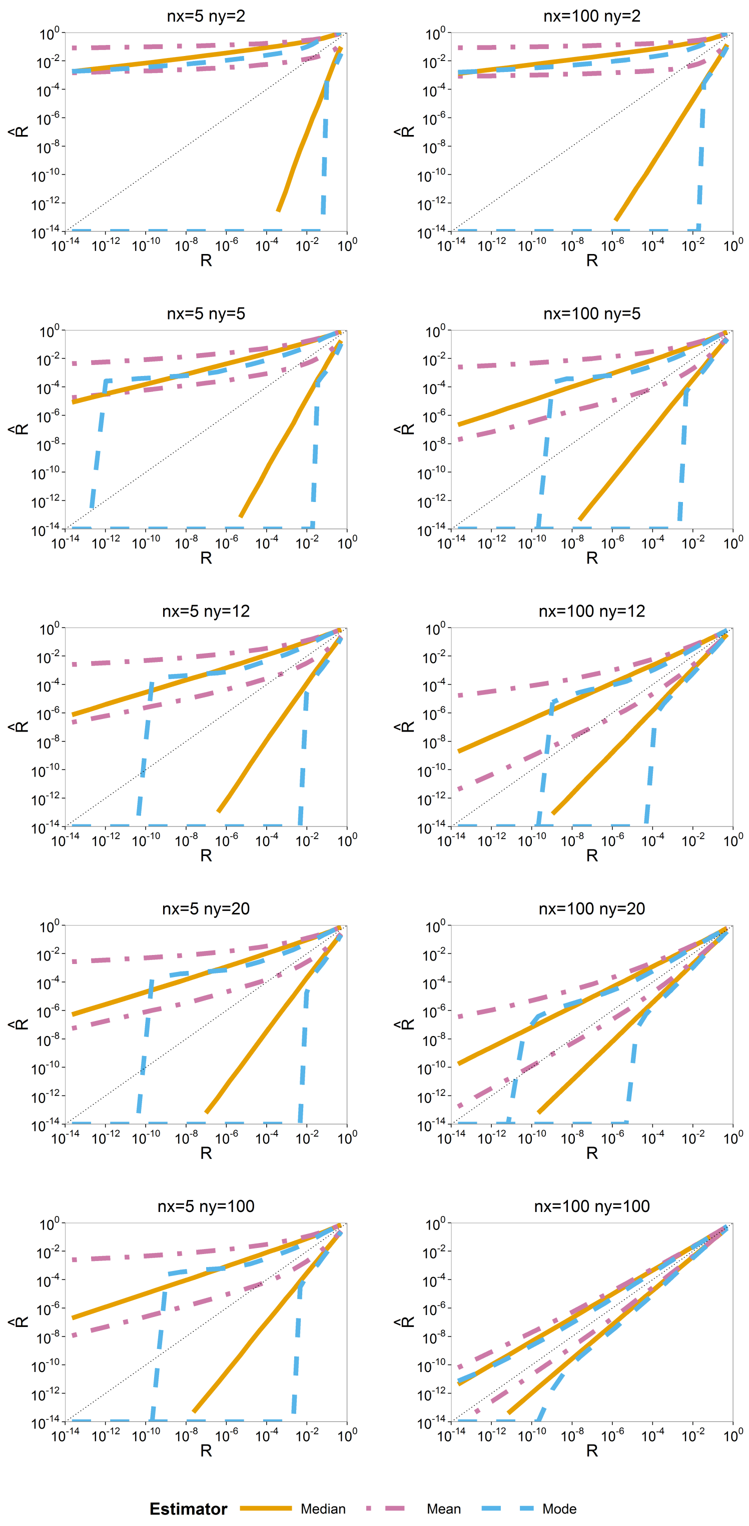

Figure S6: The median of the sampling distribution of the three Bayesian point estimators (mean, median and mode) calculated on the probit scale. The diagonal dotted line represents the values where $probit(\hat{R}) = probit(R)$.

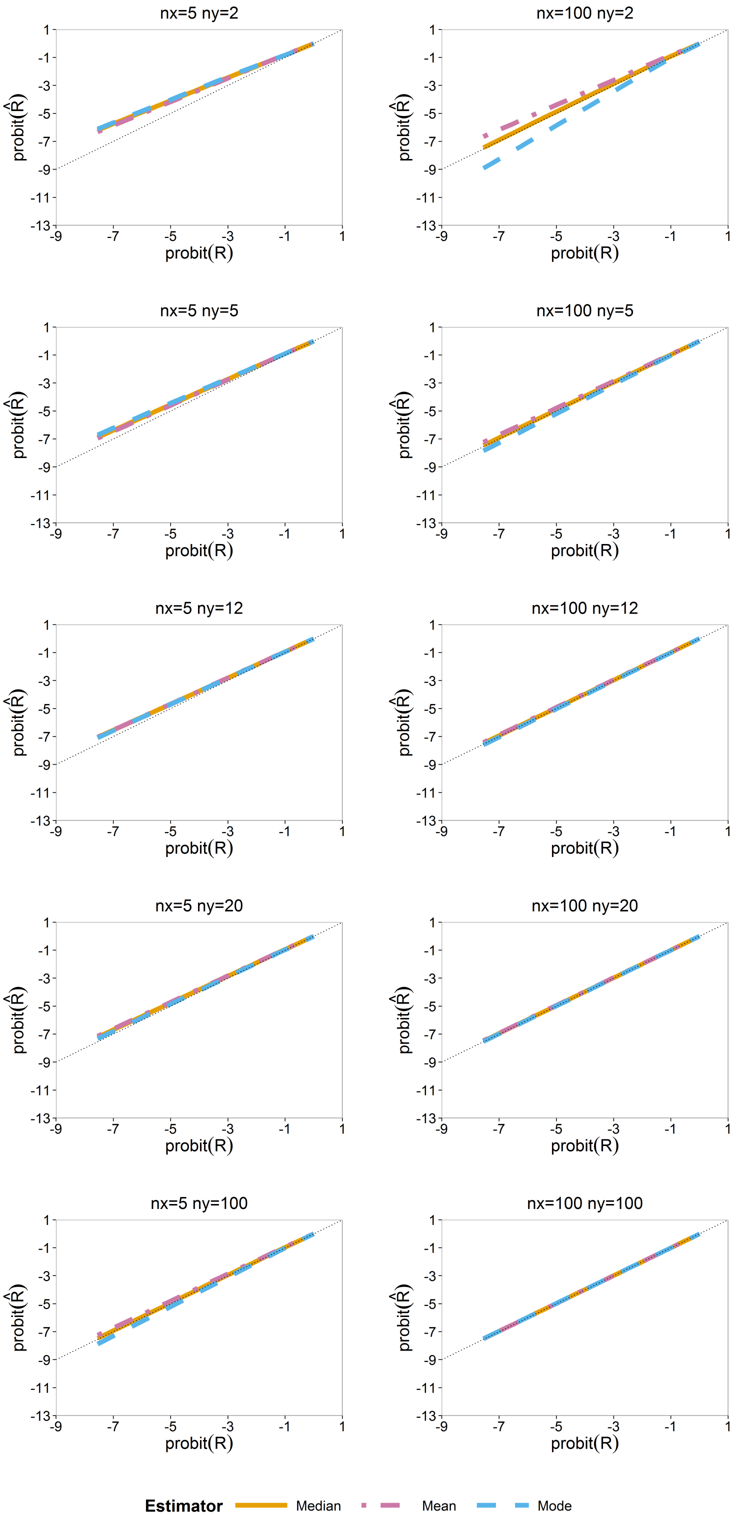

Figure S7: The 0.025 and 0.975 quantiles of the sampling distribution of the three Bayesian point estimators (mean, median and mode) calculated on the probit scale. The diagonal dotted line represents the values where $probit(\hat{R}) = probit(R)$.

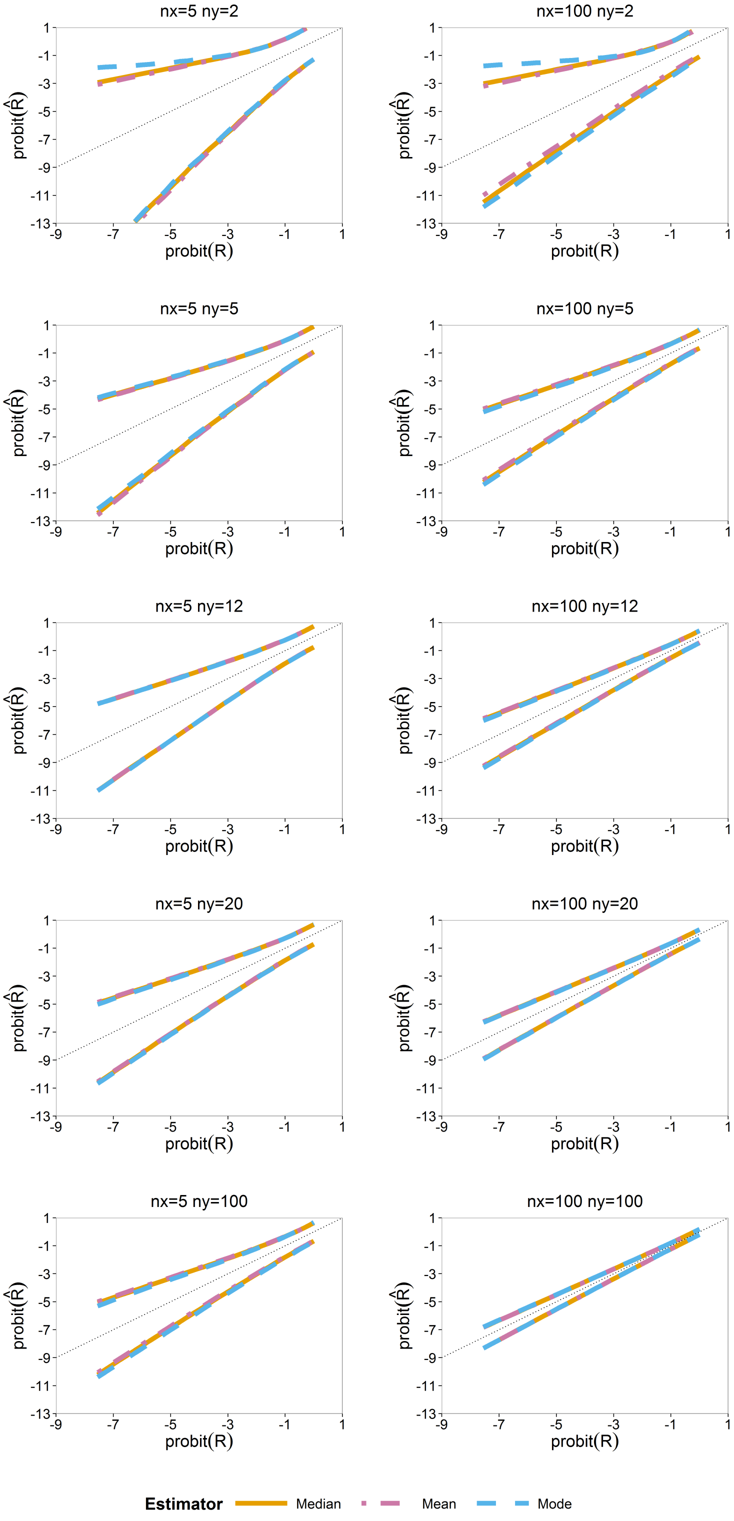

Figure S8: The median of the sampling distribution of the four point estimators, $\hat{R}_{MLE}$, $\hat{R}_{QMLE}$, $\hat{R}_{Bayes}$ and $\hat{R}_{NP}$ calculated on the probit scale. The diagonal dotted line represents the values where $probit(\hat{R}) = probit(R)$. The horizontal grey line gives the lower bound ($probit\left(\frac{1}{n_x n_y + 2}\right)$) of $probit(\hat{R}_{NP})$.

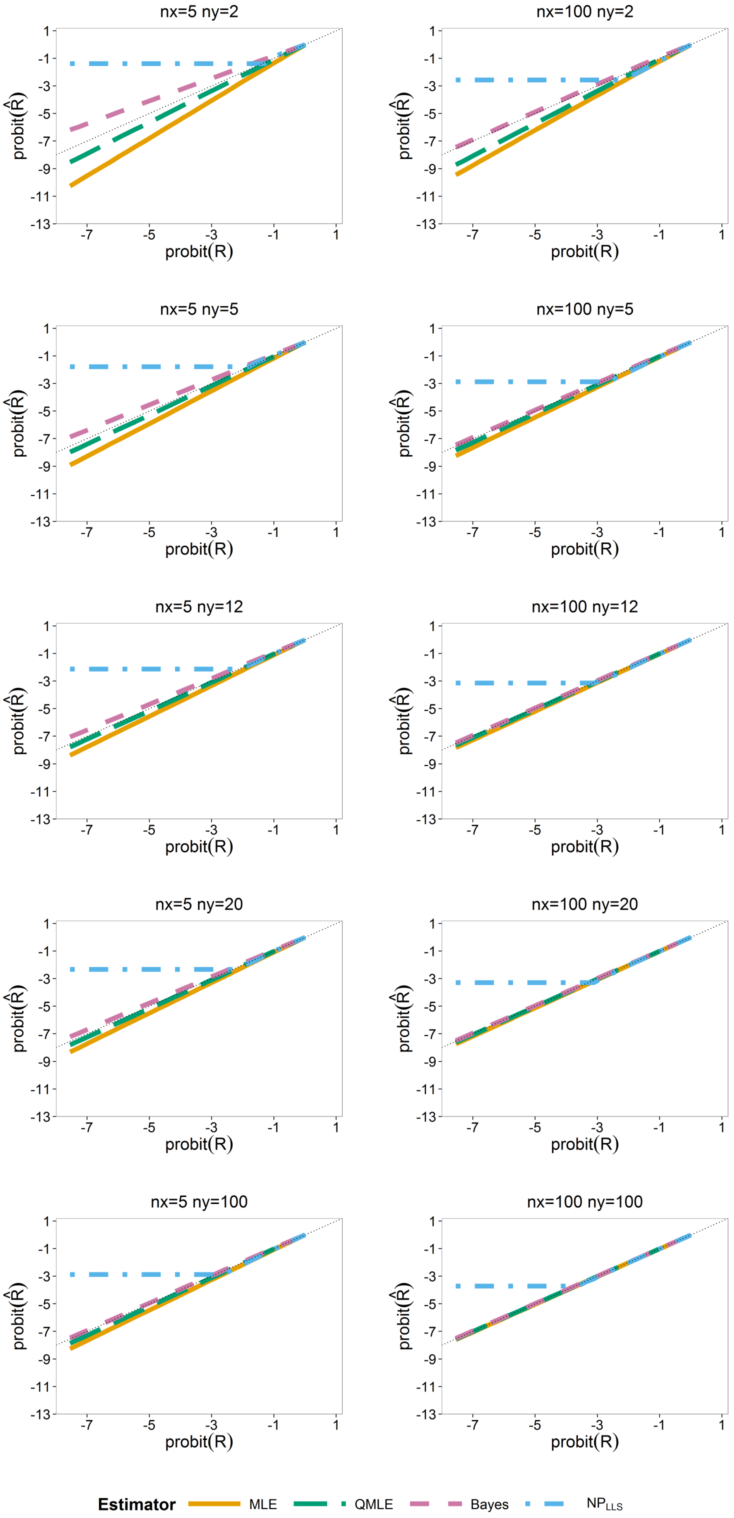

Figure S9: The 0.025 and 0.975 quantiles of the sampling distribution of the four point estimators, $\hat{R}_{MLE}$, $\hat{R}_{QMLE}$, $\hat{R}_{Bayes}$ and $\hat{R}_{NP}$ calculated on the probit scale. The diagonal dotted line represents the values where $probit(\hat{R}) = probit(R)$. The horizontal grey line gives the lower bound ($probit\left(\frac{1}{n_x n_y+2}\right)$) of $probit(\hat{R}_{NP})$.

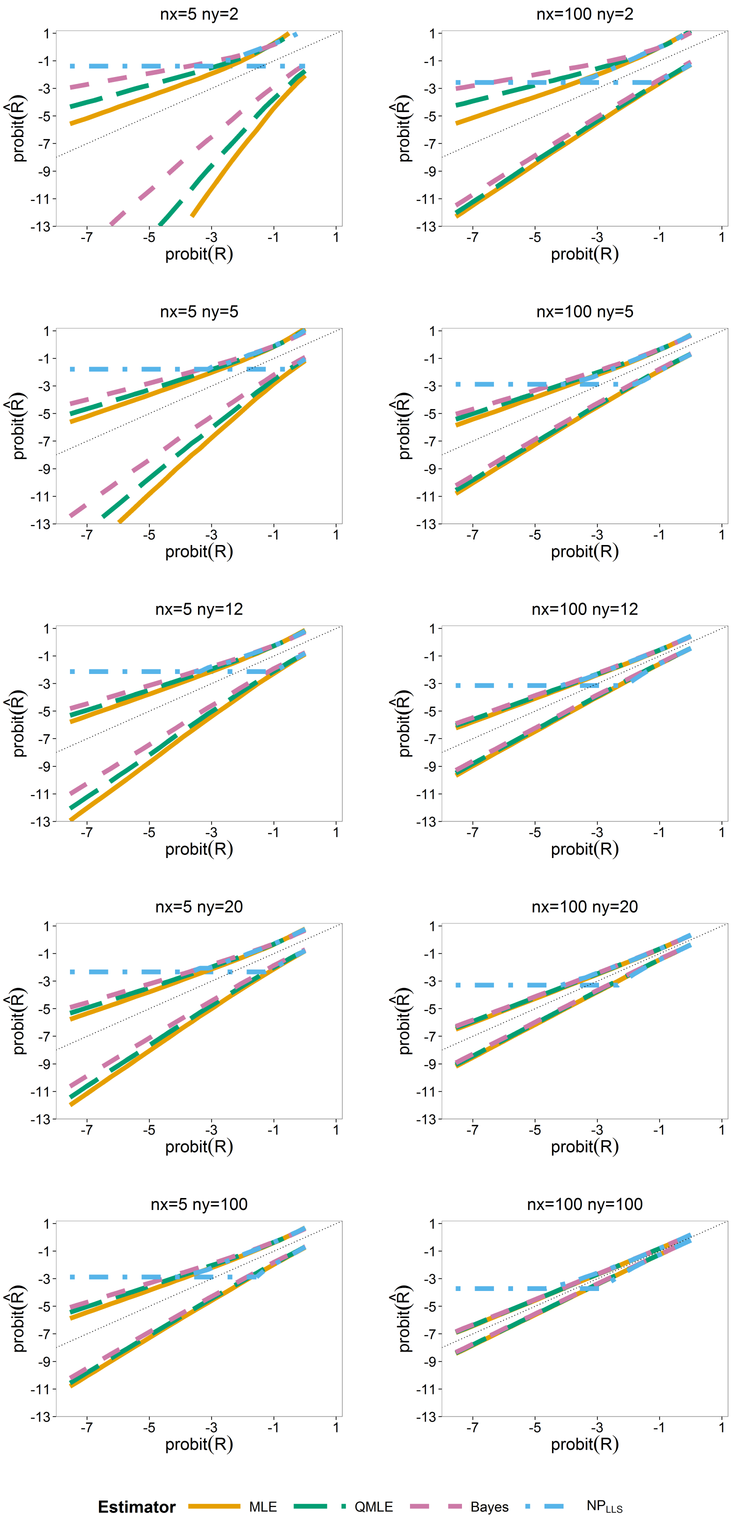

Figure S10: Scatterplots of the 90% two-sided coverage probabilities against the relative median interval length calculated on the probit scale. The value of the true R value is illustrated by the color scale. The size of the dots corresponds to the size of the sample size of effect. A vertical reference line is drawn at a coverage probability of 90%. The points corresponding to $n_y = 12$ are indicated by an open black circle.

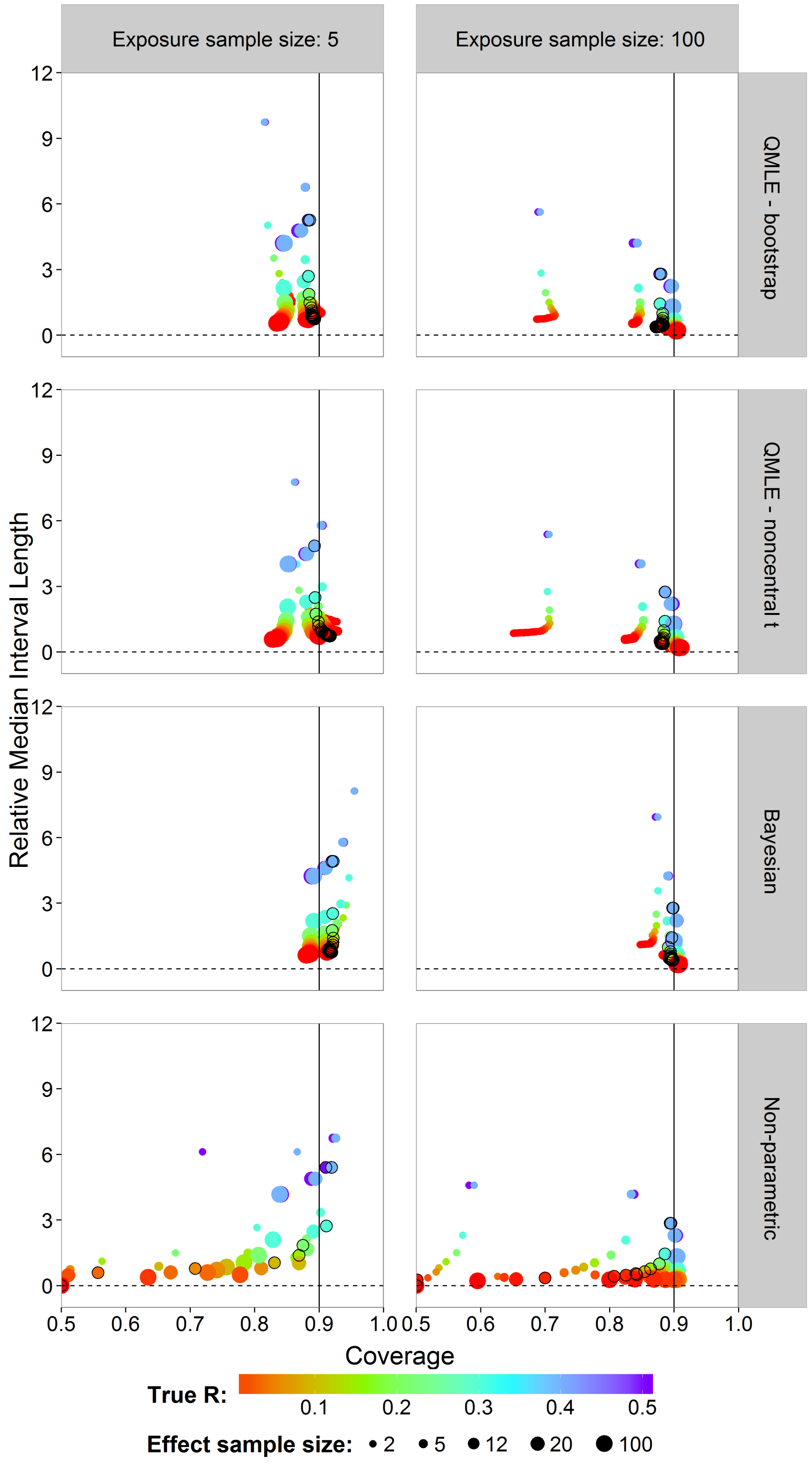

Figure S11: Scatterplots of the 95% one-sided coverage probabilities against the relative median difference calculated on the probit scale. The value of the true R value is illustrated by the color scale. The size of the dots corresponds to the size of the sample size of effect. A vertical reference line is drawn at a coverage probability of 95%. The points corresponding to $n_y = 12$ are indicated by a black circle.

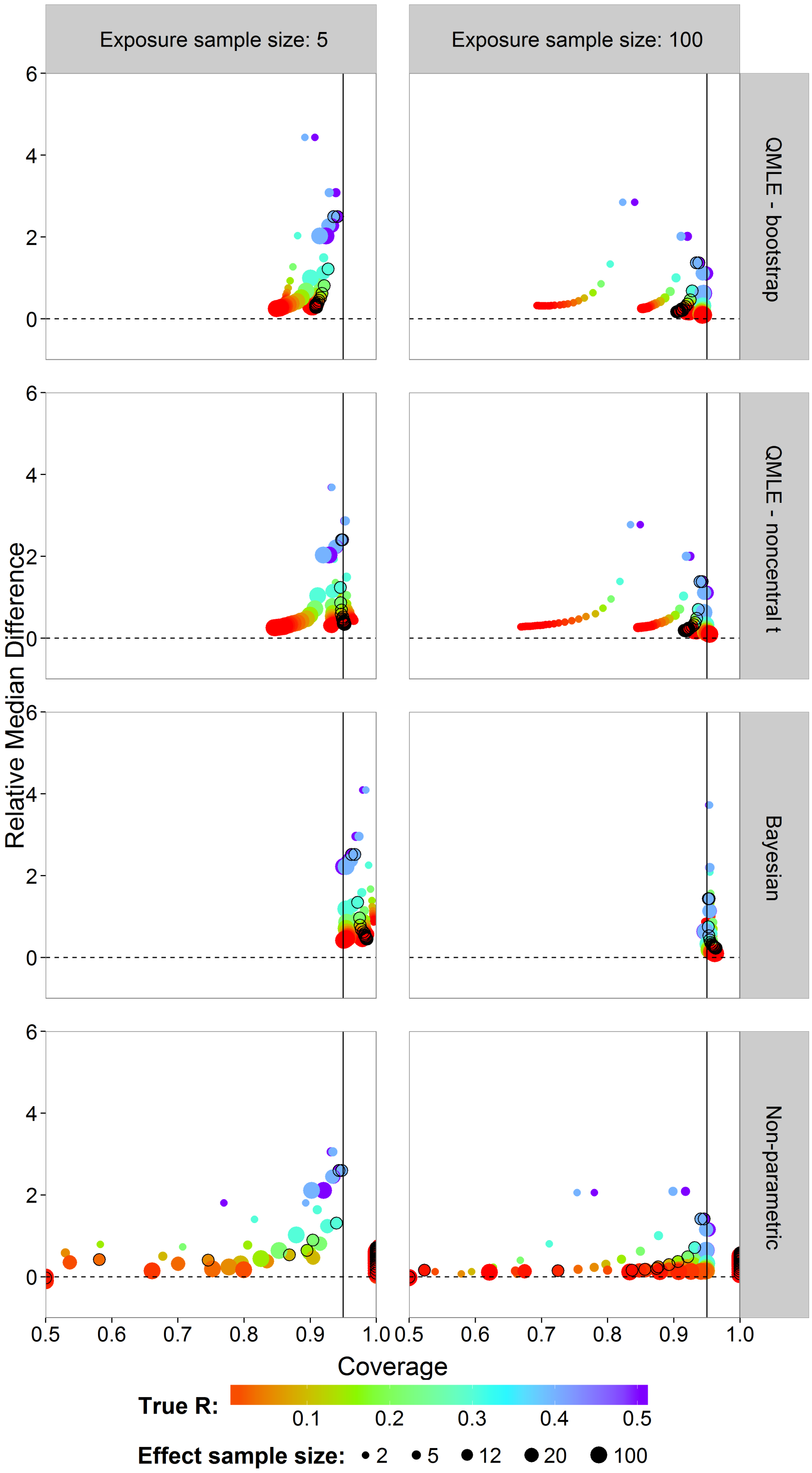

Table S2: The sample size, true R, true $probit(R)$, mean ($\bar{\hat{R}}$), standard deviation (SD), bias and root mean squared error (RMSE) of the MLE for all sample size scenarios and selected R values.

(n_x, n_y)	R	$probit(R)$	$\bar{\hat{R}}$ (SD)	bias	RMSE
(5,2)	0.5000	0.0000	-0.0139 (1.0115)	−0.0139	1.0116
	0.0966	−1.3014	-1.9791 (1.3210)	−0.6777	1.4847
	0.0021	−2.8632	-4.3374 (2.1167)	−1.4742	2.5795
	4.8e-06	−4.4249	-6.6957 (3.0421)	−2.2707	3.7962
	1.1e-09	−5.9867	-9.0539 (4.0085)	−3.0672	5.0474
	2.2e-14	−7.5484	-11.4122 (4.9920)	−3.8638	6.3126
(5,5)	0.5000	0.0000	-0.0047 (0.5838)	−0.0047	0.5838
	0.0966	−1.3014	-1.6239 (0.7537)	−0.3224	0.8197
	0.0021	−2.8632	-3.5669 (1.1882)	−0.7037	1.3810
	4.8e-06	−4.4249	-5.5100 (1.6967)	−1.0851	2.0140
	1.1e-09	−5.9867	-7.4530 (2.2292)	−1.4664	2.6682
	2.2e-14	−7.5484	-9.3961 (2.7718)	−1.8477	3.3312
(5,12)	0.5000	0.0000	-0.0003 (0.4373)	−0.0003	0.4373
	0.0966	−1.3014	-1.4901 (0.5304)	−0.1887	0.5629
	0.0021	−2.8632	-3.2779 (0.7997)	−0.4147	0.9008
	4.8e-06	−4.4249	-5.0656 (1.1273)	−0.6407	1.2966
	1.1e-09	−5.9867	-6.8534 (1.4747)	−0.8667	1.7105
	2.2e-14	−7.5484	-8.6411 (1.8308)	−1.0927	2.1321
(5,20)	0.5000	0.0000	0.0029 (0.4040)	0.0029	0.4040
	0.0966	−1.3014	-1.4525 (0.4823)	−0.1511	0.5054
	0.0021	−2.8632	-3.1991 (0.7104)	−0.3359	0.7859
	4.8e-06	−4.4249	-4.9457 (0.9914)	−0.5208	1.1198
	1.1e-09	−5.9867	-6.6923 (1.2911)	−0.7056	1.4713
	2.2e-14	−7.5484	-8.4388 (1.5991)	−0.8904	1.8303
(5,100)	0.5000	0.0000	0.0061 (0.3565)	0.0061	0.3565
	0.0966	−1.3014	-1.4208 (0.4181)	−0.1194	0.4348
	0.0021	−2.8632	-3.1331 (0.5940)	−0.2699	0.6525
	4.8e-06	−4.4249	-4.8454 (0.8141)	−0.4205	0.9163
	1.1e-09	−5.9867	-6.5577 (1.0508)	−0.5711	1.1960
	2.2e-14	−7.5484	-8.2701 (1.2952)	−0.7217	1.4827
(100,2)	0.5000	0.0000	-0.0131 (0.6229)	−0.0131	0.6230
	0.0966	−1.3014	-1.6115 (0.6814)	−0.3100	0.7486
	0.0021	−2.8632	-3.5295 (0.8675)	−0.6664	1.0939
	4.8e-06	−4.4249	-5.4476 (1.1203)	−1.0227	1.5169
	1.1e-09	−5.9867	-7.3657 (1.4042)	−1.3790	1.9681
	2.2e-14	−7.5484	-9.2837 (1.7039)	−1.7353	2.4320
(100,5)	0.5000	0.0000	0.0007 (0.3573)	0.0007	0.3573
	0.0966	−1.3014	-1.4213 (0.4144)	−0.1198	0.4313
	0.0021	−2.8632	-3.1277 (0.5879)	−0.2645	0.6447
	4.8e-06	−4.4249	-4.8341 (0.8073)	−0.4091	0.9051
	1.1e-09	−5.9867	-6.5405 (1.0440)	−0.5538	1.1818
	2.2e-14	−7.5484	-8.2469 (1.2885)	−0.6985	1.4656
(100,12)	0.5000	0.0000	0.0014 (0.2256)	0.0014	0.2256
	0.0966	−1.3014	-1.3535 (0.2709)	−0.0520	0.2758
	0.0021	−2.8632	-2.9793 (0.3953)	−0.1162	0.4120
	4.8e-06	−4.4249	-4.6052 (0.5479)	−0.1803	0.5768
	1.1e-09	−5.9867	-6.2311 (0.7107)	−0.2444	0.7515
	2.2e-14	−7.5484	-7.8570 (0.8780)	−0.3086	0.9307
(100,20)	0.5000	0.0000	0.0014 (0.1769)	0.0014	0.1769
	0.0966	−1.3014	-1.3360 (0.2121)	−0.0346	0.2149
	0.0021	−2.8632	-2.9410 (0.3101)	−0.0778	0.3197
	4.8e-06	−4.4249	-4.5459 (0.4304)	−0.1210	0.4471
	1.1e-09	−5.9867	-6.1509 (0.5588)	−0.1642	0.5824
	2.2e-14	−7.5484	-7.7558 (0.6907)	−0.2074	0.7212
(100,100)	0.5000	0.0000	-0.0032 (0.1003)	−0.0032	0.1003
	0.0966	−1.3014	-1.3152 (0.1198)	−0.0137	0.1206
	0.0021	−2.8632	-2.8896 (0.1750)	−0.0264	0.1770
	4.8e-06	−4.4249	-4.4640 (0.2430)	−0.0390	0.2461
	1.1e-09	−5.9867	-6.0384 (0.3156)	−0.0517	0.3198
	2.2e-14	−7.5484	-7.6128 (0.3902)	−0.0644	0.3955

Table S3: The sample size, true R, true $probit(R)$, mean ($\bar{\hat{R}}$), standard deviation (SD), bias and root mean squared error (RMSE) of the QMLE for all sample size scenarios and selected R values.

(n_x, n_y)	R	$probit(R)$	$\bar{\hat{R}}$ (SD)	bias	RMSE
(5,2)	0.5000	0.0000	-0.0112 (0.8466)	−0.0112	0.8467
	0.0966	−1.3014	-1.6454 (1.1225)	−0.3439	1.1740
	0.0021	−2.8632	-3.6064 (1.8236)	−0.7432	1.9692
	4.8e-06	−4.4249	-5.5674 (2.6331)	−1.1425	2.8703
	1.1e-09	−5.9867	-7.5285 (3.4761)	−1.5418	3.8027
	2.2e-14	−7.5484	-9.4895 (4.3330)	−1.9411	4.7479
(5,5)	0.5000	0.0000	-0.0042 (0.5222)	−0.0042	0.5222
	0.0966	−1.3014	-1.4524 (0.6741)	−0.1510	0.6908
	0.0021	−2.8632	-3.1904 (1.0628)	−0.3272	1.1120
	4.8e-06	−4.4249	-4.9283 (1.5176)	−0.5034	1.5989
	1.1e-09	−5.9867	-6.6662 (1.9938)	−0.6795	2.1065
	2.2e-14	−7.5484	-8.4041 (2.4791)	−0.8557	2.6227
(5,12)	0.5000	0.0000	-0.0003 (0.4068)	−0.0003	0.4068
	0.0966	−1.3014	-1.3845 (0.4970)	−0.0831	0.5039
	0.0021	−2.8632	-3.0456 (0.7554)	−0.1824	0.7771
	4.8e-06	−4.4249	-4.7066 (1.0680)	−0.2817	1.1045
	1.1e-09	−5.9867	-6.3677 (1.3988)	−0.3810	1.4498
	2.2e-14	−7.5484	-8.0288 (1.7376)	−0.4804	1.8028
(5,20)	0.5000	0.0000	0.0026 (0.3804)	0.0026	0.3804
	0.0966	−1.3014	-1.3657 (0.4612)	−0.0643	0.4656
	0.0021	−2.8632	-3.0078 (0.6908)	−0.1446	0.7057
	4.8e-06	−4.4249	-4.6498 (0.9702)	−0.2249	0.9959
	1.1e-09	−5.9867	-6.2918 (1.2668)	−0.3052	1.3030
	2.2e-14	−7.5484	-7.9338 (1.5710)	−0.3854	1.6176
(5,100)	0.5000	0.0000	0.0058 (0.3407)	0.0058	0.3407
	0.0966	−1.3014	-1.3547 (0.4104)	−0.0533	0.4139
	0.0021	−2.8632	-2.9874 (0.6043)	−0.1242	0.6170
	4.8e-06	−4.4249	-4.6201 (0.8415)	−0.1952	0.8638
	1.1e-09	−5.9867	-6.2528 (1.0941)	−0.2661	1.1260
	2.2e-14	−7.5484	-7.8854 (1.3535)	−0.3370	1.3948
(100,2)	0.5000	0.0000	-0.0121 (0.5763)	−0.0121	0.5765
	0.0966	−1.3014	-1.4736 (0.6726)	−0.1721	0.6943
	0.0021	−2.8632	-3.2273 (0.9523)	−0.3641	1.0195
	4.8e-06	−4.4249	-4.9810 (1.3036)	−0.5561	1.4172
	1.1e-09	−5.9867	-6.7347 (1.6823)	−0.7480	1.8411
	2.2e-14	−7.5484	-8.4884 (2.0734)	−0.9400	2.2766
(100,5)	0.5000	0.0000	0.0007 (0.3414)	0.0007	0.3414
	0.0966	−1.3014	-1.3549 (0.4068)	−0.0534	0.4103
	0.0021	−2.8632	-2.9816 (0.5989)	−0.1184	0.6105
	4.8e-06	−4.4249	-4.6083 (0.8358)	−0.1834	0.8557
	1.1e-09	−5.9867	-6.2351 (1.0887)	−0.2484	1.1166
	2.2e-14	−7.5484	-7.8618 (1.3485)	−0.3134	1.3844
(100,12)	0.5000	0.0000	0.0014 (0.2205)	0.0014	0.2205
	0.0966	−1.3014	-1.3229 (0.2672)	−0.0215	0.2681
	0.0021	−2.8632	-2.9121 (0.3942)	−0.0489	0.3973
	4.8e-06	−4.4249	-4.5012 (0.5489)	−0.0763	0.5542
	1.1e-09	−5.9867	-6.0904 (0.7135)	−0.1037	0.7210
	2.2e-14	−7.5484	-7.6796 (0.8824)	−0.1312	0.8921
(100,20)	0.5000	0.0000	0.0014 (0.1743)	0.0014	0.1743
	0.0966	−1.3014	-1.3162 (0.2099)	−0.0148	0.2104
	0.0021	−2.8632	-2.8974 (0.3084)	−0.0342	0.3102
	4.8e-06	−4.4249	-4.4786 (0.4289)	−0.0537	0.4322
	1.1e-09	−5.9867	-6.0598 (0.5573)	−0.0731	0.5621
	2.2e-14	−7.5484	-7.6409 (0.6892)	−0.0925	0.6954
(100,100)	0.5000	0.0000	-0.0032 (0.0998)	−0.0032	0.0998
	0.0966	−1.3014	-1.3086 (0.1192)	−0.0071	0.1194
	0.0021	−2.8632	-2.8751 (0.1741)	−0.0119	0.1745
	4.8e-06	−4.4249	-4.4416 (0.2417)	−0.0167	0.2423
	1.1e-09	−5.9867	-6.0081 (0.3140)	−0.0214	0.3147
	2.2e-14	−7.5484	-7.5746 (0.3883)	−0.0262	0.3891

Table S4: The sample size, true R, true $probit(R)$, mean ($\bar{\hat{R}}$), standard deviation (SD), bias and root mean squared error (RMSE) of the Bayes estimator for all sample size scenarios and selected R values.

(n_x, n_y)	R	$probit(R)$	$\bar{\hat{R}}$ (SD)	bias	RMSE
(5,2)	0.5000	0.0000	-0.0080 (0.6408)	−0.0080	0.6409
	0.0966	−1.3014	-1.2282 (0.8496)	0.0733	0.8528
	0.0021	−2.8632	-2.6603 (1.4094)	0.2029	1.4240
	4.8e-06	−4.4249	-4.0899 (2.0566)	0.3350	2.0837
	1.1e-09	−5.9867	-5.5223 (2.7272)	0.4643	2.7665
	2.2e-14	−7.5484	-6.9561 (3.4069)	0.5923	3.4580
(5,5)	0.5000	0.0000	-0.0037 (0.4523)	−0.0037	0.4523
	0.0966	−1.3014	-1.2547 (0.5796)	0.0468	0.5815
	0.0021	−2.8632	-2.7466 (0.9129)	0.1166	0.9203
	4.8e-06	−4.4249	-4.2376 (1.3041)	0.1873	1.3175
	1.1e-09	−5.9867	-5.7290 (1.7140)	0.2577	1.7332
	2.2e-14	−7.5484	-7.2208 (2.1317)	0.3276	2.1567
(5,12)	0.5000	0.0000	-0.0003 (0.3716)	−0.0003	0.3716
	0.0966	−1.3014	-1.2611 (0.4539)	0.0404	0.4557
	0.0021	−2.8632	-2.7691 (0.6922)	0.0941	0.6986
	4.8e-06	−4.4249	-4.2768 (0.9804)	0.1481	0.9915
	1.1e-09	−5.9867	-5.7848 (1.2852)	0.2018	1.3010
	2.2e-14	−7.5484	-7.2930 (1.5972)	0.2554	1.6175
(5,20)	0.5000	0.0000	0.0023 (0.3534)	0.0023	0.3535
	0.0966	−1.3014	-1.2668 (0.4310)	0.0347	0.4324
	0.0021	−2.8632	-2.7862 (0.6504)	0.0770	0.6549
	4.8e-06	−4.4249	-4.3054 (0.9161)	0.1196	0.9238
	1.1e-09	−5.9867	-5.8245 (1.1976)	0.1621	1.2086
	2.2e-14	−7.5484	-7.3440 (1.4860)	0.2044	1.5000
(5,100)	0.5000	0.0000	0.0056 (0.3249)	0.0056	0.3250
	0.0966	−1.3014	-1.2898 (0.3977)	0.0117	0.3979
	0.0021	−2.8632	-2.8445 (0.5977)	0.0187	0.5980
	4.8e-06	−4.4249	-4.3995 (0.8393)	0.0254	0.8397
	1.1e-09	−5.9867	-5.9547 (1.0953)	0.0320	1.0957
	2.2e-14	−7.5484	-7.5098 (1.3574)	0.0386	1.3579
(100,2)	0.5000	0.0000	-0.0105 (0.5136)	−0.0105	0.5137
	0.0966	−1.3014	-1.2868 (0.6395)	0.0147	0.6397
	0.0021	−2.8632	-2.7992 (1.0052)	0.0640	1.0072
	4.8e-06	−4.4249	-4.3101 (1.4448)	0.1148	1.4493
	1.1e-09	−5.9867	-5.8226 (1.9050)	0.1641	1.9121
	2.2e-14	−7.5484	-7.3361 (2.3732)	0.2123	2.3827
(100,5)	0.5000	0.0000	0.0008 (0.3255)	0.0008	0.3255
	0.0966	−1.3014	-1.2898 (0.3942)	0.0116	0.3944
	0.0021	−2.8632	-2.8387 (0.5928)	0.0244	0.5933
	4.8e-06	−4.4249	-4.3879 (0.8344)	0.0370	0.8352
	1.1e-09	−5.9867	-5.9371 (1.0909)	0.0496	1.0921
	2.2e-14	−7.5484	-7.4863 (1.3538)	0.0621	1.3552
(100,12)	0.5000	0.0000	0.0014 (0.2156)	0.0014	0.2156
	0.0966	−1.3014	-1.2928 (0.2620)	0.0086	0.2621
	0.0021	−2.8632	-2.8457 (0.3876)	0.0175	0.3880
	4.8e-06	−4.4249	-4.3987 (0.5402)	0.0263	0.5408
	1.1e-09	−5.9867	-5.9516 (0.7025)	0.0351	0.7033
	2.2e-14	−7.5484	-7.5045 (0.8689)	0.0439	0.8700
(100,20)	0.5000	0.0000	0.0014 (0.1716)	0.0014	0.1716
	0.0966	−1.3014	-1.2954 (0.2067)	0.0061	0.2068
	0.0021	−2.8632	-2.8512 (0.3038)	0.0120	0.3041
	4.8e-06	−4.4249	-4.4070 (0.4226)	0.0179	0.4229
	1.1e-09	−5.9867	-5.9628 (0.5490)	0.0239	0.5495
	2.2e-14	−7.5484	-7.5185 (0.6789)	0.0299	0.6796
(100,100)	0.5000	0.0000	-0.0031 (0.0991)	−0.0031	0.0992
	0.0966	−1.3014	-1.3002 (0.1184)	0.0012	0.1184
	0.0021	−2.8632	-2.8563 (0.1729)	0.0069	0.1731
	4.8e-06	−4.4249	-4.4124 (0.2401)	0.0125	0.2405
	1.1e-09	−5.9867	-5.9684 (0.3119)	0.0183	0.3124
	2.2e-14	−7.5484	-7.5245 (0.3857)	0.0239	0.3865

Table S5: The sample size, true R, true $probit(R)$, mean ($\bar{\hat{R}}$), standard deviation (SD), bias and root mean squared error (RMSE) of the non-parametric estimator for all sample size scenarios and selected R values.

(n_x, n_y)	R	$probit(R)$	$\bar{\hat{R}}$ (SD)	bias	RMSE
(5,2)	0.5000	0.0000	-0.0084 (0.6513)	−0.0084	0.6513
	0.0966	−1.3014	-1.0585 (0.4276)	0.2429	0.4918
	0.0021	−2.8632	-1.3759 (0.0594)	1.4873	1.4885
	4.8e-06	−4.4249	-1.3830 (0.0000)	3.0419	3.0419
	1.1e-09	−5.9867	-1.3830 (0.0000)	4.6037	4.6037
	2.2e-14	−7.5484	-1.3830 (0.0000)	6.1654	6.1654
(5,5)	0.5000	0.0000	-0.0037 (0.5129)	−0.0037	0.5130
	0.0966	−1.3014	-1.2666 (0.4514)	0.0349	0.4527
	0.0021	−2.8632	-1.7699 (0.0811)	1.0933	1.0963
	4.8e-06	−4.4249	-1.7862 (0.0000)	2.6388	2.6388
	1.1e-09	−5.9867	-1.7862 (0.0000)	4.2005	4.2005
	2.2e-14	−7.5484	-1.7862 (0.0000)	5.7622	5.7622
(5,12)	0.5000	0.0000	0.0006 (0.4164)	0.0006	0.4164
	0.0966	−1.3014	-1.3553 (0.4597)	−0.0538	0.4628
	0.0021	−2.8632	-2.1091 (0.1136)	0.7540	0.7625
	4.8e-06	−4.4249	-2.1411 (0.0044)	2.2838	2.2838
	1.1e-09	−5.9867	-2.1412 (0.0000)	3.8455	3.8455
	2.2e-14	−7.5484	-2.1412 (0.0000)	5.4072	5.4072
(5,20)	0.5000	0.0000	0.0044 (0.3933)	0.0044	0.3933
	0.0966	−1.3014	-1.3763 (0.4579)	−0.0748	0.4639
	0.0021	−2.8632	-2.2877 (0.1397)	0.5755	0.5922
	4.8e-06	−4.4249	-2.3335 (0.0091)	2.0915	2.0915
	1.1e-09	−5.9867	-2.3338 (0.0000)	3.6529	3.6529
	2.2e-14	−7.5484	-2.3338 (0.0000)	5.2146	5.2146
(5,100)	0.5000	0.0000	0.0062 (0.3500)	0.0062	0.3501
	0.0966	−1.3014	-1.4054 (0.4421)	−0.1040	0.4541
	0.0021	−2.8632	-2.7503 (0.2376)	0.1129	0.2631
	4.8e-06	−4.4249	-2.8787 (0.0204)	1.5462	1.5463
	1.1e-09	−5.9867	-2.8794 (0.0000)	3.1072	3.1072
	2.2e-14	−7.5484	-2.8794 (0.0000)	4.6690	4.6690
(100,2)	0.5000	0.0000	-0.0139 (0.6039)	−0.0139	0.6040
	0.0966	−1.3014	-1.5296 (0.6232)	−0.2281	0.6637
	0.0021	−2.8632	-2.5186 (0.1802)	0.3446	0.3888
	4.8e-06	−4.4249	-2.5789 (0.0098)	1.8460	1.8461
	1.1e-09	−5.9867	-2.5793 (0.0000)	3.4074	3.4074
	2.2e-14	−7.5484	-2.5793 (0.0000)	4.9691	4.9691
(100,5)	0.5000	0.0000	0.0009 (0.3515)	0.0009	0.3515
	0.0966	−1.3014	-1.4019 (0.4353)	−0.1004	0.4467
	0.0021	−2.8632	-2.7463 (0.2379)	0.1169	0.2651
	4.8e-06	−4.4249	-2.8790 (0.0104)	1.5459	1.5460
	1.1e-09	−5.9867	-2.8794 (0.0000)	3.1072	3.1072
	2.2e-14	−7.5484	-2.8794 (0.0000)	4.6690	4.6690
(100,12)	0.5000	0.0000	0.0025 (0.2257)	0.0025	0.2257
	0.0966	−1.3014	-1.3456 (0.2849)	−0.0442	0.2883
	0.0021	−2.8632	-2.8960 (0.2942)	−0.0328	0.2961
	4.8e-06	−4.4249	-3.1435 (0.0156)	1.2814	1.2815
	1.1e-09	−5.9867	-3.1445 (0.0000)	2.8422	2.8422
	2.2e-14	−7.5484	-3.1445 (0.0000)	4.4039	4.4039
(100,20)	0.5000	0.0000	0.0021 (0.1791)	0.0021	0.1791
	0.0966	−1.3014	-1.3303 (0.2215)	−0.0289	0.2234
	0.0021	−2.8632	-2.9462 (0.3093)	−0.0830	0.3202
	4.8e-06	−4.4249	-3.2889 (0.0215)	1.1360	1.1362
	1.1e-09	−5.9867	-3.2908 (0.0000)	2.6959	2.6959
	2.2e-14	−7.5484	-3.2908 (0.0000)	4.2576	4.2576
(100,100)	0.5000	0.0000	-0.0027 (0.1023)	−0.0027	0.1023
	0.0966	−1.3014	-1.3136 (0.1233)	−0.0122	0.1239
	0.0021	−2.8632	-2.9603 (0.3001)	−0.0971	0.3155
	4.8e-06	−4.4249	-3.7115 (0.0420)	0.7134	0.7147
	1.1e-09	−5.9867	-3.7191 (0.0000)	2.2676	2.2676
	2.2e-14	−7.5484	-3.7191 (0.0000)	3.8293	3.8293

2.3 Case: $\sigma_y = 0.2\sigma_x$

Figure S12: The median of the sampling distribution of the three Bayesian point estimators (mean, median and mode) calculated on the original scale and plotted on log_{10}-scale. When plotted on log_{10}-scale, a zero mode becomes $-\infty$. The diagonal dotted line represents the values where $\hat{R} = R$.

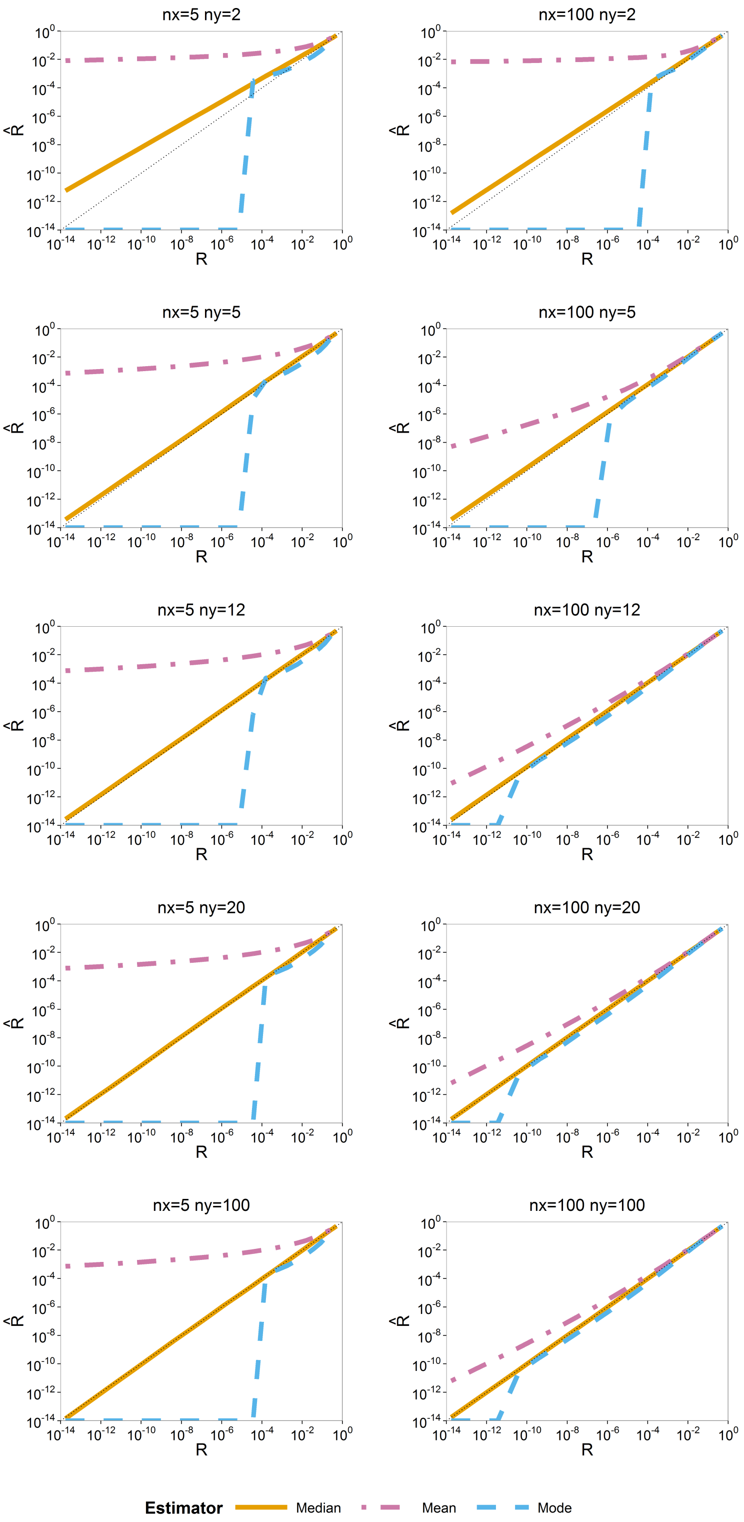

Figure S13: The 0.025 and 0.975 quantiles of the sampling distribution of the three Bayesian point estimators (mean, median and mode) calculated on the original scale and plotted on log_{10}-scale. When plotted on log_{10}-scale, a zero mode becomes $-\infty$. The diagonal dotted line represents the values where $\hat{R} = R$.

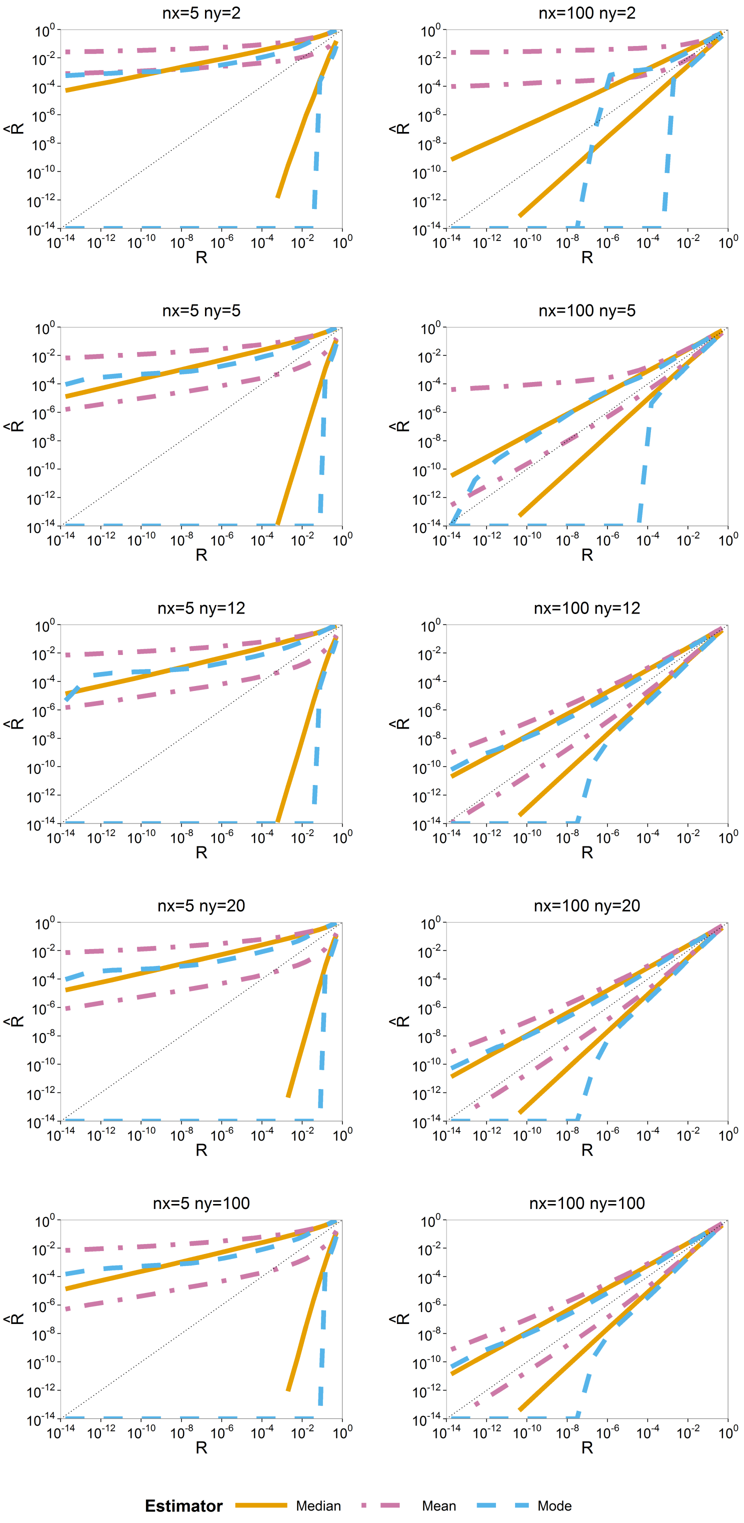

Figure S14: The median of the sampling distribution of the three Bayesian point estimators (mean, median and mode) calculated on the probit scale. The diagonal dotted line represents the values where $probit(\hat{R}) = probit(R)$.

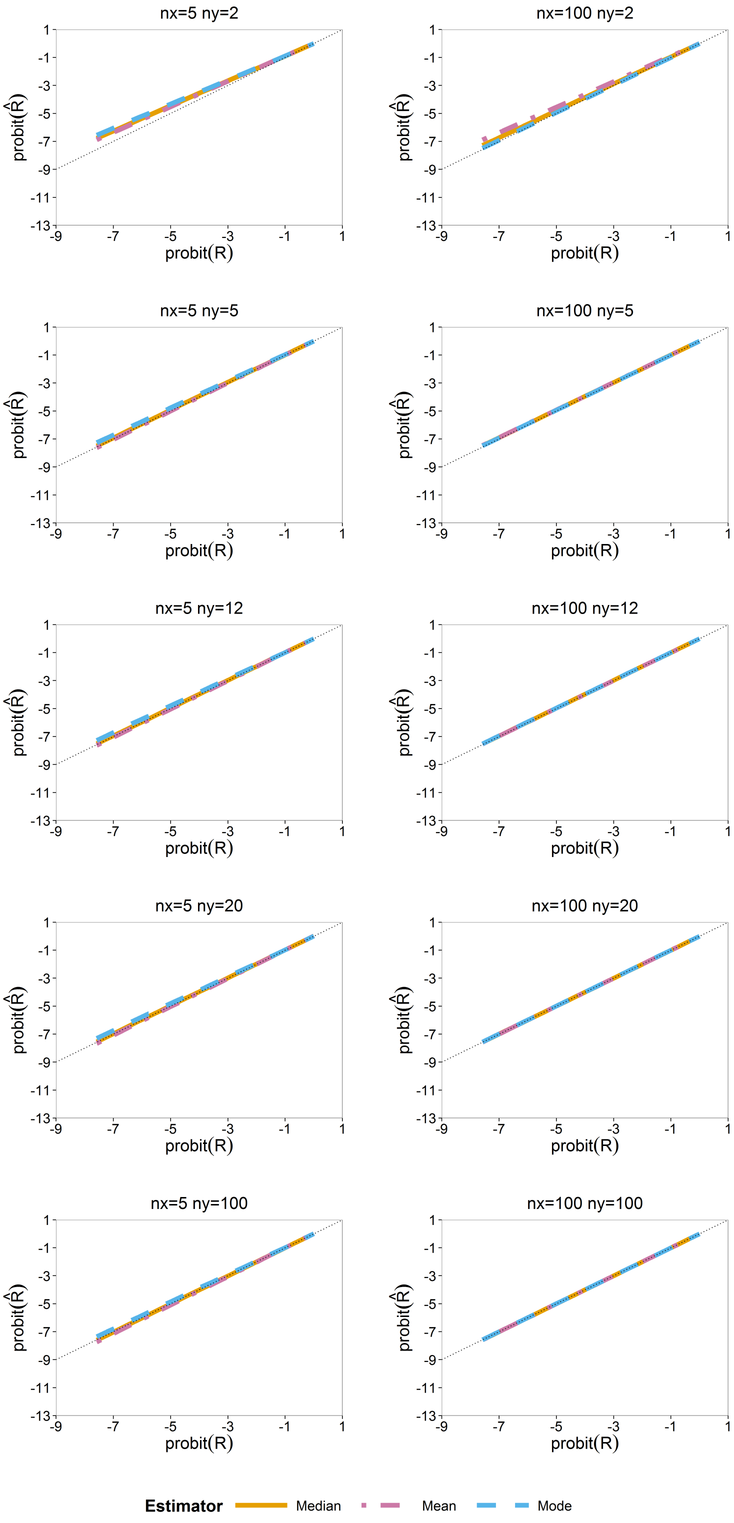

Figure S15: The 0.025 and 0.975 quantiles of the sampling distribution of the three Bayesian point estimators (mean, median and mode) calculated on the probit scale. The diagonal dotted line represents the values where $probit(\hat{R}) = probit(R)$.

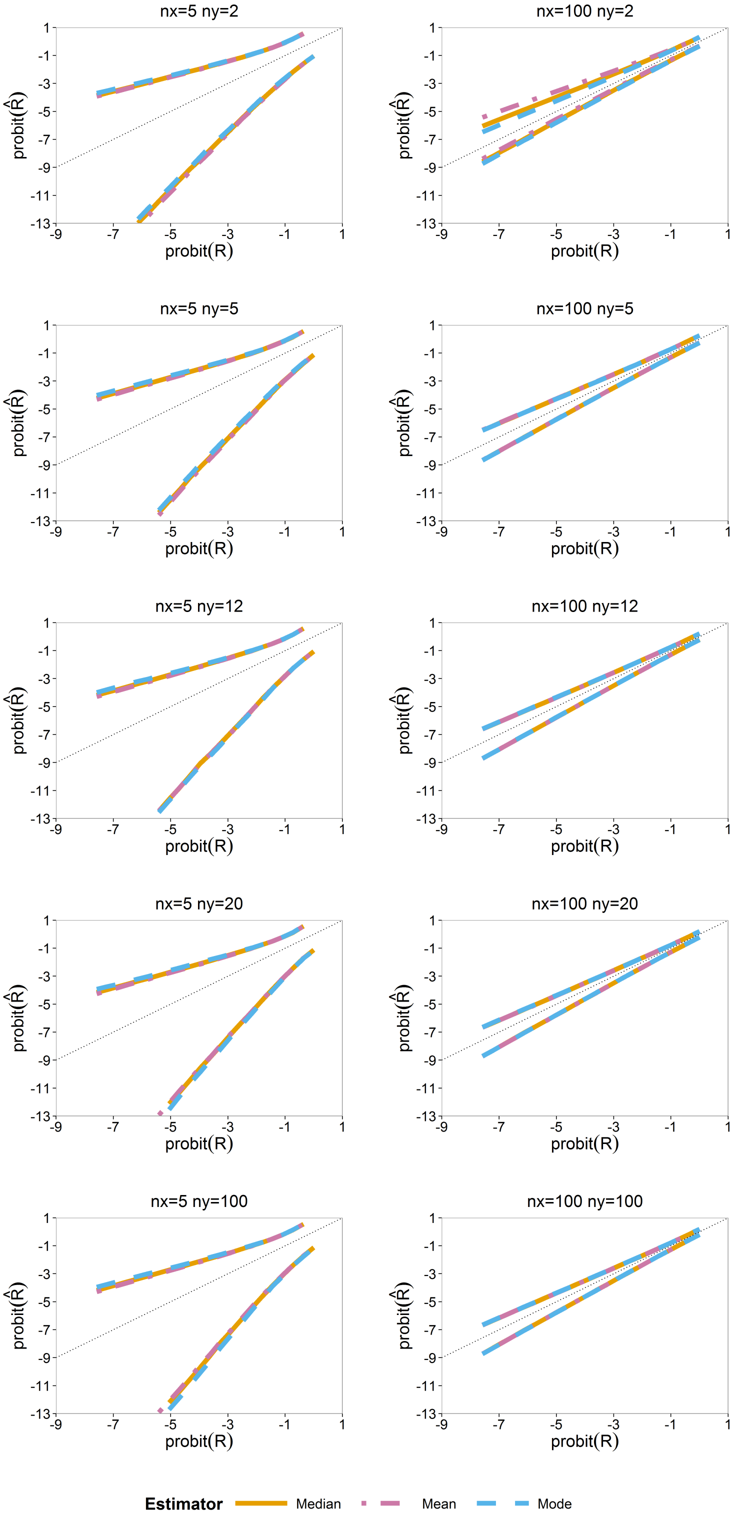

Figure S16: The median of the sampling distribution of the four point estimators, $\hat{R}_{MLE}$, $\hat{R}_{QMLE}$, $\hat{R}_{Bayes}$ and $\hat{R}_{NP}$ calculated on the probit scale. The diagonal dotted line represents the values where $probit(\hat{R}) = probit(R)$. The horizontal grey line gives the lower bound ($probit\left(\frac{1}{n_x n_y+2}\right)$) of $probit(\hat{R}_{NP})$.

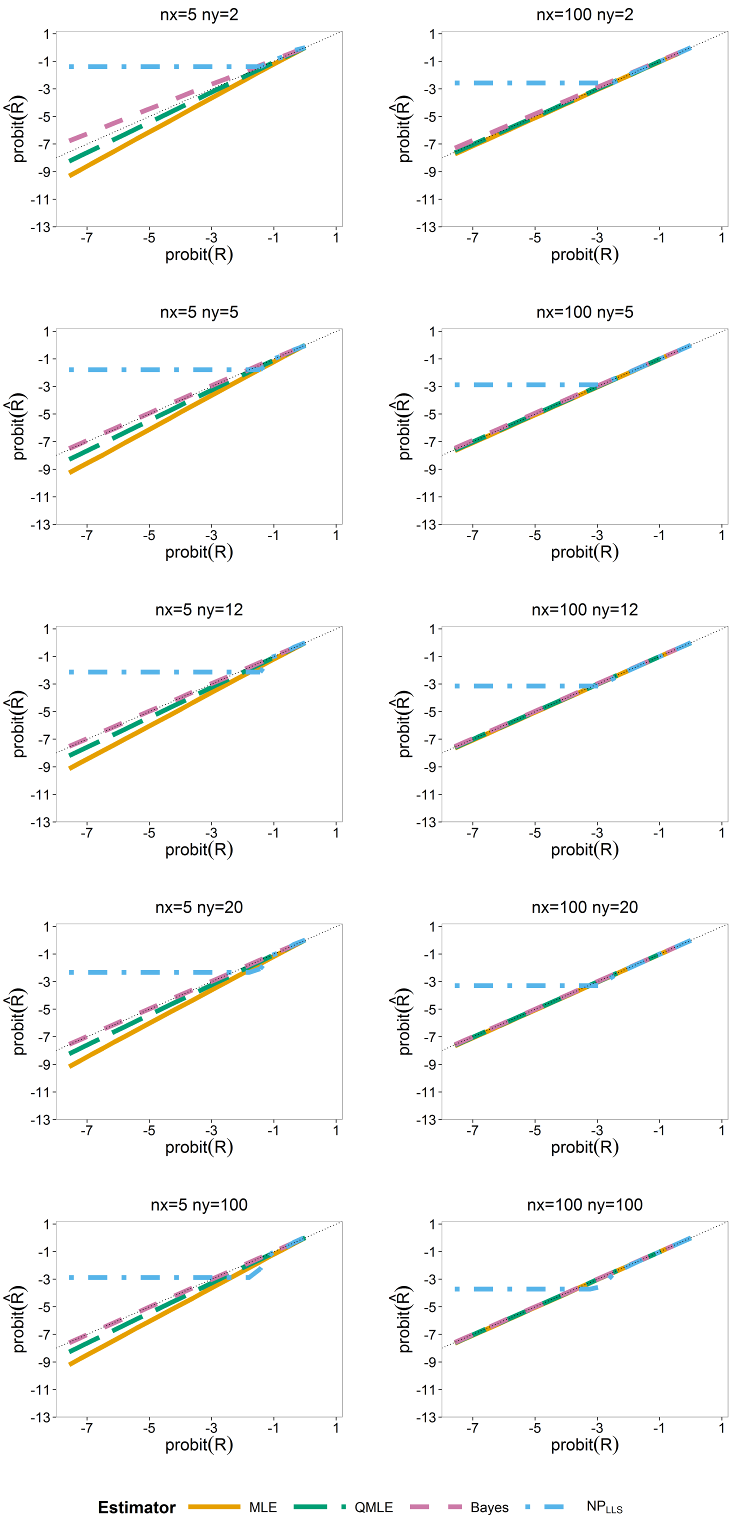

Figure S17: The 0.025 and 0.975 quantiles of the sampling distribution of the four point estimators, $\hat{R}_{MLE}$, $\hat{R}_{QMLE}$, $\hat{R}_{Bayes}$ and $\hat{R}_{NP}$ calculated on the probit scale. The diagonal dotted line represents the values where $probit(\hat{R}) = probit(R)$. The horizontal grey line gives the lower bound ($probit\left(\frac{1}{n_x n_y+2}\right)$) of $probit(\hat{R}_{NP})$.

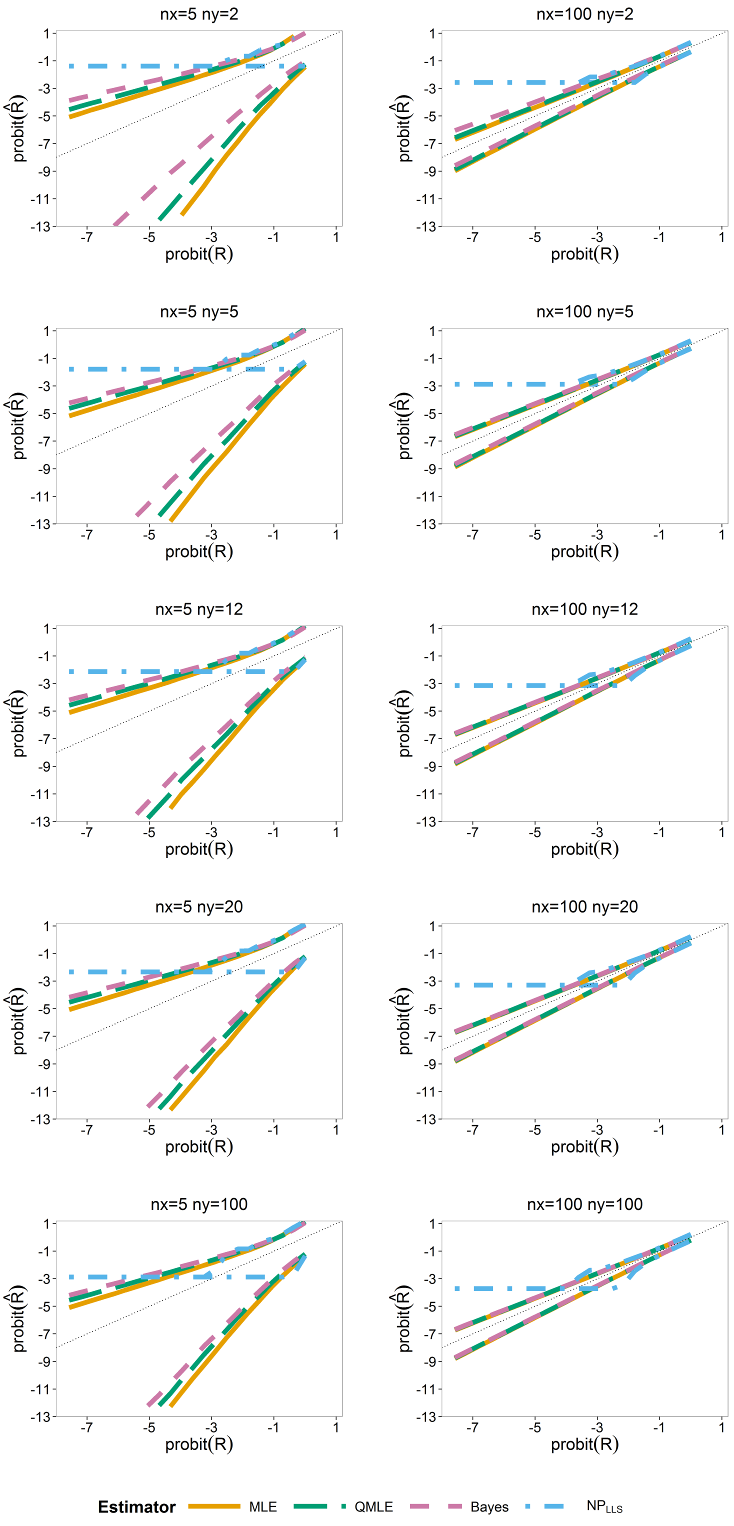

Figure S18: Scatterplots of the 90% two-sided coverage probabilities against the relative median interval length calculated on the probit scale. The value of the true R value is illustrated by the color scale. The size of the dots corresponds to the size of the sample size of effect. A vertical reference line is drawn at a coverage probability of 90%. The points corresponding to $n_y = 12$ are indicated by an open black circle.

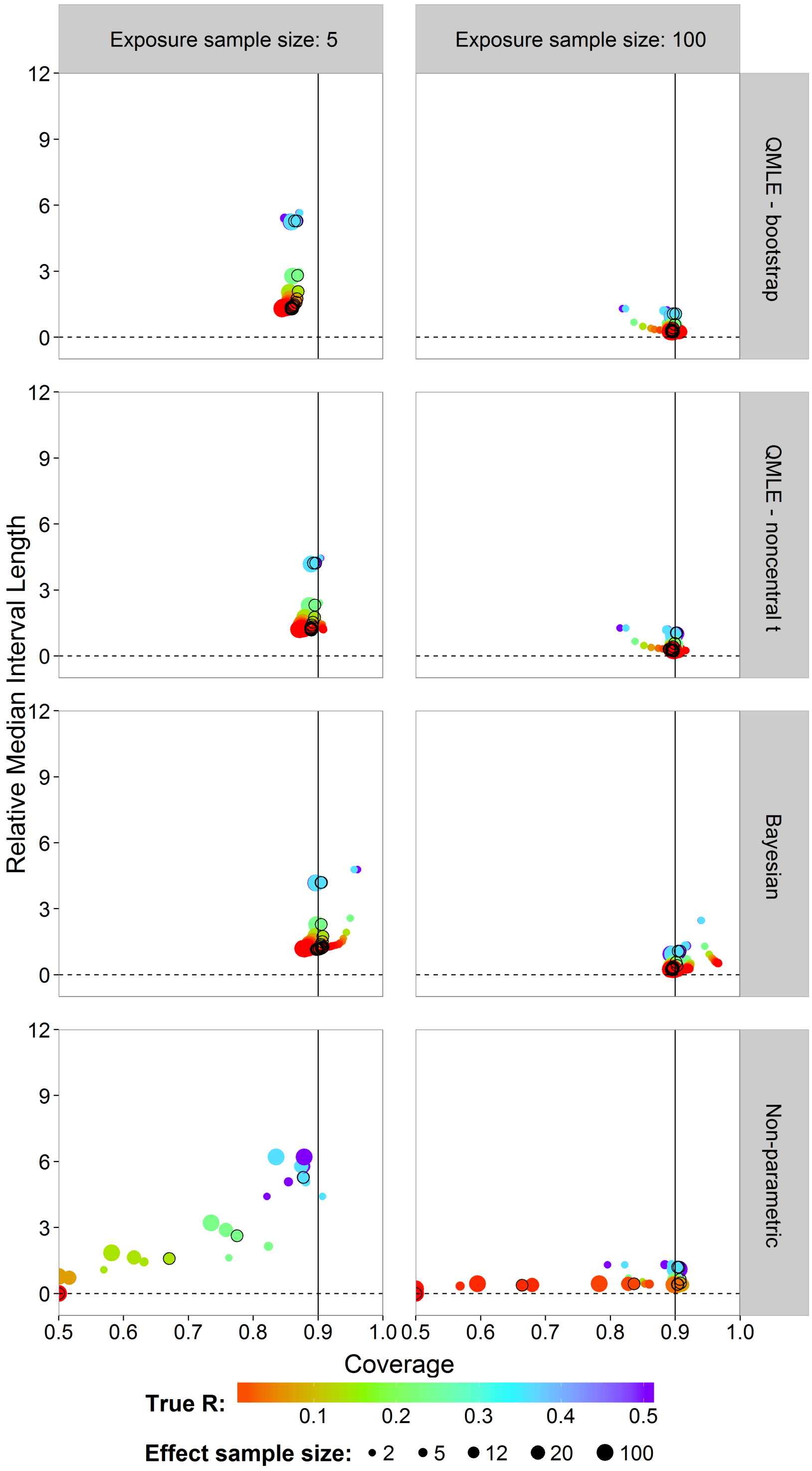

Figure S19: Scatterplots of the 95% one-sided coverage probabilities against the relative median difference calculated on the probit scale. The value of the true R value is illustrated by the color scale. The size of the dots corresponds to the size of the sample size of effect. A vertical reference line is drawn at a coverage probability of 95%. The points corresponding to $n_y = 12$ are indicated by a black circle.

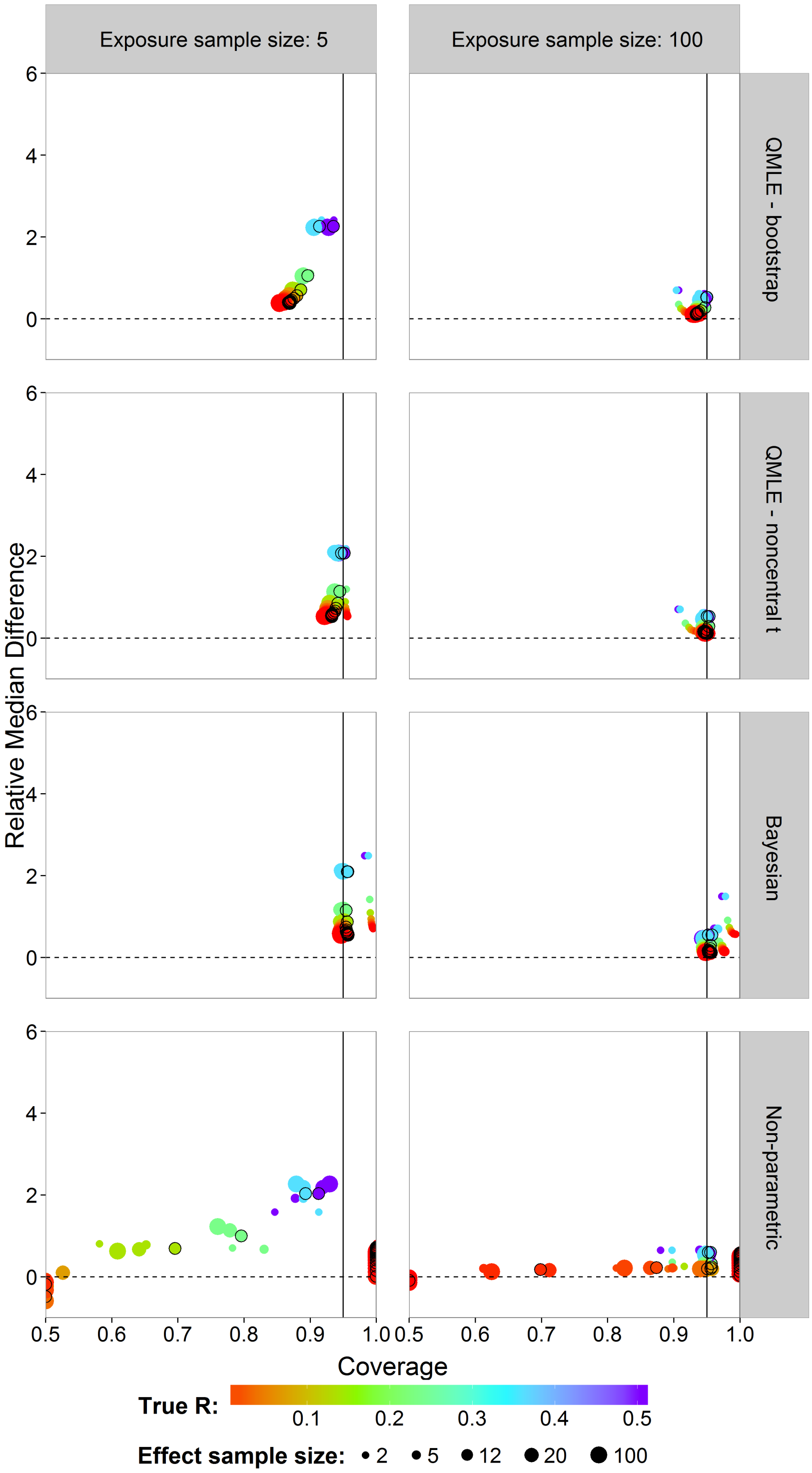

Table S6: The sample size, true R, true $probit(R)$, mean ($\bar{\hat{R}}$), standard deviation (SD), bias and root mean squared error (RMSE) of the MLE for all sample size scenarios and selected R values.

(n_x, n_y)	R	$probit(R)$	$\bar{\hat{R}}$ (SD)	bias	RMSE
(5,2)	0.5000	0.0000	-0.0026 (0.7379)	−0.0026	0.7379
	0.0744	−1.4438	-1.9999 (1.2222)	−0.5561	1.3428
	0.0006	−3.2486	-4.4966 (2.2665)	−1.2480	2.5874
	2.2e-07	−5.0534	-6.9932 (3.3927)	−1.9398	3.9081
	3.5e-12	−6.8582	-9.4898 (4.5403)	−2.6317	5.2478
(5,5)	0.5000	0.0000	-0.0141 (0.6726)	−0.0141	0.6728
	0.0744	−1.4438	-1.9827 (1.1013)	−0.5389	1.2261
	0.0006	−3.2486	-4.4435 (2.0338)	−1.1949	2.3589
	2.2e-07	−5.0534	-6.9043 (3.0417)	−1.8509	3.5606
	3.5e-12	−6.8582	-9.3651 (4.0694)	−2.5069	4.7796
(5,12)	0.5000	0.0000	0.0035 (0.6259)	0.0035	0.6259
	0.0744	−1.4438	-1.9206 (0.9807)	−0.4768	1.0904
	0.0006	−3.2486	-4.3258 (1.8348)	−1.0772	2.1276
	2.2e-07	−5.0534	-6.7310 (2.7661)	−1.6776	3.2350
	3.5e-12	−6.8582	-9.1361 (3.7171)	−2.2780	4.3595
(5,20)	0.5000	0.0000	0.0018 (0.6354)	0.0018	0.6354
	0.0744	−1.4438	-1.9312 (1.0372)	−0.4874	1.1460
	0.0006	−3.2486	-4.3474 (1.9211)	−1.0988	2.2131
	2.2e-07	−5.0534	-6.7636 (2.8770)	−1.7102	3.3469
	3.5e-12	−6.8582	-9.1798 (3.8517)	−2.3216	4.4973
(5,100)	0.5000	0.0000	0.0095 (0.6357)	0.0095	0.6357
	0.0744	−1.4438	-1.9328 (1.0125)	−0.4889	1.1244
	0.0006	−3.2486	-4.3606 (1.8890)	−1.1120	2.1920
	2.2e-07	−5.0534	-6.7885 (2.8416)	−1.7351	3.3294
	3.5e-12	−6.8582	-9.2163 (3.8137)	−2.3581	4.4839
(100,2)	0.5000	0.0000	-0.0008 (0.1720)	−0.0008	0.1720
	0.0744	−1.4438	-1.4773 (0.1997)	−0.0335	0.2024
	0.0006	−3.2486	-3.3230 (0.2906)	−0.0744	0.3000
	2.2e-07	−5.0534	-5.1687 (0.4053)	−0.1153	0.4214
	3.5e-12	−6.8582	-7.0144 (0.5286)	−0.1562	0.5512
(100,5)	0.5000	0.0000	0.0005 (0.1341)	0.0005	0.1341
	0.0744	−1.4438	-1.4654 (0.1690)	−0.0215	0.1704
	0.0006	−3.2486	-3.2977 (0.2675)	−0.0491	0.2719
	2.2e-07	−5.0534	-5.1301 (0.3841)	−0.0767	0.3917
	3.5e-12	−6.8582	-6.9625 (0.5065)	−0.1043	0.5172
(100,12)	0.5000	0.0000	0.0029 (0.1161)	0.0029	0.1161
	0.0744	−1.4438	-1.4601 (0.1559)	−0.0163	0.1568
	0.0006	−3.2486	-3.2889 (0.2583)	−0.0403	0.2615
	2.2e-07	−5.0534	-5.1177 (0.3758)	−0.0643	0.3813
	3.5e-12	−6.8582	-6.9465 (0.4978)	−0.0883	0.5056
(100,20)	0.5000	0.0000	-0.0015 (0.1078)	−0.0015	0.1079
	0.0744	−1.4438	-1.4653 (0.1500)	−0.0215	0.1516
	0.0006	−3.2486	-3.2952 (0.2543)	−0.0466	0.2585
	2.2e-07	−5.0534	-5.1250 (0.3722)	−0.0716	0.3790
	3.5e-12	−6.8582	-6.9549 (0.4941)	−0.0967	0.5035
(100,100)	0.5000	0.0000	-0.0031 (0.1016)	−0.0031	0.1016
	0.0744	−1.4438	-1.4642 (0.1440)	−0.0203	0.1454
	0.0006	−3.2486	-3.2905 (0.2489)	−0.0418	0.2524
	2.2e-07	−5.0534	-5.1167 (0.3668)	−0.0633	0.3722
	3.5e-12	−6.8582	-6.9430 (0.4883)	−0.0849	0.4956

Table S7: The sample size, true R, true $probit(R)$, mean ($\bar{\hat{R}}$), standard deviation (SD), bias and root mean squared error (RMSE) of the QMLE for all sample size scenarios and selected R values.

(n_x, n_y)	R	$probit(R)$	$\bar{\hat{R}}$ (SD)	bias	RMSE
(5,2)	0.5000	0.0000	-0.0018 (0.6419)	−0.0018	0.6419
	0.0744	−1.4438	-1.7627 (1.0435)	−0.3189	1.0911
	0.0006	−3.2486	-3.9640 (1.9285)	−0.7154	2.0569
	2.2e-07	−5.0534	-6.1652 (2.8866)	−1.1118	3.0933
	3.5e-12	−6.8582	-8.3664 (3.8637)	−1.5082	4.1476
(5,5)	0.5000	0.0000	-0.0126 (0.6016)	−0.0126	0.6017
	0.0744	−1.4438	-1.7734 (0.9851)	−0.3296	1.0387
	0.0006	−3.2486	-3.9744 (1.8191)	−0.7258	1.9585
	2.2e-07	−5.0534	-6.1754 (2.7206)	−1.1220	2.9429
	3.5e-12	−6.8582	-8.3764 (3.6397)	−1.5182	3.9437
(5,12)	0.5000	0.0000	0.0033 (0.5645)	0.0033	0.5645
	0.0744	−1.4438	-1.7285 (0.8925)	−0.2847	0.9368
	0.0006	−3.2486	-3.8933 (1.6777)	−0.6447	1.7973
	2.2e-07	−5.0534	-6.0580 (2.5321)	−1.0046	2.7241
	3.5e-12	−6.8582	-8.2228 (3.4040)	−1.3646	3.6673
(5,20)	0.5000	0.0000	0.0014 (0.5748)	0.0014	0.5748
	0.0744	−1.4438	-1.7423 (0.9515)	−0.2985	0.9973
	0.0006	−3.2486	-3.9220 (1.7727)	−0.6734	1.8963
	2.2e-07	−5.0534	-6.1017 (2.6583)	−1.0483	2.8575
	3.5e-12	−6.8582	-8.2813 (3.5606)	−1.4232	3.8345
(5,100)	0.5000	0.0000	0.0086 (0.5777)	0.0086	0.5778
	0.0744	−1.4438	-1.7476 (0.9347)	−0.3038	0.9828
	0.0006	−3.2486	-3.9428 (1.7562)	−0.6942	1.8884
	2.2e-07	−5.0534	-6.1380 (2.6460)	−1.0846	2.8597
	3.5e-12	−6.8582	-8.3332 (3.5532)	−1.4751	3.8472
(100,2)	0.5000	0.0000	-0.0008 (0.1696)	−0.0008	0.1696
	0.0744	−1.4438	-1.4566 (0.1986)	−0.0128	0.1990
	0.0006	−3.2486	-3.2763 (0.2920)	−0.0277	0.2933
	2.2e-07	−5.0534	-5.0961 (0.4090)	−0.0427	0.4112
	3.5e-12	−6.8582	-6.9158 (0.5342)	−0.0576	0.5373
(100,5)	0.5000	0.0000	0.0005 (0.1329)	0.0005	0.1329
	0.0744	−1.4438	-1.4526 (0.1675)	−0.0087	0.1677
	0.0006	−3.2486	-3.2689 (0.2650)	−0.0203	0.2658
	2.2e-07	−5.0534	-5.0853 (0.3806)	−0.0319	0.3819
	3.5e-12	−6.8582	-6.9017 (0.5019)	−0.0435	0.5038
(100,12)	0.5000	0.0000	0.0029 (0.1153)	0.0029	0.1154
	0.0744	−1.4438	-1.4507 (0.1548)	−0.0069	0.1550
	0.0006	−3.2486	-3.2677 (0.2563)	−0.0191	0.2570
	2.2e-07	−5.0534	-5.0846 (0.3728)	−0.0312	0.3741
	3.5e-12	−6.8582	-6.9016 (0.4938)	−0.0434	0.4957
(100,20)	0.5000	0.0000	-0.0015 (0.1072)	−0.0015	0.1072
	0.0744	−1.4438	-1.4568 (0.1491)	−0.0130	0.1497
	0.0006	−3.2486	-3.2761 (0.2525)	−0.0275	0.2540
	2.2e-07	−5.0534	-5.0953 (0.3696)	−0.0419	0.3720
	3.5e-12	−6.8582	-6.9145 (0.4906)	−0.0563	0.4938
(100,100)	0.5000	0.0000	-0.0031 (0.1011)	−0.0031	0.1011
	0.0744	−1.4438	-1.4568 (0.1432)	−0.0130	0.1438
	0.0006	−3.2486	-3.2740 (0.2476)	−0.0253	0.2489
	2.2e-07	−5.0534	-5.0911 (0.3649)	−0.0377	0.3669
	3.5e-12	−6.8582	-6.9082 (0.4859)	−0.0501	0.4884

Table S8: The sample size, true R, true $probit(R)$, mean $(\bar{\hat{R}})$, standard deviation (SD), bias and root mean squared error (RMSE) of the Bayes estimator for all sample size scenarios and selected R values.

(n_x, n_y)	R	$probit(R)$	$\bar{\hat{R}}$ (SD)	bias	RMSE
(5,2)	0.5000	0.0000	-0.0010 (0.5235)	−0.0010	0.5235
	0.0744	−1.4438	-1.4531 (0.8106)	−0.0092	0.8106
	0.0006	−3.2486	-3.2427 (1.4823)	0.0059	1.4823
	2.2e-07	−5.0534	-5.0336 (2.2163)	0.0198	2.2164
	3.5e-12	−6.8582	-6.8258 (2.9658)	0.0323	2.9660
(5,5)	0.5000	0.0000	-0.0111 (0.5406)	−0.0111	0.5407
	0.0744	−1.4438	-1.5968 (0.8564)	−0.1529	0.8699
	0.0006	−3.2486	-3.5638 (1.5670)	−0.3151	1.5984
	2.2e-07	−5.0534	-5.5314 (2.3400)	−0.4780	2.3883
	3.5e-12	−6.8582	-7.4998 (3.1289)	−0.6416	3.1941
(5,12)	0.5000	0.0000	0.0030 (0.5208)	0.0030	0.5208
	0.0744	−1.4438	-1.5901 (0.8090)	−0.1463	0.8221
	0.0006	−3.2486	-3.5658 (1.5191)	−0.3172	1.5518
	2.2e-07	−5.0534	-5.5420 (2.2940)	−0.4886	2.3455
	3.5e-12	−6.8582	-7.5193 (3.0847)	−0.6611	3.1548
(5,20)	0.5000	0.0000	0.0014 (0.5337)	0.0014	0.5337
	0.0744	−1.4438	-1.6105 (0.8737)	−0.1666	0.8894
	0.0006	−3.2486	-3.6087 (1.6309)	−0.3601	1.6702
	2.2e-07	−5.0534	-5.6073 (2.4488)	−0.5539	2.5106
	3.5e-12	−6.8582	-7.6071 (3.2816)	−0.7489	3.3660
(5,100)	0.5000	0.0000	0.0083 (0.5405)	0.0083	0.5405
	0.0744	−1.4438	-1.6231 (0.8713)	−0.1792	0.8896
	0.0006	−3.2486	-3.6445 (1.6452)	−0.3959	1.6922
	2.2e-07	−5.0534	-5.6665 (2.4835)	−0.6131	2.5581
	3.5e-12	−6.8582	-7.6897 (3.3373)	−0.8315	3.4393
(100,2)	0.5000	0.0000	-0.0007 (0.1626)	−0.0007	0.1626
	0.0744	−1.4438	-1.3939 (0.1998)	0.0499	0.2060
	0.0006	−3.2486	-3.1335 (0.3099)	0.1151	0.3305
	2.2e-07	−5.0534	-4.8727 (0.4417)	0.1807	0.4772
	3.5e-12	−6.8582	-6.6116 (0.5810)	0.2465	0.6311
(100,5)	0.5000	0.0000	0.0005 (0.1311)	0.0005	0.1311
	0.0744	−1.4438	-1.4320 (0.1652)	0.0118	0.1656
	0.0006	−3.2486	-3.2201 (0.2616)	0.0285	0.2632
	2.2e-07	−5.0534	-5.0078 (0.3760)	0.0456	0.3788
	3.5e-12	−6.8582	-6.7955 (0.4961)	0.0627	0.5001
(100,12)	0.5000	0.0000	0.0028 (0.1146)	0.0028	0.1146
	0.0744	−1.4438	-1.4401 (0.1535)	0.0037	0.1535
	0.0006	−3.2486	-3.2434 (0.2537)	0.0052	0.2537
	2.2e-07	−5.0534	-5.0467 (0.3688)	0.0067	0.3689
	3.5e-12	−6.8582	-6.8499 (0.4884)	0.0083	0.4885
(100,20)	0.5000	0.0000	-0.0015 (0.1066)	−0.0015	0.1067
	0.0744	−1.4438	-1.4489 (0.1480)	−0.0051	0.1481
	0.0006	−3.2486	-3.2580 (0.2504)	−0.0094	0.2506
	2.2e-07	−5.0534	-5.0671 (0.3664)	−0.0137	0.3666
	3.5e-12	−6.8582	-6.8762 (0.4862)	−0.0180	0.4866
(100,100)	0.5000	0.0000	-0.0031 (0.1008)	−0.0031	0.1008
	0.0744	−1.4438	-1.4519 (0.1427)	−0.0080	0.1429
	0.0006	−3.2486	-3.2623 (0.2467)	−0.0136	0.2471
	2.2e-07	−5.0534	-5.0726 (0.3635)	−0.0193	0.3641
	3.5e-12	−6.8582	-6.8831 (0.4840)	−0.0249	0.4847

Table S9: The sample size, true R, true $probit(R)$, mean ($\bar{\hat{R}}$), standard deviation (SD), bias and root mean squared error (RMSE) of the non-parametric estimator for all sample size scenarios and selected R values.

(n_x, n_y)	R	$probit(R)$	$\bar{\hat{R}}$ (SD)	bias	RMSE
(5,2)	0.5000	0.0000	0.0024 (0.5273)	0.0024	0.5273
	0.0744	−1.4438	-1.1263 (0.3676)	0.3176	0.4858
	0.0006	−3.2486	-1.3808 (0.0371)	1.8678	1.8682
	2.2e-07	−5.0534	-1.3830 (0.0000)	3.6704	3.6704
	3.5e-12	−6.8582	-1.3830 (0.0000)	5.4752	5.4752
(5,5)	0.5000	0.0000	-0.0109 (0.5885)	−0.0109	0.5886
	0.0744	−1.4438	-1.4070 (0.4858)	0.0368	0.4872
	0.0006	−3.2486	-1.7833 (0.0416)	1.4653	1.4659
	2.2e-07	−5.0534	-1.7862 (0.0000)	3.2672	3.2672
	3.5e-12	−6.8582	-1.7862 (0.0000)	5.0720	5.0720
(5,12)	0.5000	0.0000	0.0023 (0.6123)	0.0023	0.6123
	0.0744	−1.4438	-1.6243 (0.6131)	−0.1805	0.6391
	0.0006	−3.2486	-2.1354 (0.0716)	1.1132	1.1155
	2.2e-07	−5.0534	-2.1412 (0.0000)	2.9122	2.9122
	3.5e-12	−6.8582	-2.1412 (0.0000)	4.7170	4.7170
(5,20)	0.5000	0.0000	0.0047 (0.6235)	0.0047	0.6235
	0.0744	−1.4438	-1.7203 (0.6916)	−0.2765	0.7448
	0.0006	−3.2486	-2.3272 (0.0819)	0.9214	0.9251
	2.2e-07	−5.0534	-2.3338 (0.0000)	2.7196	2.7196
	3.5e-12	−6.8582	-2.3338 (0.0000)	4.5244	4.5244
(5,100)	0.5000	0.0000	0.0091 (0.6515)	0.0091	0.6516
	0.0744	−1.4438	-1.9859 (0.9037)	−0.5421	1.0538
	0.0006	−3.2486	-2.8670 (0.1244)	0.3816	0.4014
	2.2e-07	−5.0534	-2.8790 (0.0277)	2.1744	2.1746
	3.5e-12	−6.8582	-2.8794 (0.0000)	3.9788	3.9788
(100,2)	0.5000	0.0000	-0.0032 (0.1811)	−0.0032	0.1811
	0.0744	−1.4438	-1.4490 (0.2257)	−0.0052	0.2257
	0.0006	−3.2486	-2.5535 (0.0914)	0.6951	0.7011
	2.2e-07	−5.0534	-2.5793 (0.0000)	2.4741	2.4741
	3.5e-12	−6.8582	-2.5793 (0.0000)	4.2789	4.2789
(100,5)	0.5000	0.0000	0.0006 (0.1465)	0.0006	0.1465
	0.0744	−1.4438	-1.4577 (0.1993)	−0.0138	0.1998
	0.0006	−3.2486	-2.8367 (0.1363)	0.4119	0.4338
	2.2e-07	−5.0534	-2.8794 (0.0000)	2.1740	2.1740
	3.5e-12	−6.8582	-2.8794 (0.0000)	3.9788	3.9788
(100,12)	0.5000	0.0000	0.0033 (0.1291)	0.0033	0.1291
	0.0744	−1.4438	-1.4581 (0.1865)	−0.0143	0.1871
	0.0006	−3.2486	-3.0798 (0.1867)	0.1688	0.2517
	2.2e-07	−5.0534	-3.1445 (0.0000)	1.9089	1.9089
	3.5e-12	−6.8582	-3.1445 (0.0000)	3.7137	3.7137
(100,20)	0.5000	0.0000	-0.0004 (0.1227)	−0.0004	0.1227
	0.0744	−1.4438	-1.4686 (0.1804)	−0.0248	0.1821
	0.0006	−3.2486	-3.2080 (0.2246)	0.0406	0.2283
	2.2e-07	−5.0534	-3.2908 (0.0000)	1.7626	1.7626
	3.5e-12	−6.8582	-3.2908 (0.0000)	3.5674	3.5674
(100,100)	0.5000	0.0000	-0.0025 (0.1153)	−0.0025	0.1154
	0.0744	−1.4438	-1.4701 (0.1802)	−0.0262	0.1821
	0.0006	−3.2486	-3.5579 (0.3598)	−0.3093	0.4744
	2.2e-07	−5.0534	-3.7190 (0.0027)	1.3344	1.3344
	3.5e-12	−6.8582	-3.7191 (0.0000)	3.1391	3.1391

2.4 Case: $\sigma_y = 5\sigma_x$

Figure S20: The median of the sampling distribution of the three Bayesian point estimators (mean, median and mode) calculated on the original scale and plotted on log_{10}-scale. When plotted on log_{10}-scale, a zero mode becomes $-\infty$. The diagonal dotted line represents the values where $\hat{R} = R$.

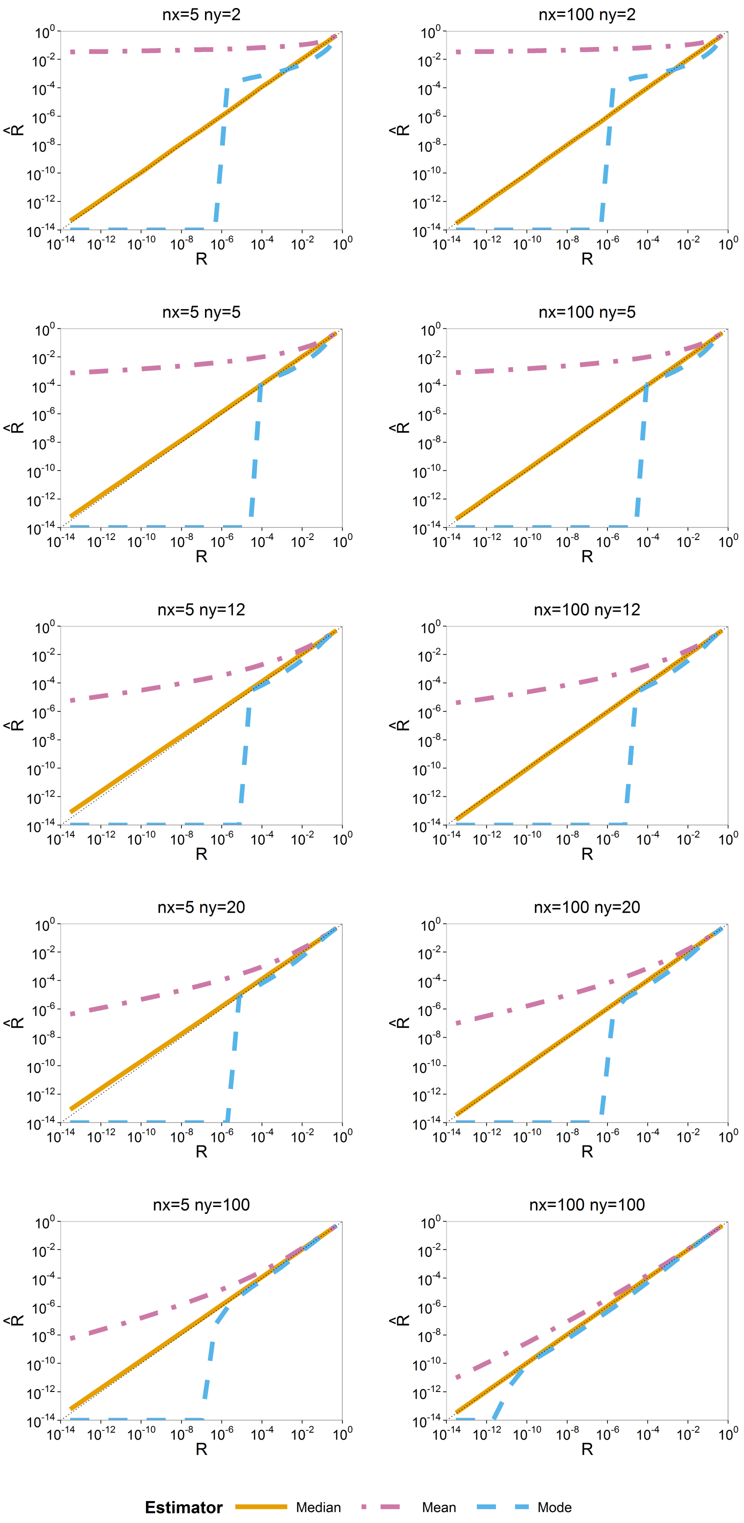

Figure S21: The 0.025 and 0.975 quantiles of the sampling distribution of the three Bayesian point estimators (mean, median and mode) calculated on the original scale and plotted on log_{10}-scale. When plotted on log_{10}-scale, a zero mode becomes $-\infty$. The diagonal dotted line represents the values where $\hat{R} = R$.

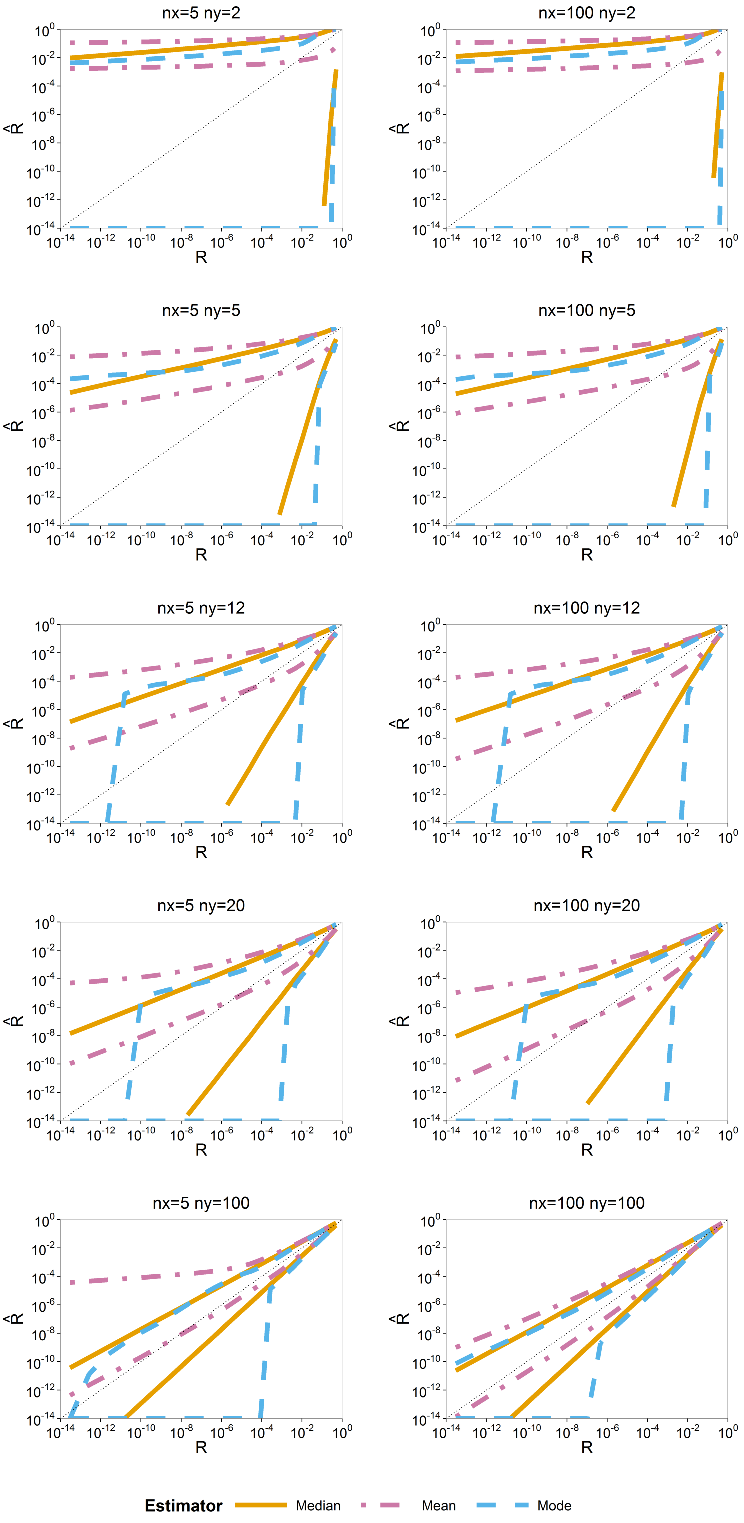

Figure S22: The median of the sampling distribution of the three Bayesian point estimators (mean, median and mode) calculated on the probit scale. The diagonal dotted line represents the values where $probit(\hat{R}) = probit(R)$.

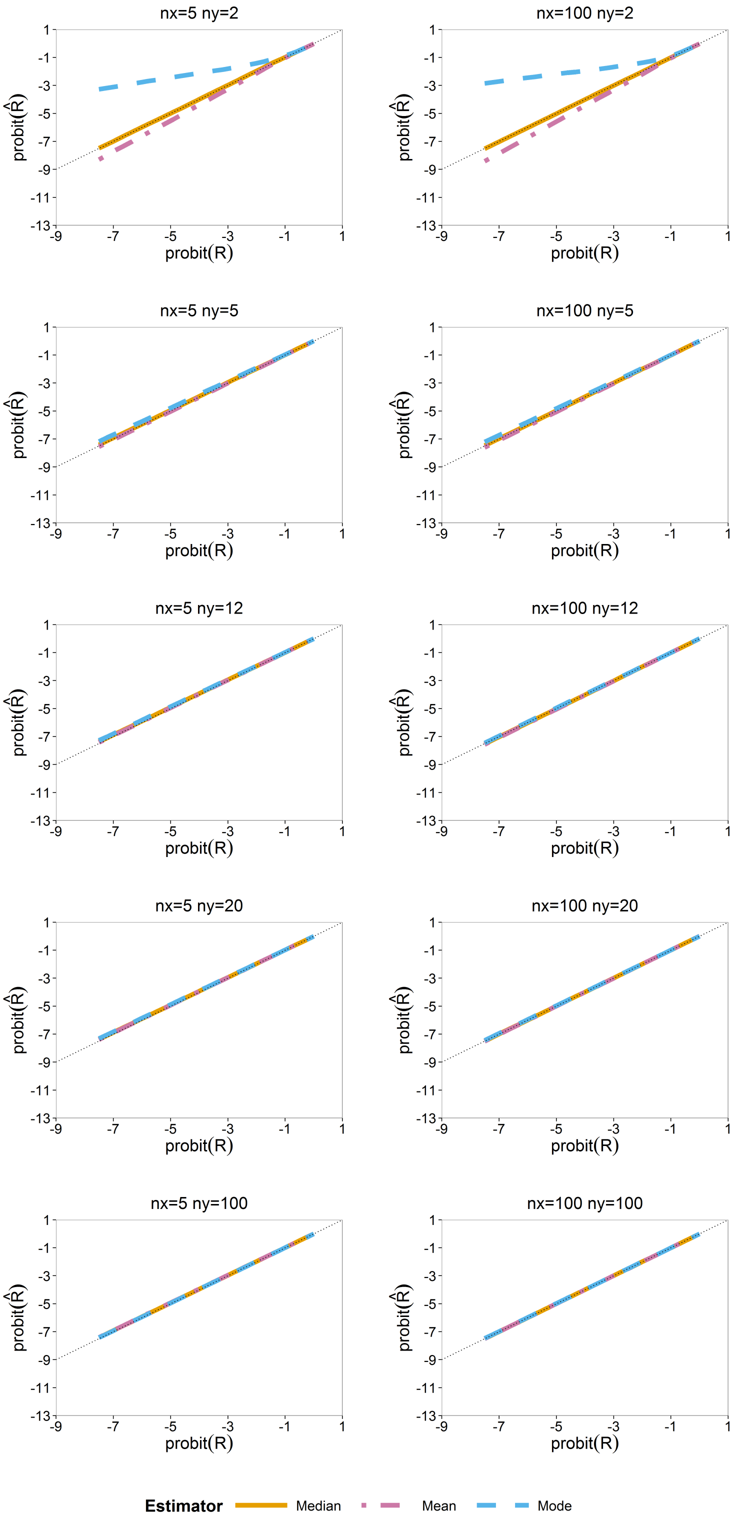

Figure S23: The 0.025 and 0.975 quantiles of the sampling distribution of the three Bayesian point estimators (mean, median and mode) calculated on the probit scale. The diagonal dotted line represents the values where $probit(\hat{R}) = probit(R)$.

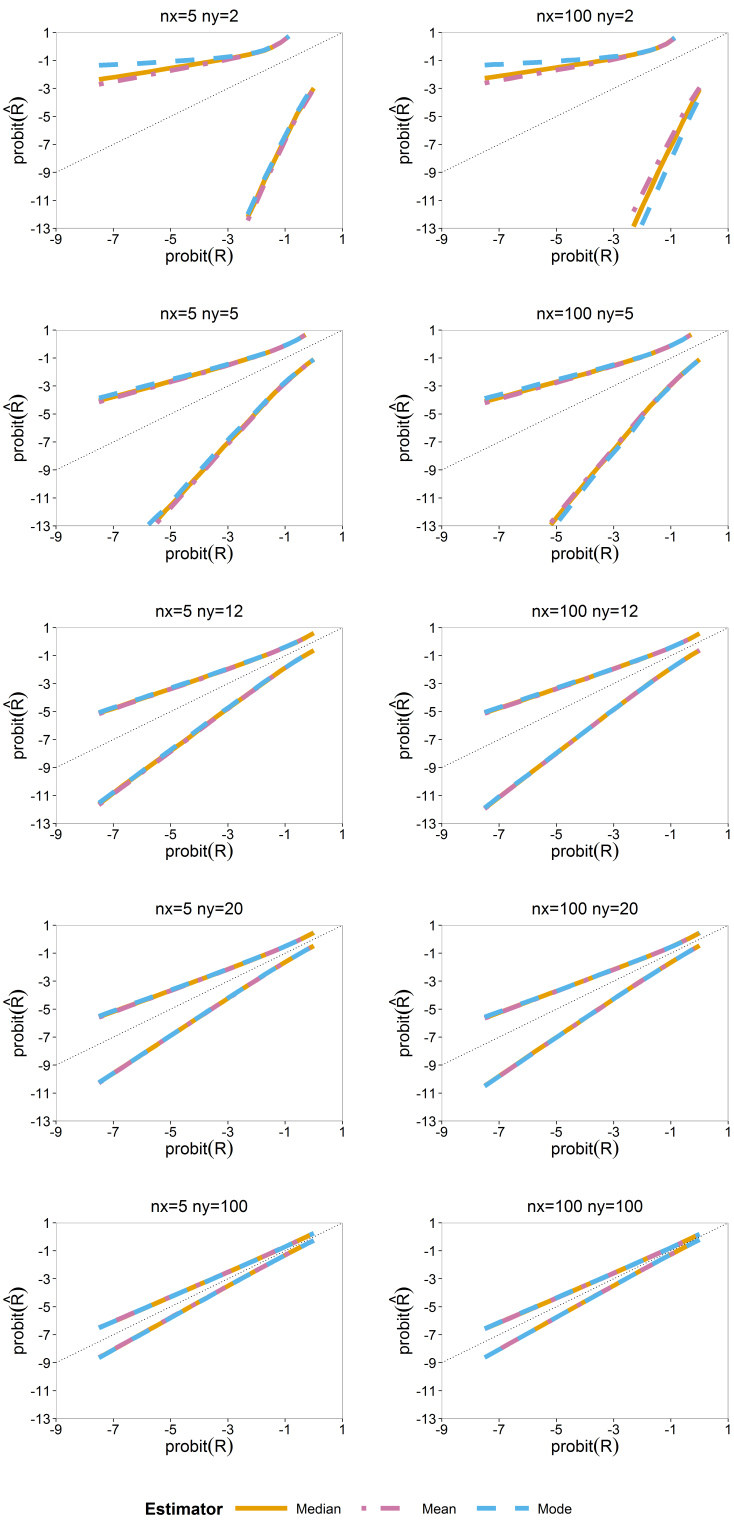

Figure S24: The median of the sampling distribution of the four point estimators, $\hat{R}_{MLE}$, $\hat{R}_{QMLE}$, $\hat{R}_{Bayes}$ and $\hat{R}_{NP}$ calculated on the probit scale. The diagonal dotted line represents the values where $probit(\hat{R}) = probit(R)$. The horizontal grey line gives the lower bound ($probit\left(\frac{1}{n_x n_y+2}\right)$) of $probit(\hat{R}_{NP})$.

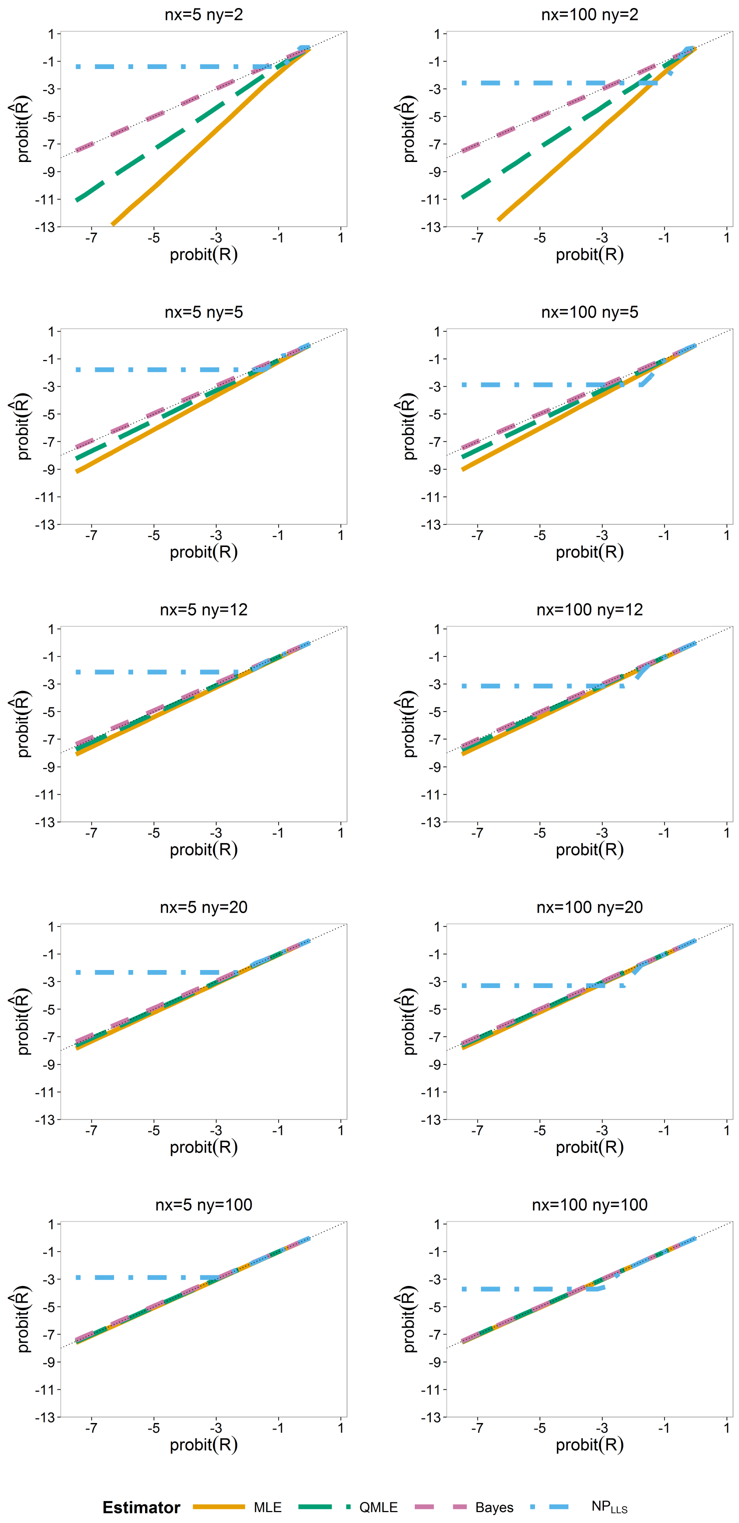

Figure S25: The 0.025 and 0.975 quantiles of the sampling distribution of the four point estimators, $\hat{R}_{MLE}$, $\hat{R}_{QMLE}$, $\hat{R}_{Bayes}$ and $\hat{R}_{NP}$ calculated on the probit scale. The diagonal dotted line represents the values where $probit(\hat{R}) = probit(R)$. The horizontal grey line gives the lower bound ($probit\left(\frac{1}{n_x n_y+2}\right)$) of $probit(\hat{R}_{NP})$.

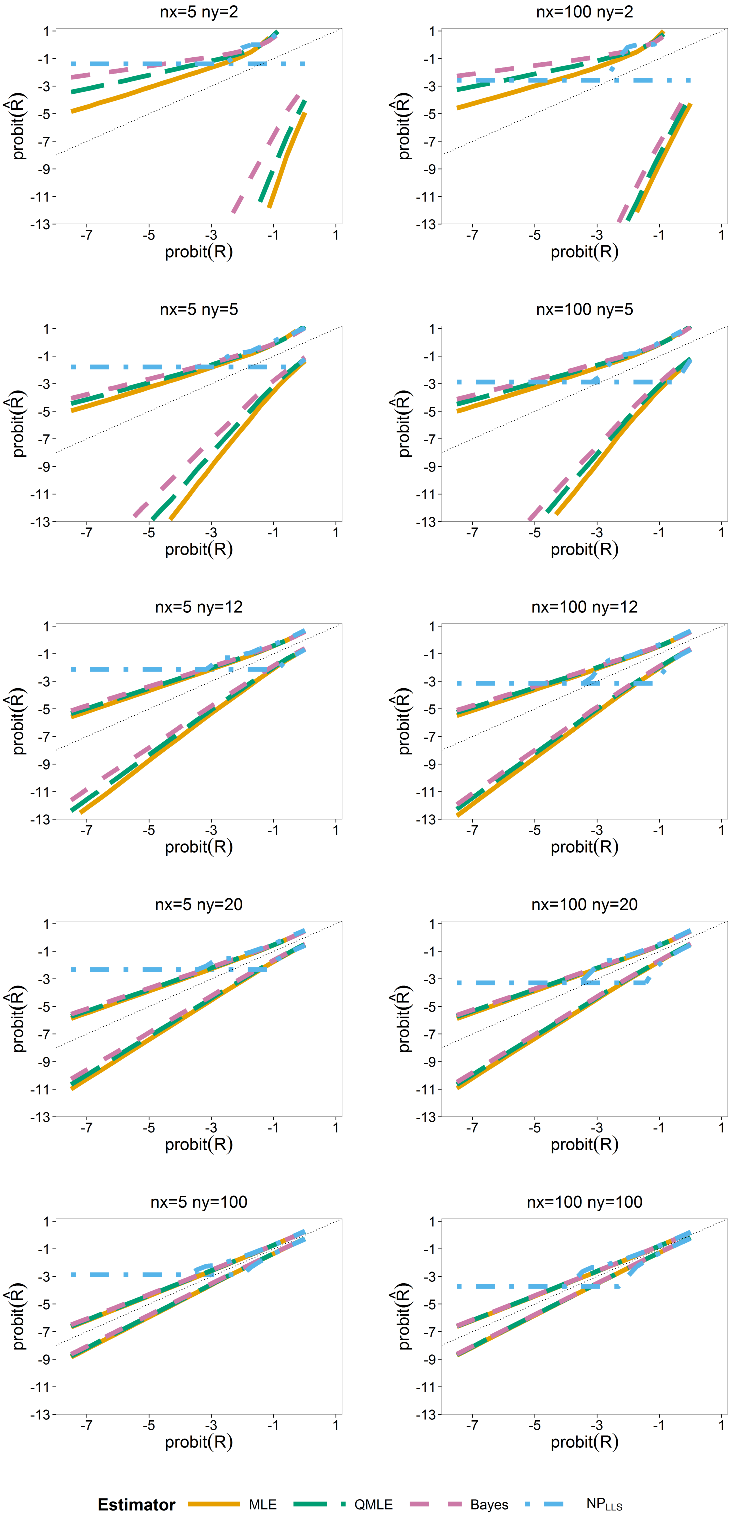

Figure S26: Scatterplots of the 90% two-sided coverage probabilities against the relative median interval length calculated on the probit scale. The value of the true R value is illustrated by the color scale. The size of the dots corresponds to the size of the sample size of effect. A vertical reference line is drawn at a coverage probability of 90%. The points corresponding to $n_y = 12$ are indicated by an open black circle.

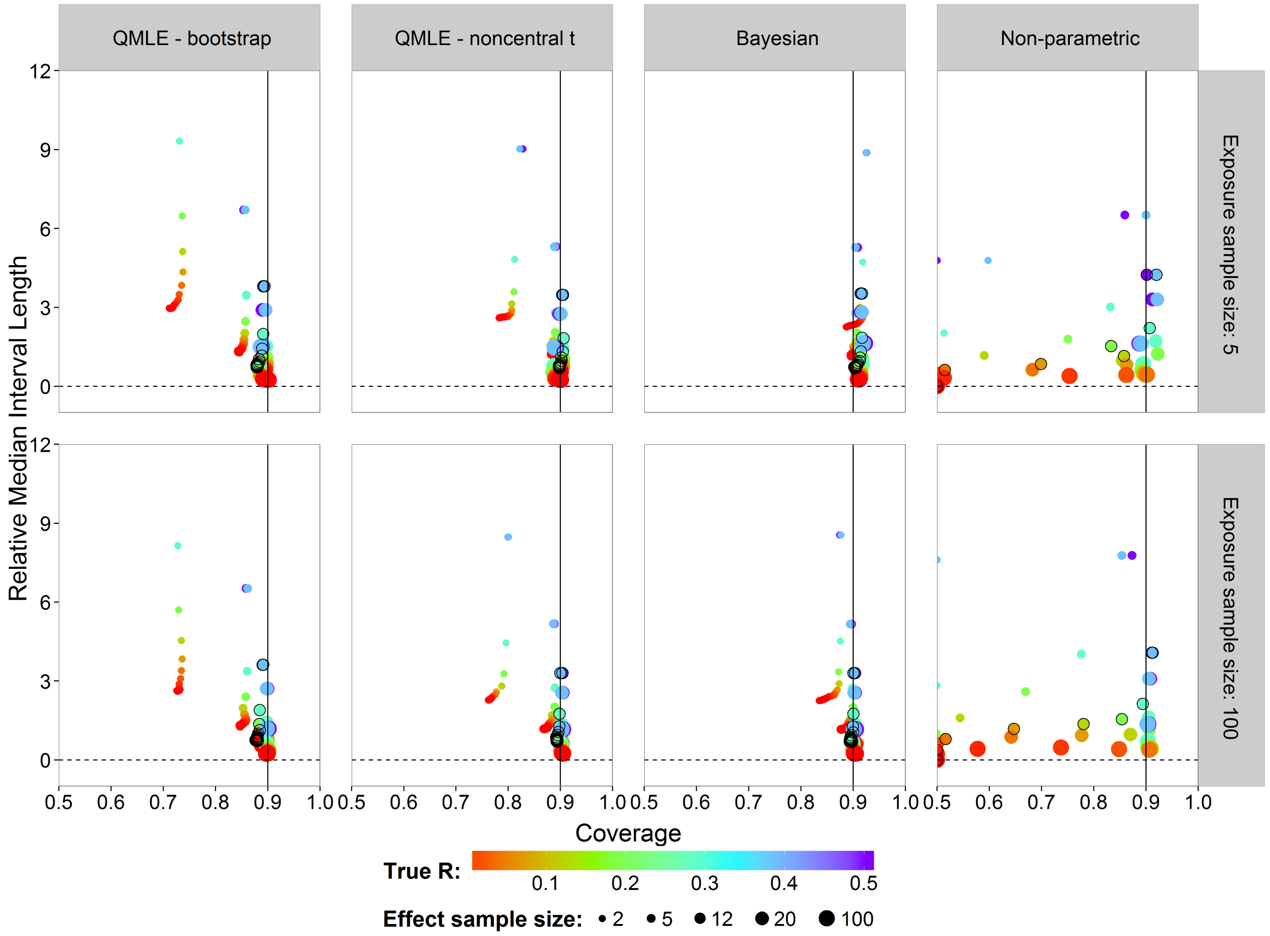

Figure S27: Scatterplots of the 95% one-sided coverage probabilities against the relative median difference calculated on the probit scale. The value of the true R value is illustrated by the color scale. The size of the dots corresponds to the size of the sample size of effect. A vertical reference line is drawn at a coverage probability of 95%. The points corresponding to $n_y = 12$ are indicated by a black circle.

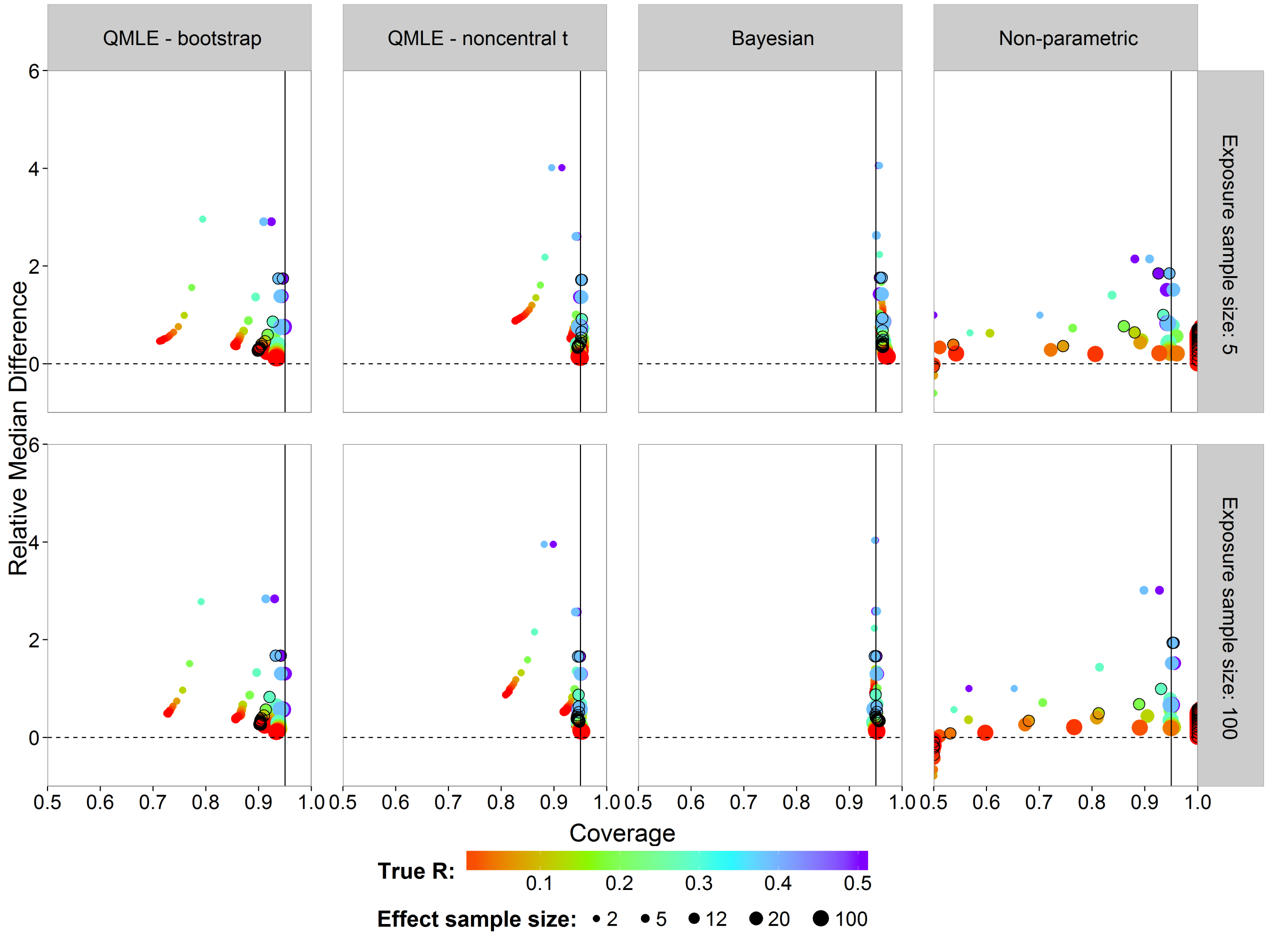

Table S10: The sample size, true R, true $probit(R)$, mean ($\bar{\hat{R}}$), standard deviation (SD), bias and root mean squared error (RMSE) of the MLE for all sample size scenarios and selected R values.

(n_x, n_y)	R	$probit(R)$	$\bar{\hat{R}}$ (SD)	bias	RMSE
(5,2)	0.5000	0.0000	-0.0307 (2.4070)	−0.0307	2.4072
	0.1240	−1.1551	-3.0883 (3.2728)	−1.9333	3.8012
	0.0047	−2.5989	-6.9104 (5.5870)	−4.3115	7.0571
	2.6e-05	−4.0427	-10.7324 (8.2261)	−6.6897	10.6028
	2.0e-08	−5.4865	-14.5545 (10.9578)	−9.0679	14.2232
	2.1e-12	−6.9304	-18.3765 (13.7270)	−11.4461	17.8730
(5,5)	0.5000	0.0000	-0.0019 (0.6570)	−0.0019	0.6570
	0.1240	−1.1551	-1.5759 (0.9441)	−0.4208	1.0337
	0.0047	−2.5989	-3.5433 (1.6449)	−0.9444	1.8968
	2.6e-05	−4.0427	-5.5108 (2.4286)	−1.4681	2.8378
	2.0e-08	−5.4865	-7.4783 (3.2353)	−1.9917	3.7993
	2.1e-12	−6.9304	-9.4457 (4.0515)	−2.5153	4.7688
(5,12)	0.5000	0.0000	-0.0011 (0.3369)	−0.0011	0.3369
	0.1240	−1.1551	-1.2954 (0.4476)	−0.1404	0.4691
	0.0047	−2.5989	-2.9134 (0.7443)	−0.3145	0.8080
	2.6e-05	−4.0427	-4.5314 (1.0862)	−0.4887	1.1911
	2.0e-08	−5.4865	-6.1493 (1.4415)	−0.6628	1.5866
	2.1e-12	−6.9304	-7.7673 (1.8024)	−0.8369	1.9872
(5,20)	0.5000	0.0000	-0.0012 (0.2560)	−0.0012	0.2560
	0.1240	−1.1551	-1.2308 (0.3235)	−0.0757	0.3323
	0.0047	−2.5989	-2.7678 (0.5181)	−0.1689	0.5449
	2.6e-05	−4.0427	-4.3048 (0.7482)	−0.2620	0.7928
	2.0e-08	−5.4865	-5.8417 (0.9893)	−0.3552	1.0512
	2.1e-12	−6.9304	-7.3787 (1.2350)	−0.4484	1.3139
(5,100)	0.5000	0.0000	0.0021 (0.1341)	0.0021	0.1341
	0.1240	−1.1551	-1.1728 (0.1603)	−0.0177	0.1613
	0.0047	−2.5989	-2.6414 (0.2338)	−0.0425	0.2376
	2.6e-05	−4.0427	-4.1100 (0.3241)	−0.0673	0.3310
	2.0e-08	−5.4865	-5.5786 (0.4206)	−0.0921	0.4306
	2.1e-12	−6.9304	-7.0472 (0.5198)	−0.1168	0.5328
(100,2)	0.5000	0.0000	-0.0377 (1.8901)	−0.0377	1.8905
	0.1240	−1.1551	-2.7841 (2.4844)	−1.6290	2.9708
	0.0047	−2.5989	-6.2170 (4.0743)	−3.6181	5.4489
	2.6e-05	−4.0427	-9.6499 (5.9165)	−5.6072	8.1514
	2.0e-08	−5.4865	-13.0828 (7.8348)	−7.5963	10.9128
	2.1e-12	−6.9304	-16.5157 (9.7847)	−9.5854	13.6975
(100,5)	0.5000	0.0000	0.0036 (0.6321)	0.0036	0.6321
	0.1240	−1.1551	-1.5439 (0.8846)	−0.3889	0.9663
	0.0047	−2.5989	-3.4783 (1.5562)	−0.8794	1.7875
	2.6e-05	−4.0427	-5.4127 (2.3131)	−1.3700	2.6884
	2.0e-08	−5.4865	-7.3470 (3.0933)	−1.8605	3.6097
	2.1e-12	−6.9304	-9.2814 (3.8828)	−2.3510	4.5391
(100,12)	0.5000	0.0000	-0.0015 (0.3186)	−0.0015	0.3186
	0.1240	−1.1551	-1.2845 (0.4293)	−0.1294	0.4484
	0.0047	−2.5989	-2.8883 (0.7150)	−0.2894	0.7713
	2.6e-05	−4.0427	-4.4920 (1.0422)	−0.4493	1.1350
	2.0e-08	−5.4865	-6.0958 (1.3819)	−0.6092	1.5102
	2.1e-12	−6.9304	-7.6995 (1.7266)	−0.7691	1.8901
(100,20)	0.5000	0.0000	0.0037 (0.2356)	0.0037	0.2356
	0.1240	−1.1551	-1.2256 (0.3101)	−0.0706	0.3180
	0.0047	−2.5989	-2.7623 (0.5067)	−0.1634	0.5324
	2.6e-05	−4.0427	-4.2990 (0.7344)	−0.2563	0.7778
	2.0e-08	−5.4865	-5.8357 (0.9716)	−0.3491	1.0324
	2.1e-12	−6.9304	-7.3724 (1.2126)	−0.4420	1.2907
(100,100)	0.5000	0.0000	-0.0024 (0.1003)	−0.0024	0.1004
	0.1240	−1.1551	-1.1699 (0.1286)	−0.0148	0.1294
	0.0047	−2.5989	-2.6292 (0.2049)	−0.0303	0.2071
	2.6e-05	−4.0427	-4.0886 (0.2945)	−0.0459	0.2981
	2.0e-08	−5.4865	-5.5479 (0.3883)	−0.0614	0.3932
	2.1e-12	−6.9304	-7.0073 (0.4839)	−0.0769	0.4900

Table S11: The sample size, true R, true $probit(R)$, mean $(\bar{\hat{R}})$, standard deviation (SD), bias and root mean squared error (RMSE) of the QMLE for all sample size scenarios and selected R values.

(n_x, n_y)	R	$probit(R)$	$\bar{\hat{R}}$ (SD)	bias	RMSE
(5,2)	0.5000	0.0000	-0.0215 (1.9343)	−0.0215	1.9344
	0.1240	−1.1551	-2.3912 (2.7028)	−1.2362	2.9721
	0.0047	−2.5989	-5.3534 (4.7159)	−2.7545	5.4615
	2.6e-05	−4.0427	-8.3156 (6.9871)	−4.2729	8.1901
	2.0e-08	−5.4865	-11.2779 (9.3298)	−5.7913	10.9811
	2.1e-12	−6.9304	-14.2401 (11.7010)	−7.3097	13.7966
(5,5)	0.5000	0.0000	-0.0017 (0.5877)	−0.0017	0.5877
	0.1240	−1.1551	-1.4095 (0.8445)	−0.2544	0.8820
	0.0047	−2.5989	-3.1693 (1.4713)	−0.5704	1.5780
	2.6e-05	−4.0427	-4.9290 (2.1722)	−0.8863	2.3460
	2.0e-08	−5.4865	-6.6888 (2.8938)	−1.2022	3.1336
	2.1e-12	−6.9304	-8.4485 (3.6238)	−1.5181	3.9289
(5,12)	0.5000	0.0000	-0.0010 (0.3215)	−0.0010	0.3215
	0.1240	−1.1551	-1.2364 (0.4259)	−0.0813	0.4335
	0.0047	−2.5989	-2.7805 (0.7064)	−0.1817	0.7294
	2.6e-05	−4.0427	-4.3247 (1.0303)	−0.2820	1.0682
	2.0e-08	−5.4865	-5.8689 (1.3669)	−0.3824	1.4194
	2.1e-12	−6.9304	-7.4131 (1.7088)	−0.4827	1.7757
(5,20)	0.5000	0.0000	-0.0012 (0.2486)	−0.0012	0.2487
	0.1240	−1.1551	-1.1955 (0.3135)	−0.0404	0.3161
	0.0047	−2.5989	-2.6884 (0.5010)	−0.0895	0.5090
	2.6e-05	−4.0427	-4.1814 (0.7230)	−0.1386	0.7362
	2.0e-08	−5.4865	-5.6743 (0.9557)	−0.1877	0.9740
	2.1e-12	−6.9304	-7.1672 (1.1929)	−0.2368	1.2162
(5,100)	0.5000	0.0000	0.0021 (0.1329)	0.0021	0.1329
	0.1240	−1.1551	-1.1626 (0.1588)	−0.0075	0.1590
	0.0047	−2.5989	-2.6184 (0.2316)	−0.0195	0.2324
	2.6e-05	−4.0427	-4.0742 (0.3211)	−0.0315	0.3227
	2.0e-08	−5.4865	-5.5300 (0.4167)	−0.0435	0.4190
	2.1e-12	−6.9304	-6.9858 (0.5150)	−0.0555	0.5180
(100,2)	0.5000	0.0000	-0.0292 (1.6350)	−0.0292	1.6352
	0.1240	−1.1551	-2.2766 (2.2785)	−1.1216	2.5396
	0.0047	−2.5989	-5.0859 (3.9147)	−2.4870	4.6379
	2.6e-05	−4.0427	-7.8952 (5.7636)	−3.8524	6.9325
	2.0e-08	−5.4865	-10.7044 (7.6729)	−5.2179	9.2790
	2.1e-12	−6.9304	-13.5137 (9.6068)	−6.5833	11.6461
(100,5)	0.5000	0.0000	0.0034 (0.5742)	0.0034	0.5742
	0.1240	−1.1551	-1.3960 (0.8145)	−0.2410	0.8494
	0.0047	−2.5989	-3.1453 (1.4467)	−0.5465	1.5465
	2.6e-05	−4.0427	-4.8946 (2.1558)	−0.8519	2.3180
	2.0e-08	−5.4865	-6.6439 (2.8856)	−1.1574	3.1091
	2.1e-12	−6.9304	-8.3932 (3.6237)	−1.4628	3.9078
(100,12)	0.5000	0.0000	-0.0015 (0.3057)	−0.0015	0.3057
	0.1240	−1.1551	-1.2323 (0.4127)	−0.0772	0.4199
	0.0047	−2.5989	-2.7709 (0.6885)	−0.1720	0.7097
	2.6e-05	−4.0427	-4.3094 (1.0042)	−0.2667	1.0390
	2.0e-08	−5.4865	-5.8480 (1.3317)	−0.3615	1.3799
	2.1e-12	−6.9304	-7.3866 (1.6640)	−0.4562	1.7254
(100,20)	0.5000	0.0000	0.0036 (0.2299)	0.0036	0.2299
	0.1240	−1.1551	-1.1957 (0.3028)	−0.0406	0.3055
	0.0047	−2.5989	-2.6949 (0.4952)	−0.0960	0.5044
	2.6e-05	−4.0427	-4.1941 (0.7178)	−0.1514	0.7336
	2.0e-08	−5.4865	-5.6933 (0.9497)	−0.2067	0.9719
	2.1e-12	−6.9304	-7.1925 (1.1854)	−0.2621	1.2140
(100,100)	0.5000	0.0000	-0.0024 (0.0998)	−0.0024	0.0998
	0.1240	−1.1551	-1.1640 (0.1279)	−0.0089	0.1282
	0.0047	−2.5989	-2.6160 (0.2039)	−0.0171	0.2046
	2.6e-05	−4.0427	-4.0681 (0.2931)	−0.0254	0.2942
	2.0e-08	−5.4865	-5.5201 (0.3864)	−0.0336	0.3879
	2.1e-12	−6.9304	-6.9722 (0.4815)	−0.0418	0.4833

Table S12: The sample size, true R, true $probit(R)$, mean $(\bar{\hat{R}})$, standard deviation (SD), bias and root mean squared error (RMSE) of the Bayes estimator for all sample size scenarios and selected R values.

(n_x, n_y)	R	$probit(R)$	$\bar{\hat{R}}$ (SD)	bias	RMSE
(5,2)	0.5000	0.0000	-0.0147 (1.4158)	−0.0147	1.4159
	0.1240	−1.1551	-1.7090 (1.9791)	−0.5539	2.0551
	0.0047	−2.5989	-3.7772 (3.4930)	−1.1783	3.6864
	2.6e-05	−4.0427	-5.8375 (5.2056)	−1.7948	5.5064
	2.0e-08	−5.4865	-7.9007 (6.9669)	−2.4142	7.3734
	2.1e-12	−6.9304	-9.9670 (8.7480)	−3.0366	9.2600
(5,5)	0.5000	0.0000	-0.0011 (0.5290)	−0.0011	0.5290
	0.1240	−1.1551	-1.2708 (0.7376)	−0.1157	0.7466
	0.0047	−2.5989	-2.8446 (1.2681)	−0.2457	1.2917
	2.6e-05	−4.0427	-4.4173 (1.8678)	−0.3746	1.9050
	2.0e-08	−5.4865	-5.9906 (2.4865)	−0.5041	2.5371
	2.1e-12	−6.9304	-7.5646 (3.1131)	−0.6342	3.1770
(5,12)	0.5000	0.0000	-0.0011 (0.3072)	−0.0011	0.3072
	0.1240	−1.1551	-1.1812 (0.4009)	−0.0261	0.4017
	0.0047	−2.5989	-2.6515 (0.6577)	−0.0526	0.6598
	2.6e-05	−4.0427	-4.1214 (0.9565)	−0.0787	0.9597
	2.0e-08	−5.4865	-5.5916 (1.2677)	−0.1050	1.2720
	2.1e-12	−6.9304	-7.0619 (1.5841)	−0.1316	1.5895
(5,20)	0.5000	0.0000	-0.0011 (0.2410)	−0.0011	0.2410
	0.1240	−1.1551	-1.1583 (0.3012)	−0.0032	0.3012
	0.0047	−2.5989	-2.6017 (0.4775)	−0.0028	0.4775
	2.6e-05	−4.0427	-4.0447 (0.6874)	−0.0020	0.6874
	2.0e-08	−5.4865	-5.4878 (0.9079)	−0.0012	0.9079
	2.1e-12	−6.9304	-6.9310 (1.1328)	−0.0006	1.1328
(5,100)	0.5000	0.0000	0.0021 (0.1311)	0.0021	0.1311
	0.1240	−1.1551	-1.1464 (0.1567)	0.0087	0.1569
	0.0047	−2.5989	-2.5800 (0.2285)	0.0189	0.2293
	2.6e-05	−4.0427	-4.0130 (0.3170)	0.0297	0.3184
	2.0e-08	−5.4865	-5.4460 (0.4116)	0.0406	0.4136
	2.1e-12	−6.9304	-6.8790 (0.5088)	0.0514	0.5114
(100,2)	0.5000	0.0000	-0.0219 (1.3801)	−0.0219	1.3803
	0.1240	−1.1551	-1.8086 (1.9961)	−0.6535	2.1004
	0.0047	−2.5989	-3.9864 (3.5615)	−1.3875	3.8223
	2.6e-05	−4.0427	-6.1569 (5.3120)	−2.1142	5.7173
	2.0e-08	−5.4865	-8.3308 (7.1083)	−2.8442	7.6562
	2.1e-12	−6.9304	-10.5074 (8.9216)	−3.5770	9.6120
(100,5)	0.5000	0.0000	0.0032 (0.5371)	0.0032	0.5371
	0.1240	−1.1551	-1.2987 (0.7590)	−0.1436	0.7725
	0.0047	−2.5989	-2.9105 (1.3548)	−0.3116	1.3901
	2.6e-05	−4.0427	-4.5213 (2.0237)	−0.4786	2.0796
	2.0e-08	−5.4865	-6.1332 (2.7120)	−0.6466	2.7880
	2.1e-12	−6.9304	-7.7455 (3.4074)	−0.8151	3.5035
(100,12)	0.5000	0.0000	-0.0014 (0.2984)	−0.0014	0.2984
	0.1240	−1.1551	-1.2003 (0.4010)	−0.0452	0.4036
	0.0047	−2.5989	-2.6930 (0.6685)	−0.0941	0.6751
	2.6e-05	−4.0427	-4.1854 (0.9756)	−0.1427	0.9860
	2.0e-08	−5.4865	-5.6781 (1.2943)	−0.1916	1.3084
	2.1e-12	−6.9304	-7.1710 (1.6176)	−0.2406	1.6354
(100,20)	0.5000	0.0000	0.0036 (0.2267)	0.0036	0.2267
	0.1240	−1.1551	-1.1775 (0.2976)	−0.0224	0.2985
	0.0047	−2.5989	-2.6504 (0.4865)	−0.0515	0.4892
	2.6e-05	−4.0427	-4.1232 (0.7055)	−0.0804	0.7101
	2.0e-08	−5.4865	-5.5961 (0.9336)	−0.1096	0.9400
	2.1e-12	−6.9304	-7.0691 (1.1654)	−0.1388	1.1737
(100,100)	0.5000	0.0000	-0.0023 (0.0995)	−0.0023	0.0995
	0.1240	−1.1551	-1.1601 (0.1275)	−0.0051	0.1276
	0.0047	−2.5989	-2.6068 (0.2031)	−0.0079	0.2033
	2.6e-05	−4.0427	-4.0534 (0.2919)	−0.0107	0.2921
	2.0e-08	−5.4865	-5.5001 (0.3849)	−0.0135	0.3851
	2.1e-12	−6.9304	-6.9467 (0.4795)	−0.0164	0.4798

Table S13: The sample size, true R, true $probit(R)$, mean $(\bar{\hat{R}})$, standard deviation (SD), bias and root mean squared error (RMSE) of the non-parametric estimator for all sample size scenarios and selected R values.

(n_x, n_y)	R	$probit(R)$	$\bar{\hat{R}}$ (SD)	bias	RMSE
(5,2)	0.5000	0.0000	-0.0261 (0.8929)	−0.0261	0.8933
	0.1240	−1.1551	-1.0225 (0.6026)	0.1326	0.6170
	0.0047	−2.5989	-1.3681 (0.1271)	1.2308	1.2373
	2.6e-05	−4.0427	-1.3830 (0.0000)	2.6597	2.6597
	2.0e-08	−5.4865	-1.3830 (0.0000)	4.1035	4.1035
	2.1e-12	−6.9304	-1.3830 (0.0000)	5.5474	5.5474
(5,5)	0.5000	0.0000	0.0026 (0.5799)	0.0026	0.5799
	0.1240	−1.1551	-1.1911 (0.5640)	−0.0361	0.5651
	0.0047	−2.5989	-1.7581 (0.1467)	0.8408	0.8535
	2.6e-05	−4.0427	-1.7862 (0.0000)	2.2566	2.2566
	2.0e-08	−5.4865	-1.7862 (0.0000)	3.7004	3.7004
	2.1e-12	−6.9304	-1.7862 (0.0000)	5.1442	5.1442
(5,12)	0.5000	0.0000	-0.0035 (0.3534)	−0.0035	0.3534
	0.1240	−1.1551	-1.2064 (0.4607)	−0.0513	0.4635
	0.0047	−2.5989	-2.0841 (0.1888)	0.5148	0.5483
	2.6e-05	−4.0427	-2.1408 (0.0145)	1.9019	1.9020
	2.0e-08	−5.4865	-2.1412 (0.0000)	3.3453	3.3453
	2.1e-12	−6.9304	-2.1412 (0.0000)	4.7892	4.7892
(5,20)	0.5000	0.0000	-0.0033 (0.2764)	−0.0033	0.2764
	0.1240	−1.1551	-1.1905 (0.3691)	−0.0354	0.3708
	0.0047	−2.5989	-2.2486 (0.2141)	0.3503	0.4106
	2.6e-05	−4.0427	-2.3335 (0.0143)	1.7092	1.7093
	2.0e-08	−5.4865	-2.3338 (0.0000)	3.1528	3.1528
	2.1e-12	−6.9304	-2.3338 (0.0000)	4.5966	4.5966
(5,100)	0.5000	0.0000	0.0028 (0.1459)	0.0028	0.1459
	0.1240	−1.1551	-1.1636 (0.1776)	−0.0085	0.1778
	0.0047	−2.5989	-2.5939 (0.3022)	0.0050	0.3023
	2.6e-05	−4.0427	-2.8775 (0.0293)	1.1652	1.1656
	2.0e-08	−5.4865	-2.8794 (0.0000)	2.6071	2.6071
	2.1e-12	−6.9304	-2.8794 (0.0000)	4.0509	4.0509
(100,2)	0.5000	0.0000	-0.0252 (1.4283)	−0.0252	1.4285
	0.1240	−1.1551	-1.8277 (1.0598)	−0.6726	1.2552
	0.0047	−2.5989	-2.5385 (0.2605)	0.0603	0.2674
	2.6e-05	−4.0427	-2.5790 (0.0134)	1.4638	1.4638
	2.0e-08	−5.4865	-2.5793 (0.0000)	2.9073	2.9073
	2.1e-12	−6.9304	-2.5793 (0.0000)	4.3511	4.3511
(100,5)	0.5000	0.0000	0.0011 (0.6613)	0.0011	0.6613
	0.1240	−1.1551	-1.5977 (0.9460)	−0.4426	1.0444
	0.0047	−2.5989	-2.8005 (0.3241)	−0.2017	0.3817
	2.6e-05	−4.0427	-2.8791 (0.0150)	1.1636	1.1637
	2.0e-08	−5.4865	-2.8794 (0.0000)	2.6071	2.6071
	2.1e-12	−6.9304	-2.8794 (0.0000)	4.0509	4.0509
(100,12)	0.5000	0.0000	0.0015 (0.3454)	0.0015	0.3454
	0.1240	−1.1551	-1.3374 (0.6226)	−0.1824	0.6488
	0.0047	−2.5989	-2.9741 (0.4364)	−0.3752	0.5755
	2.6e-05	−4.0427	-3.1432 (0.0336)	0.8995	0.9001
	2.0e-08	−5.4865	-3.1445 (0.0000)	2.3421	2.3421
	2.1e-12	−6.9304	-3.1445 (0.0000)	3.7859	3.7859
(100,20)	0.5000	0.0000	0.0048 (0.2616)	0.0048	0.2617
	0.1240	−1.1551	-1.2350 (0.4117)	−0.0799	0.4194
	0.0047	−2.5989	-3.0492 (0.4806)	−0.4503	0.6586
	2.6e-05	−4.0427	-3.2890 (0.0387)	0.7537	0.7547
	2.0e-08	−5.4865	-3.2908 (0.0000)	2.1957	2.1957
	2.1e-12	−6.9304	-3.2908 (0.0000)	3.6396	3.6396
(100,100)	0.5000	0.0000	-0.0019 (0.1153)	−0.0019	0.1153
	0.1240	−1.1551	-1.1692 (0.1483)	−0.0141	0.1490
	0.0047	−2.5989	-2.9163 (0.5665)	−0.3174	0.6493
	2.6e-05	−4.0427	-3.7098 (0.0852)	0.3329	0.3437
	2.0e-08	−5.4865	-3.7191 (0.0000)	1.7675	1.7675
	2.1e-12	−6.9304	-3.7191 (0.0000)	3.2113	3.2113
